# Supplementary material for: Predicting cancer origins with a DNA methylation-based deep neural network model
Source: PLoS One. 2020 May 8;15(5):e0226461. doi: 10.1371/journal.pone.0226461 (PMC7209244; doi:10.1371/journal.pone.0226461)
Supplement: S1 Table — (DOCX) [file pone.0226461.s001.docx]

**S1 Table. Cancer origin predictions for 1468 patient samples from TCGA**

|  | **Patient** | **Diagnosis** | **Primary_site** | **Prediction** | **Correct** |
| --- | --- | --- | --- | --- | --- |
| 1 | TCGA-G7-6796 | Kidney Renal Papillary Cell Carcinoma | Kidney | Kidney | Yes |
| 2 | TCGA-CV-A6JN | Head and Neck Squamous Cell Carcinoma | Head and Neck | Head and Neck | Yes |
| 3 | TCGA-D5-6927 | Colon Adenocarcinoma | Colorectal | Colorectal | Yes |
| 4 | TCGA-DJ-A2PW | Thyroid Carcinoma | Thyroid | Thyroid | Yes |
| 5 | TCGA-A2-A0T4 | Breast Invasive Carcinoma | Breast | Breast | Yes |
| 6 | TCGA-DU-6392 | Brain Lower Grade Glioma | Brain | Brain | Yes |
| 7 | TCGA-S3-AA14 | Breast Invasive Carcinoma | Breast | Breast | Yes |
| 8 | TCGA-B0-5699 | Kidney Renal Clear Cell Carcinoma | Kidney | Kidney | Yes |
| 9 | TCGA-DD-AADK | Liver Hepatocellular Carcinoma | Liver | Liver | Yes |
| 10 | TCGA-A7-A4SA | Breast Invasive Carcinoma | Breast | Breast | Yes |
| 11 | TCGA-CC-A7IL | Liver Hepatocellular Carcinoma | Liver | Liver | Yes |
| 12 | TCGA-2Z-A9JG | Kidney Renal Papillary Cell Carcinoma | Brain | Brain | Yes |
| 13 | TCGA-CZ-4853 | Kidney Renal Clear Cell Carcinoma | Kidney | Kidney | Yes |
| 14 | TCGA-DD-A113 | Liver Hepatocellular Carcinoma | Liver | Liver | Yes |
| 15 | TCGA-FB-AAPS | Pancreatic Adenocarcinoma | Adrenal Gland | Adrenal Gland | Yes |
| 16 | TCGA-DW-5560 | Kidney Renal Papillary Cell Carcinoma | Kidney | Kidney | Yes |
| 17 | TCGA-CN-6992 | Head and Neck Squamous Cell Carcinoma | Head and Neck | Head and Neck | Yes |
| 18 | TCGA-DD-A1EJ | Liver Hepatocellular Carcinoma | Liver | Liver | Yes |
| 19 | TCGA-CN-5365 | Head and Neck Squamous Cell Carcinoma | Head and Neck | Head and Neck | Yes |
| 20 | TCGA-SX-A7SS | Kidney Renal Papillary Cell Carcinoma | Brain | Brain | Yes |
| 21 | TCGA-OR-A5LL | Adrenocortical Carcinoma | Adrenal Gland | Adrenal Gland | Yes |
| 22 | TCGA-AO-A0JM | Breast Invasive Carcinoma | Breast | Breast | Yes |
| 23 | TCGA-YU-A912 | Testicular Germ Cell Tumors | Thyroid | Thyroid | Yes |
| 24 | TCGA-CU-A72E | Bladder Urothelial Carcinoma | Bladder | Bladder | Yes |
| 25 | TCGA-CH-5745 | Prostate Adenocarcinoma | Prostate | Prostate | Yes |
| 26 | TCGA-WB-A80K | Pheochromocytoma and Paraganglioma | Prostate | Prostate | Yes |
| 27 | TCGA-BA-6869 | Head and Neck Squamous Cell Carcinoma | Head and Neck | Head and Neck | Yes |
| 28 | TCGA-FG-8182 | Brain Lower Grade Glioma | Brain | Brain | Yes |
| 29 | TCGA-HT-8558 | Brain Lower Grade Glioma | Brain | Brain | Yes |
| 30 | TCGA-IZ-8195 | Kidney Renal Papillary Cell Carcinoma | Kidney | Kidney | Yes |
| 31 | TCGA-DK-A1AC | Bladder Urothelial Carcinoma | Bladder | Bladder | Yes |
| 32 | TCGA-22-4593 | Lung Squamous Cell Carcinoma | Lung | Lung | Yes |
| 33 | TCGA-EJ-8469 | Prostate Adenocarcinoma | Prostate | Prostate | Yes |
| 34 | TCGA-FX-A3NK | Sarcoma | Skin | Skin | Yes |
| 35 | TCGA-OR-A5JO | Adrenocortical Carcinoma | Adrenal Gland | Adrenal Gland | Yes |
| 36 | TCGA-2J-AAB1 | Pancreatic Adenocarcinoma | Prostate | Prostate | Yes |
| 37 | TCGA-D6-A6ES | Head and Neck Squamous Cell Carcinoma | Head and Neck | Esophagus | No |
| 38 | TCGA-5M-AAT5 |  | Colorectal | Colorectal | Yes |
| 39 | TCGA-97-A4M0 | Lung Adenocarcinoma | Lung | Lung | Yes |
| 40 | TCGA-BH-A2L8 | Breast Invasive Carcinoma | Breast | Breast | Yes |
| 41 | TCGA-56-7222 | Lung Squamous Cell Carcinoma | Lung | Lung | Yes |
| 42 | TCGA-R8-A6YH |  | Liver | Liver | Yes |
| 43 | TCGA-DJ-A3V6 | Thyroid Carcinoma | Thyroid | Thyroid | Yes |
| 44 | TCGA-A2-A25D | Breast Invasive Carcinoma | Breast | Breast | Yes |
| 45 | TCGA-CV-7101 | Head and Neck Squamous Cell Carcinoma | Kidney | Kidney | Yes |
| 46 | TCGA-TQ-A8XE | Brain Lower Grade Glioma | Liver | Liver | Yes |
| 47 | TCGA-CN-5361 | Head and Neck Squamous Cell Carcinoma | Kidney | Kidney | Yes |
| 48 | TCGA-90-6837 | Lung Squamous Cell Carcinoma | Lung | Lung | Yes |
| 49 | TCGA-XN-A8T3 | Pancreatic Adenocarcinoma | Adrenal Gland | Adrenal Gland | Yes |
| 50 | TCGA-33-AASJ | Lung Squamous Cell Carcinoma | Pancreas | Pancreas | Yes |
| 51 | TCGA-DJ-A2PZ | Thyroid Carcinoma | Thymus | Thymus | Yes |
| 52 | TCGA-86-A4P7 | Lung Adenocarcinoma | Lung | Lung | Yes |
| 53 | TCGA-L9-A444 | Lung Adenocarcinoma | Lung | Lung | Yes |
| 54 | TCGA-HT-7689 | Brain Lower Grade Glioma | Brain | Brain | Yes |
| 55 | TCGA-44-6778 | Lung Adenocarcinoma | Lung | Lung | Yes |
| 56 | TCGA-QK-A8Z7 | Head and Neck Squamous Cell Carcinoma | Head and Neck | Head and Neck | Yes |
| 57 | TCGA-55-6983 | Lung Adenocarcinoma | Lung | Lung | Yes |
| 58 | TCGA-63-A5MW | Lung Squamous Cell Carcinoma | Lung | Lung | Yes |
| 59 | TCGA-WB-A81T | Pheochromocytoma and Paraganglioma | Prostate | Prostate | Yes |
| 60 | TCGA-50-5066 | Lung Adenocarcinoma | Lung | Lung | Yes |
| 61 | TCGA-KN-8435 | Kidney Chromophobe | Kidney | Kidney | Yes |
| 62 | TCGA-EM-A2OW | Thyroid Carcinoma | Thyroid | Thyroid | Yes |
| 63 | TCGA-CV-6441 | Head and Neck Squamous Cell Carcinoma | Kidney | Kidney | Yes |
| 64 | TCGA-AR-A1AN | Breast Invasive Carcinoma | Breast | Breast | Yes |
| 65 | TCGA-NJ-A4YG | Lung Adenocarcinoma | Lung | Lung | Yes |
| 66 | TCGA-OR-A5J4 | Adrenocortical Carcinoma | Adrenal Gland | Adrenal Gland | Yes |
| 67 | TCGA-RW-A68F | Pheochromocytoma and Paraganglioma | Prostate | Prostate | Yes |
| 68 | TCGA-G9-6373 | Prostate Adenocarcinoma | Prostate | Prostate | Yes |
| 69 | TCGA-J9-A8CK | Prostate Adenocarcinoma | Prostate | Prostate | Yes |
| 70 | TCGA-CV-5434 | Head and Neck Squamous Cell Carcinoma | Kidney | Kidney | Yes |
| 71 | TCGA-D5-6926 | Colon Adenocarcinoma | Colorectal | Colorectal | Yes |
| 72 | TCGA-OR-A5LS | Adrenocortical Carcinoma | Adrenal Gland | Adrenal Gland | Yes |
| 73 | TCGA-BC-A3KF | Liver Hepatocellular Carcinoma | Liver | Liver | Yes |
| 74 | TCGA-B0-4841 | Kidney Renal Clear Cell Carcinoma | Kidney | Kidney | Yes |
| 75 | TCGA-X6-A8C2 | Sarcoma | Skin | Skin | Yes |
| 76 | TCGA-DU-6406 | Brain Lower Grade Glioma | Brain | Brain | Yes |
| 77 | TCGA-39-5016 | Lung Squamous Cell Carcinoma | Lung | Lung | Yes |
| 78 | TCGA-43-A56U | Lung Squamous Cell Carcinoma | Lung | Lung | Yes |
| 79 | TCGA-DJ-A4UT | Thyroid Carcinoma | Thyroid | Thyroid | Yes |
| 80 | TCGA-XD-AAUH | Pancreatic Adenocarcinoma | Adrenal Gland | Adrenal Gland | Yes |
| 81 | TCGA-S9-A6TY | Brain Lower Grade Glioma | Brain | Brain | Yes |
| 82 | TCGA-AO-A03U | Breast Invasive Carcinoma | Breast | Breast | Yes |
| 83 | TCGA-DD-A3A0 | Liver Hepatocellular Carcinoma | Liver | Liver | Yes |
| 84 | TCGA-BR-8486 | Stomach Adenocarcinoma | Thyroid | Thyroid | Yes |
| 85 | TCGA-G7-A8LD | Kidney Renal Papillary Cell Carcinoma | Brain | Brain | Yes |
| 86 | TCGA-38-6178 | Lung Adenocarcinoma | Lung | Lung | Yes |
| 87 | TCGA-OR-A5JS | Adrenocortical Carcinoma | Adrenal Gland | Adrenal Gland | Yes |
| 88 | TCGA-FF-8061 | Lymphoid Neoplasm Diffuse Large B-cell Lymphoma | Esophagus | Stomach | No |
| 89 | TCGA-DU-A7TC | Brain Lower Grade Glioma | Brain | Brain | Yes |
| 90 | TCGA-BA-5556 | Head and Neck Squamous Cell Carcinoma | Head and Neck | Head and Neck | Yes |
| 91 | TCGA-LD-A9QF | Breast Invasive Carcinoma | Breast | Breast | Yes |
| 92 | TCGA-AF-2693 | Rectum Adenocarcinoma | Soft Tissue | Soft Tissue | Yes |
| 93 | TCGA-T1-A6J8 | Liver Hepatocellular Carcinoma | Liver | Liver | Yes |
| 94 | TCGA-G4-6293 | Colon Adenocarcinoma | Colorectal | Colorectal | Yes |
| 95 | TCGA-L9-A7SV | Lung Adenocarcinoma | Lung | Lung | Yes |
| 96 | TCGA-E8-A418 | Thyroid Carcinoma | Thymus | Thymus | Yes |
| 97 | TCGA-CH-5744 | Prostate Adenocarcinoma | Prostate | Prostate | Yes |
| 98 | TCGA-HT-7681 | Brain Lower Grade Glioma | Liver | Liver | Yes |
| 99 | TCGA-HT-7608 | Brain Lower Grade Glioma | Brain | Brain | Yes |
| 100 | TCGA-KS-A41L | Thyroid Carcinoma | Thyroid | Thyroid | Yes |
| 101 | TCGA-DD-AACX | Liver Hepatocellular Carcinoma | Lung | Lung | Yes |
| 102 | TCGA-56-8308 | Lung Squamous Cell Carcinoma | Pancreas | Pancreas | Yes |
| 103 | TCGA-E8-A413 | Thyroid Carcinoma | Thyroid | Thyroid | Yes |
| 104 | TCGA-L5-A88Y | Esophageal Carcinoma | Brain | Brain | Yes |
| 105 | TCGA-UF-A7JD | Head and Neck Squamous Cell Carcinoma | Head and Neck | Head and Neck | Yes |
| 106 | TCGA-HU-8245 | Stomach Adenocarcinoma | Stomach | Stomach | Yes |
| 107 | TCGA-LG-A9QD | Liver Hepatocellular Carcinoma | Liver | Liver | Yes |
| 108 | TCGA-DJ-A3VF | Thyroid Carcinoma | Thyroid | Thyroid | Yes |
| 109 | TCGA-33-AAS8 | Lung Squamous Cell Carcinoma | Lung | Lung | Yes |
| 110 | TCGA-DK-A3IU | Bladder Urothelial Carcinoma | Bladder | Bladder | Yes |
| 111 | TCGA-TM-A84I | Brain Lower Grade Glioma | Brain | Brain | Yes |
| 112 | TCGA-BF-A1Q0 | Skin Cutaneous Melanoma | Stomach | Stomach | Yes |
| 113 | TCGA-CK-6747 | Colon Adenocarcinoma | Colorectal | Colorectal | Yes |
| 114 | TCGA-RW-A67Y | Pheochromocytoma and Paraganglioma | Prostate | Prostate | Yes |
| 115 | TCGA-L5-A8NM | Esophageal Carcinoma | Esophagus | Esophagus | Yes |
| 116 | TCGA-E9-A22A | Breast Invasive Carcinoma | Breast | Breast | Yes |
| 117 | TCGA-E2-A108 | Breast Invasive Carcinoma | Breast | Breast | Yes |
| 118 | TCGA-W2-A7HH | Pheochromocytoma and Paraganglioma | Prostate | Prostate | Yes |
| 119 | TCGA-05-4396 | Lung Adenocarcinoma | Lung | Lung | Yes |
| 120 | TCGA-CD-8533 | Stomach Adenocarcinoma | Testis | Testis | Yes |
| 121 | TCGA-2L-AAQJ | Pancreatic Adenocarcinoma | Adrenal Gland | Adrenal Gland | Yes |
| 122 | TCGA-BH-A1FN | Breast Invasive Carcinoma | Breast | Breast | Yes |
| 123 | TCGA-D6-A6EP | Head and Neck Squamous Cell Carcinoma | Head and Neck | Head and Neck | Yes |
| 124 | TCGA-GR-7351 | Lymphoid Neoplasm Diffuse Large B-cell Lymphoma | Esophagus | Stomach | No |
| 125 | TCGA-B6-A0RI | Breast Invasive Carcinoma | Breast | Breast | Yes |
| 126 | TCGA-H9-7775 | Prostate Adenocarcinoma | Soft Tissue | Soft Tissue | Yes |
| 127 | TCGA-BB-4228 | Head and Neck Squamous Cell Carcinoma | Head and Neck | Head and Neck | Yes |
| 128 | TCGA-EJ-7794 | Prostate Adenocarcinoma | Prostate | Prostate | Yes |
| 129 | TCGA-73-4676 | Lung Adenocarcinoma | Lung | Lung | Yes |
| 130 | TCGA-BH-A0BC | Breast Invasive Carcinoma | Breast | Breast | Yes |
| 131 | TCGA-FD-A3B3 | Bladder Urothelial Carcinoma | Bladder | Bladder | Yes |
| 132 | TCGA-HV-A7OP | Pancreatic Adenocarcinoma | Adrenal Gland | Adrenal Gland | Yes |
| 133 | TCGA-AG-3725 | Rectum Adenocarcinoma | Soft Tissue | Soft Tissue | Yes |
| 134 | TCGA-S9-A7R3 | Brain Lower Grade Glioma | Brain | Brain | Yes |
| 135 | TCGA-DJ-A1QH | Thyroid Carcinoma | Thyroid | Thyroid | Yes |
| 136 | TCGA-BH-A0HY | Breast Invasive Carcinoma | Breast | Breast | Yes |
| 137 | TCGA-EW-A423 | Breast Invasive Carcinoma | Breast | Breast | Yes |
| 138 | TCGA-94-A5I4 | Lung Squamous Cell Carcinoma | Lung | Lung | Yes |
| 139 | TCGA-VR-AA4D | Esophageal Carcinoma | Brain | Brain | Yes |
| 140 | TCGA-HC-8258 | Prostate Adenocarcinoma | Prostate | Prostate | Yes |
| 141 | TCGA-FP-8209 | Stomach Adenocarcinoma | Thyroid | Thyroid | Yes |
| 142 | TCGA-D7-A74B | Stomach Adenocarcinoma | Stomach | Stomach | Yes |
| 143 | TCGA-BT-A20T | Bladder Urothelial Carcinoma | Bladder | Bladder | Yes |
| 144 | TCGA-NH-A8F7 | Colon Adenocarcinoma | Colorectal | Colorectal | Yes |
| 145 | TCGA-44-6147 | Lung Adenocarcinoma | Lung | Lung | Yes |
| 146 | TCGA-95-A4VN | Lung Adenocarcinoma | Lung | Lung | Yes |
| 147 | TCGA-MM-A563 | Kidney Renal Clear Cell Carcinoma | Kidney | Kidney | Yes |
| 148 | TCGA-BR-8361 | Stomach Adenocarcinoma | Stomach | Stomach | Yes |
| 149 | TCGA-DD-A11B | Liver Hepatocellular Carcinoma | Liver | Liver | Yes |
| 150 | TCGA-DQ-7592 | Head and Neck Squamous Cell Carcinoma | Kidney | Kidney | Yes |
| 151 | TCGA-VM-A8CE | Brain Lower Grade Glioma | Brain | Brain | Yes |
| 152 | TCGA-UZ-A9PK | Kidney Renal Papillary Cell Carcinoma | Kidney | Kidney | Yes |
| 153 | TCGA-2G-AAGC | Testicular Germ Cell Tumors | Thyroid | Thyroid | Yes |
| 154 | TCGA-2Z-A9J6 | Kidney Renal Papillary Cell Carcinoma | Kidney | Kidney | Yes |
| 155 | TCGA-KD-A5QT | Sarcoma | Skin | Skin | Yes |
| 156 | TCGA-63-A5MS | Lung Squamous Cell Carcinoma | Lung | Lung | Yes |
| 157 | TCGA-BR-6456 | Stomach Adenocarcinoma | Stomach | Stomach | Yes |
| 158 | TCGA-QQ-A8VB | Sarcoma | Skin | Skin | Yes |
| 159 | TCGA-CV-A6JE | Head and Neck Squamous Cell Carcinoma | Head and Neck | Head and Neck | Yes |
| 160 | TCGA-NC-A5HQ | Lung Squamous Cell Carcinoma | Lung | Lung | Yes |
| 161 | TCGA-CC-A3M9 | Liver Hepatocellular Carcinoma | Lung | Lung | Yes |
| 162 | TCGA-IM-A3U2 | Thyroid Carcinoma | Thymus | Thymus | Yes |
| 163 | TCGA-H4-A2HO | Bladder Urothelial Carcinoma | Bladder | Bladder | Yes |
| 164 | TCGA-AR-A0TW | Breast Invasive Carcinoma | Breast | Breast | Yes |
| 165 | TCGA-IM-A3EB | Thyroid Carcinoma | Thyroid | Thyroid | Yes |
| 166 | TCGA-34-8456 | Lung Squamous Cell Carcinoma | Lung | Lung | Yes |
| 167 | TCGA-E2-A109 | Breast Invasive Carcinoma | Breast | Breast | Yes |
| 168 | TCGA-BQ-5892 | Kidney Renal Papillary Cell Carcinoma | Brain | Brain | Yes |
| 169 | TCGA-5P-A9K3 | Kidney Renal Papillary Cell Carcinoma | Kidney | Kidney | Yes |
| 170 | TCGA-P5-A5F1 | Brain Lower Grade Glioma | Liver | Liver | Yes |
| 171 | TCGA-CC-5260 | Liver Hepatocellular Carcinoma | Liver | Liver | Yes |
| 172 | TCGA-BA-4078 | Head and Neck Squamous Cell Carcinoma | Head and Neck | Head and Neck | Yes |
| 173 | TCGA-S9-A6U2 | Brain Lower Grade Glioma | Liver | Liver | Yes |
| 174 | TCGA-AO-A1KR | Breast Invasive Carcinoma | Breast | Breast | Yes |
| 175 | TCGA-18-4721 | Lung Squamous Cell Carcinoma | Lung | Lung | Yes |
| 176 | TCGA-BP-5177 | Kidney Renal Clear Cell Carcinoma | Kidney | Kidney | Yes |
| 177 | TCGA-B0-4714 | Kidney Renal Clear Cell Carcinoma | Kidney | Kidney | Yes |
| 178 | TCGA-AF-A56L | Rectum Adenocarcinoma | Soft Tissue | Soft Tissue | Yes |
| 179 | TCGA-KL-8341 | Kidney Chromophobe | Kidney | Kidney | Yes |
| 180 | TCGA-BH-A0DQ | Breast Invasive Carcinoma | Breast | Breast | Yes |
| 181 | TCGA-AO-A03M | Breast Invasive Carcinoma | Breast | Breast | Yes |
| 182 | TCGA-VQ-A91U | Stomach Adenocarcinoma | Stomach | Stomach | Yes |
| 183 | TCGA-OR-A5JT | Adrenocortical Carcinoma | Adrenal Gland | Adrenal Gland | Yes |
| 184 | TCGA-55-7914 | Lung Adenocarcinoma | Lung | Lung | Yes |
| 185 | TCGA-PC-A5DP | Sarcoma | Skin | Skin | Yes |
| 186 | TCGA-B2-5635 | Kidney Renal Clear Cell Carcinoma | Kidney | Kidney | Yes |
| 187 | TCGA-CV-A6K0 | Head and Neck Squamous Cell Carcinoma | Head and Neck | Head and Neck | Yes |
| 188 | TCGA-CZ-5462 | Kidney Renal Clear Cell Carcinoma | Kidney | Kidney | Yes |
| 189 | TCGA-BP-5170 | Kidney Renal Clear Cell Carcinoma | Kidney | Kidney | Yes |
| 190 | TCGA-CV-7095 | Head and Neck Squamous Cell Carcinoma | Head and Neck | Head and Neck | Yes |
| 191 | TCGA-DU-5872 | Brain Lower Grade Glioma | Brain | Brain | Yes |
| 192 | TCGA-KO-8408 | Kidney Chromophobe | Kidney | Kidney | Yes |
| 193 | TCGA-62-A470 | Lung Adenocarcinoma | Lung | Lung | Yes |
| 194 | TCGA-RW-A67X | Pheochromocytoma and Paraganglioma | Prostate | Prostate | Yes |
| 195 | TCGA-BB-7863 | Head and Neck Squamous Cell Carcinoma | Kidney | Kidney | Yes |
| 196 | TCGA-EM-A22P | Thyroid Carcinoma | Thyroid | Thyroid | Yes |
| 197 | TCGA-D8-A1XZ | Breast Invasive Carcinoma | Breast | Breast | Yes |
| 198 | TCGA-77-8144 | Lung Squamous Cell Carcinoma | Lung | Lung | Yes |
| 199 | TCGA-BH-A0DH | Breast Invasive Carcinoma | Breast | Breast | Yes |
| 200 | TCGA-CJ-5679 | Kidney Renal Clear Cell Carcinoma | Kidney | Kidney | Yes |
| 201 | TCGA-FG-A60K | Brain Lower Grade Glioma | Brain | Brain | Yes |
| 202 | TCGA-D8-A1XV | Breast Invasive Carcinoma | Breast | Breast | Yes |
| 203 | TCGA-B0-5095 | Kidney Renal Clear Cell Carcinoma | Kidney | Kidney | Yes |
| 204 | TCGA-55-6980 | Lung Adenocarcinoma | Lung | Lung | Yes |
| 205 | TCGA-BR-6564 | Stomach Adenocarcinoma | Stomach | Stomach | Yes |
| 206 | TCGA-DX-AB2O | Sarcoma | Skin | Skin | Yes |
| 207 | TCGA-A6-2677 | Colon Adenocarcinoma | Esophagus | Esophagus | Yes |
| 208 | TCGA-D8-A4Z1 | Breast Invasive Carcinoma | Breast | Breast | Yes |
| 209 | TCGA-P3-A6T6 | Head and Neck Squamous Cell Carcinoma | Head and Neck | Head and Neck | Yes |
| 210 | TCGA-XF-A9T2 | Bladder Urothelial Carcinoma | Bladder | Bladder | Yes |
| 211 | TCGA-93-7348 | Lung Adenocarcinoma | Lung | Lung | Yes |
| 212 | TCGA-05-4384 | Lung Adenocarcinoma | Lung | Pancreas | No |
| 213 | TCGA-HU-8243 | Stomach Adenocarcinoma | Testis | Testis | Yes |
| 214 | TCGA-96-8170 | Lung Squamous Cell Carcinoma | Lung | Lung | Yes |
| 215 | TCGA-EJ-7781 | Prostate Adenocarcinoma | Soft Tissue | Soft Tissue | Yes |
| 216 | TCGA-85-A511 | Lung Squamous Cell Carcinoma | Lung | Lung | Yes |
| 217 | TCGA-75-5125 | Lung Adenocarcinoma | Lung | Lung | Yes |
| 218 | TCGA-DU-A7TB | Brain Lower Grade Glioma | Liver | Liver | Yes |
| 219 | TCGA-P8-A5KC | Pheochromocytoma and Paraganglioma | Prostate | Prostate | Yes |
| 220 | TCGA-BH-A6R9 | Breast Invasive Carcinoma | Breast | Breast | Yes |
| 221 | TCGA-C8-A26X | Breast Invasive Carcinoma | Breast | Breast | Yes |
| 222 | TCGA-DJ-A2PR | Thyroid Carcinoma | Thyroid | Thyroid | Yes |
| 223 | TCGA-18-5592 | Lung Squamous Cell Carcinoma | Lung | Lung | Yes |
| 224 | TCGA-MH-A562 | Kidney Renal Papillary Cell Carcinoma | Brain | Brain | Yes |
| 225 | TCGA-78-7536 | Lung Adenocarcinoma | Lung | Liver | No |
| 226 | TCGA-BR-A4PD | Stomach Adenocarcinoma | Testis | Testis | Yes |
| 227 | TCGA-RN-AAAQ | Sarcoma | Stomach | Stomach | Yes |
| 228 | TCGA-D7-8578 | Stomach Adenocarcinoma | Thyroid | Thyroid | Yes |
| 229 | TCGA-95-7567 | Lung Adenocarcinoma | Lung | Lung | Yes |
| 230 | TCGA-50-6595 | Lung Adenocarcinoma | Lung | Lung | Yes |
| 231 | TCGA-DX-AB2E | Sarcoma | Stomach | Stomach | Yes |
| 232 | TCGA-HW-A5KJ | Brain Lower Grade Glioma | Brain | Brain | Yes |
| 233 | TCGA-E2-A2P5 | Breast Invasive Carcinoma | Breast | Breast | Yes |
| 234 | TCGA-CV-5979 | Head and Neck Squamous Cell Carcinoma | Kidney | Kidney | Yes |
| 235 | TCGA-ZG-A9L5 | Prostate Adenocarcinoma | Prostate | Prostate | Yes |
| 236 | TCGA-BH-A1EN | Breast Invasive Carcinoma | Breast | Breast | Yes |
| 237 | TCGA-DE-A69J | Thyroid Carcinoma | Thyroid | Thyroid | Yes |
| 238 | TCGA-UF-A718 | Head and Neck Squamous Cell Carcinoma | Head and Neck | Head and Neck | Yes |
| 239 | TCGA-E2-A1IK | Breast Invasive Carcinoma | Breast | Breast | Yes |
| 240 | TCGA-QC-AA9N | Sarcoma | Soft Tissue | Soft Tissue | Yes |
| 241 | TCGA-KL-8326 | Kidney Chromophobe | Kidney | Kidney | Yes |
| 242 | TCGA-CR-7383 | Head and Neck Squamous Cell Carcinoma | Head and Neck | Head and Neck | Yes |
| 243 | TCGA-EI-6884 | Rectum Adenocarcinoma | Soft Tissue | Soft Tissue | Yes |
| 244 | TCGA-XF-A9SH | Bladder Urothelial Carcinoma | Bladder | Bladder | Yes |
| 245 | TCGA-BQ-5880 | Kidney Renal Papillary Cell Carcinoma | Brain | Brain | Yes |
| 246 | TCGA-XF-A9T8 | Bladder Urothelial Carcinoma | Bladder | Bladder | Yes |
| 247 | TCGA-D6-6824 | Head and Neck Squamous Cell Carcinoma | Kidney | Kidney | Yes |
| 248 | TCGA-B6-A1KC | Breast Invasive Carcinoma | Breast | Breast | Yes |
| 249 | TCGA-EL-A3H1 | Thyroid Carcinoma | Thyroid | Thyroid | Yes |
| 250 | TCGA-2G-AAHP | Testicular Germ Cell Tumors | Thyroid | Thyroid | Yes |
| 251 | TCGA-VQ-A91Q | Stomach Adenocarcinoma | Testis | Testis | Yes |
| 252 | TCGA-CV-6954 | Head and Neck Squamous Cell Carcinoma | Head and Neck | Head and Neck | Yes |
| 253 | TCGA-93-8067 | Lung Adenocarcinoma | Lung | Lung | Yes |
| 254 | TCGA-IE-A3OV | Sarcoma | Soft Tissue | Soft Tissue | Yes |
| 255 | TCGA-ZF-A9RC | Bladder Urothelial Carcinoma | Bladder | Bladder | Yes |
| 256 | TCGA-QH-A6X5 | Brain Lower Grade Glioma | Liver | Liver | Yes |
| 257 | TCGA-HC-7211 | Prostate Adenocarcinoma | Colorectal | Colorectal | Yes |
| 258 | TCGA-VW-A8FI | Brain Lower Grade Glioma | Brain | Brain | Yes |
| 259 | TCGA-A2-A0YJ | Breast Invasive Carcinoma | Breast | Breast | Yes |
| 260 | TCGA-J4-8200 | Prostate Adenocarcinoma | Prostate | Prostate | Yes |
| 261 | TCGA-P5-A5EV | Brain Lower Grade Glioma | Brain | Brain | Yes |
| 262 | TCGA-XF-A9SP | Bladder Urothelial Carcinoma | Bladder | Bladder | Yes |
| 263 | TCGA-78-7154 | Lung Adenocarcinoma | Lung | Lung | Yes |
| 264 | TCGA-AC-A62X | Breast Invasive Carcinoma | Breast | Breast | Yes |
| 265 | TCGA-DK-A2I4 | Bladder Urothelial Carcinoma | Bladder | Bladder | Yes |
| 266 | TCGA-KM-8439 | Kidney Chromophobe | Kidney | Kidney | Yes |
| 267 | TCGA-EW-A1P8 | Breast Invasive Carcinoma | Breast | Breast | Yes |
| 268 | TCGA-MH-A55W | Kidney Renal Papillary Cell Carcinoma | Kidney | Kidney | Yes |
| 269 | TCGA-MP-A4TE | Lung Adenocarcinoma | Lung | Lung | Yes |
| 270 | TCGA-DD-AAD8 | Liver Hepatocellular Carcinoma | Liver | Liver | Yes |
| 271 | TCGA-50-5045 | Lung Adenocarcinoma | Lung | Lung | Yes |
| 272 | TCGA-A2-A0CK | Breast Invasive Carcinoma | Breast | Breast | Yes |
| 273 | TCGA-3A-A9IN | Pancreatic Adenocarcinoma | Prostate | Prostate | Yes |
| 274 | TCGA-G2-A2EC | Bladder Urothelial Carcinoma | Bladder | Bladder | Yes |
| 275 | TCGA-2F-A9KW | Bladder Urothelial Carcinoma | Bladder | Bladder | Yes |
| 276 | TCGA-HT-8113 | Brain Lower Grade Glioma | Brain | Brain | Yes |
| 277 | TCGA-G8-6909 | Lymphoid Neoplasm Diffuse Large B-cell Lymphoma | Esophagus | Esophagus | Yes |
| 278 | TCGA-ZF-AA5P | Bladder Urothelial Carcinoma | Bladder | Bladder | Yes |
| 279 | TCGA-C8-A27A | Breast Invasive Carcinoma | Breast | Breast | Yes |
| 280 | TCGA-BH-A0B6 | Breast Invasive Carcinoma | Breast | Breast | Yes |
| 281 | TCGA-VQ-A91X | Stomach Adenocarcinoma | Testis | Testis | Yes |
| 282 | TCGA-CV-6940 | Head and Neck Squamous Cell Carcinoma | Head and Neck | Head and Neck | Yes |
| 283 | TCGA-2G-AALO |  | Thyroid | Thyroid | Yes |
| 284 | TCGA-F5-6571 | Rectum Adenocarcinoma | Soft Tissue | Soft Tissue | Yes |
| 285 | TCGA-SH-A9CT | Mesothelioma | Pancreas | Pancreas | Yes |
| 286 | TCGA-DX-AATS | Sarcoma | Skin | Skin | Yes |
| 287 | TCGA-CN-6024 | Head and Neck Squamous Cell Carcinoma | Head and Neck | Head and Neck | Yes |
| 288 | TCGA-HC-A6AO | Prostate Adenocarcinoma | Soft Tissue | Soft Tissue | Yes |
| 289 | TCGA-D8-A1Y2 | Breast Invasive Carcinoma | Breast | Breast | Yes |
| 290 | TCGA-EJ-5516 | Prostate Adenocarcinoma | Prostate | Prostate | Yes |
| 291 | TCGA-DH-5141 | Brain Lower Grade Glioma | Brain | Brain | Yes |
| 292 | TCGA-50-5946 | Lung Adenocarcinoma | Lung | Lung | Yes |
| 293 | TCGA-EL-A3T2 | Thyroid Carcinoma | Thyroid | Thyroid | Yes |
| 294 | TCGA-AA-3511 | Colon Adenocarcinoma | Esophagus | Stomach | No |
| 295 | TCGA-49-6761 | Lung Adenocarcinoma | Lung | Lung | Yes |
| 296 | TCGA-CS-4938 | Brain Lower Grade Glioma | Brain | Brain | Yes |
| 297 | TCGA-44-3918 | Lung Adenocarcinoma | Lung | Lung | Yes |
| 298 | TCGA-B0-4811 | Kidney Renal Clear Cell Carcinoma | Kidney | Kidney | Yes |
| 299 | TCGA-DX-A3M1 | Sarcoma | Stomach | Stomach | Yes |
| 300 | TCGA-44-2666 | Lung Adenocarcinoma | Lung | Lung | Yes |
| 301 | TCGA-WB-A816 | Pheochromocytoma and Paraganglioma | Prostate | Prostate | Yes |
| 302 | TCGA-94-A5I6 | Lung Squamous Cell Carcinoma | Lung | Lung | Yes |
| 303 | TCGA-XK-AAJT | Prostate Adenocarcinoma | Colorectal | Colorectal | Yes |
| 304 | TCGA-HU-8602 | Stomach Adenocarcinoma | Stomach | Stomach | Yes |
| 305 | TCGA-TM-A84F | Brain Lower Grade Glioma | Brain | Brain | Yes |
| 306 | TCGA-BP-5189 | Kidney Renal Clear Cell Carcinoma | Kidney | Kidney | Yes |
| 307 | TCGA-AD-6964 | Colon Adenocarcinoma | Colorectal | Colorectal | Yes |
| 308 | TCGA-NC-A5HJ | Lung Squamous Cell Carcinoma | Lung | Lung | Yes |
| 309 | TCGA-39-5024 | Lung Squamous Cell Carcinoma | Lung | Lung | Yes |
| 310 | TCGA-K4-A4AB | Bladder Urothelial Carcinoma | Bladder | Bladder | Yes |
| 311 | TCGA-06-5408 | Glioblastoma Multiforme | Head and Neck | Head and Neck | Yes |
| 312 | TCGA-26-6174 | Glioblastoma Multiforme | Head and Neck | Head and Neck | Yes |
| 313 | TCGA-IN-AB1V | Stomach Adenocarcinoma | Stomach | Stomach | Yes |
| 314 | TCGA-QR-A6H4 | Pheochromocytoma and Paraganglioma | Prostate | Prostate | Yes |
| 315 | TCGA-BA-6868 | Head and Neck Squamous Cell Carcinoma | Head and Neck | Head and Neck | Yes |
| 316 | TCGA-EL-A3T9 | Thyroid Carcinoma | Thyroid | Thyroid | Yes |
| 317 | TCGA-IB-A7M4 | Pancreatic Adenocarcinoma | Adrenal Gland | Adrenal Gland | Yes |
| 318 | TCGA-B2-5635 | Kidney Renal Clear Cell Carcinoma | Kidney | Kidney | Yes |
| 319 | TCGA-P4-AAVL | Kidney Renal Papillary Cell Carcinoma | Brain | Brain | Yes |
| 320 | TCGA-E1-A7YK | Brain Lower Grade Glioma | Liver | Liver | Yes |
| 321 | TCGA-TP-A8TT | Prostate Adenocarcinoma | Prostate | Prostate | Yes |
| 322 | TCGA-CK-4948 | Colon Adenocarcinoma | Colorectal | Colorectal | Yes |
| 323 | TCGA-J4-AATV | Prostate Adenocarcinoma | Prostate | Prostate | Yes |
| 324 | TCGA-CN-6013 | Head and Neck Squamous Cell Carcinoma | Head and Neck | Head and Neck | Yes |
| 325 | TCGA-DX-A23Z | Sarcoma | Skin | Skin | Yes |
| 326 | TCGA-A7-A6VX | Breast Invasive Carcinoma | Breast | Breast | Yes |
| 327 | TCGA-F2-7273 | Pancreatic Adenocarcinoma | Prostate | Prostate | Yes |
| 328 | TCGA-DD-AAW0 | Liver Hepatocellular Carcinoma | Lung | Lung | Yes |
| 329 | TCGA-HQ-A5NE | Bladder Urothelial Carcinoma | Bladder | Bladder | Yes |
| 330 | TCGA-AA-3495 | Colon Adenocarcinoma | Colorectal | Colorectal | Yes |
| 331 | TCGA-OL-A5D8 | Breast Invasive Carcinoma | Breast | Breast | Yes |
| 332 | TCGA-VV-A86M | Brain Lower Grade Glioma | Brain | Brain | Yes |
| 333 | TCGA-BH-A1EV | Breast Invasive Carcinoma | Breast | Breast | Yes |
| 334 | TCGA-CQ-5325 | Head and Neck Squamous Cell Carcinoma | Head and Neck | Head and Neck | Yes |
| 335 | TCGA-BT-A0YX | Bladder Urothelial Carcinoma | Bladder | Bladder | Yes |
| 336 | TCGA-OL-A5RV | Breast Invasive Carcinoma | Breast | Breast | Yes |
| 337 | TCGA-86-8358 | Lung Adenocarcinoma | Lung | Lung | Yes |
| 338 | TCGA-C8-A1HI | Breast Invasive Carcinoma | Breast | Breast | Yes |
| 339 | TCGA-69-8253 | Lung Adenocarcinoma | Lung | Lung | Yes |
| 340 | TCGA-CU-A0YR | Bladder Urothelial Carcinoma | Bladder | Bladder | Yes |
| 341 | TCGA-GC-A3RC | Bladder Urothelial Carcinoma | Bladder | Bladder | Yes |
| 342 | TCGA-DJ-A1QD | Thyroid Carcinoma | Thymus | Bladder | No |
| 343 | TCGA-RX-A8JQ | Pheochromocytoma and Paraganglioma | Prostate | Prostate | Yes |
| 344 | TCGA-WJ-A86L | Liver Hepatocellular Carcinoma | Liver | Liver | Yes |
| 345 | TCGA-BH-A0W5 | Breast Invasive Carcinoma | Breast | Breast | Yes |
| 346 | TCGA-AC-A3TM | Breast Invasive Carcinoma | Breast | Breast | Yes |
| 347 | TCGA-HD-8635 | Head and Neck Squamous Cell Carcinoma | Head and Neck | Head and Neck | Yes |
| 348 | TCGA-A4-7583 | Kidney Renal Papillary Cell Carcinoma | Kidney | Kidney | Yes |
| 349 | TCGA-85-A50Z | Lung Squamous Cell Carcinoma | Lung | Lung | Yes |
| 350 | TCGA-ZF-AA4T | Bladder Urothelial Carcinoma | Bladder | Bladder | Yes |
| 351 | TCGA-55-6978 | Lung Adenocarcinoma | Lung | Lung | Yes |
| 352 | TCGA-XJ-A9DI | Prostate Adenocarcinoma | Prostate | Prostate | Yes |
| 353 | TCGA-A7-A13F | Breast Invasive Carcinoma | Breast | Breast | Yes |
| 354 | TCGA-2Z-A9J8 | Kidney Renal Papillary Cell Carcinoma | Brain | Brain | Yes |
| 355 | TCGA-E7-A8O7 | Bladder Urothelial Carcinoma | Bladder | Bladder | Yes |
| 356 | TCGA-FD-A5BV | Bladder Urothelial Carcinoma | Bladder | Bladder | Yes |
| 357 | TCGA-A8-A0AD | Breast Invasive Carcinoma | Breast | Breast | Yes |
| 358 | TCGA-SR-A6MT | Pheochromocytoma and Paraganglioma | Prostate | Prostate | Yes |
| 359 | TCGA-BR-8285 | Stomach Adenocarcinoma | Stomach | Stomach | Yes |
| 360 | TCGA-ZD-A8I3 | Cholangiocarcinoma | Colorectal | Colorectal | Yes |
| 361 | TCGA-FG-A87Q | Brain Lower Grade Glioma | Brain | Brain | Yes |
| 362 | TCGA-AD-6895 | Colon Adenocarcinoma | Esophagus | Esophagus | Yes |
| 363 | TCGA-CN-4740 | Head and Neck Squamous Cell Carcinoma | Head and Neck | Head and Neck | Yes |
| 364 | TCGA-56-8624 | Lung Squamous Cell Carcinoma | Lung | Lung | Yes |
| 365 | TCGA-2Z-A9JL | Kidney Renal Papillary Cell Carcinoma | Brain | Brain | Yes |
| 366 | TCGA-CW-5581 | Kidney Renal Clear Cell Carcinoma | Kidney | Kidney | Yes |
| 367 | TCGA-CC-5259 | Liver Hepatocellular Carcinoma | Liver | Liver | Yes |
| 368 | TCGA-OR-A5LD | Adrenocortical Carcinoma | Adrenal Gland | Adrenal Gland | Yes |
| 369 | TCGA-KQ-A41Q | Bladder Urothelial Carcinoma | Bladder | Bladder | Yes |
| 370 | TCGA-ZF-AA58 | Bladder Urothelial Carcinoma | Bladder | Bladder | Yes |
| 371 | TCGA-EW-A1P7 | Breast Invasive Carcinoma | Breast | Breast | Yes |
| 372 | TCGA-L5-A4OG | Esophageal Carcinoma | Esophagus | Stomach | No |
| 373 | TCGA-BH-A203 | Breast Invasive Carcinoma | Breast | Breast | Yes |
| 374 | TCGA-VQ-AA6G | Stomach Adenocarcinoma | Testis | Testis | Yes |
| 375 | TCGA-DD-A1EG | Liver Hepatocellular Carcinoma | Lung | Lung | Yes |
| 376 | TCGA-B0-5706 | Kidney Renal Clear Cell Carcinoma | Kidney | Kidney | Yes |
| 377 | TCGA-C9-A480 | Head and Neck Squamous Cell Carcinoma | Head and Neck | Head and Neck | Yes |
| 378 | TCGA-V1-A8WN | Prostate Adenocarcinoma | Soft Tissue | Soft Tissue | Yes |
| 379 | TCGA-RY-A83Z | Brain Lower Grade Glioma | Liver | Liver | Yes |
| 380 | TCGA-L5-A8NE | Esophageal Carcinoma | Esophagus | Esophagus | Yes |
| 381 | TCGA-G7-6797 | Kidney Renal Papillary Cell Carcinoma | Brain | Brain | Yes |
| 382 | TCGA-39-5040 | Lung Squamous Cell Carcinoma | Lung | Lung | Yes |
| 383 | TCGA-55-8094 | Lung Adenocarcinoma | Lung | Lung | Yes |
| 384 | TCGA-2Y-A9HA | Liver Hepatocellular Carcinoma | Liver | Liver | Yes |
| 385 | TCGA-BA-7269 | Head and Neck Squamous Cell Carcinoma | Head and Neck | Head and Neck | Yes |
| 386 | TCGA-76-6662 | Glioblastoma Multiforme | Head and Neck | Head and Neck | Yes |
| 387 | TCGA-06-0221 | Glioblastoma Multiforme | Brain | Brain | Yes |
| 388 | TCGA-FY-A40M | Thyroid Carcinoma | Thyroid | Thyroid | Yes |
| 389 | TCGA-2G-AAHA | Testicular Germ Cell Tumors | Thyroid | Thyroid | Yes |
| 390 | TCGA-A4-7287 | Kidney Renal Papillary Cell Carcinoma | Kidney | Kidney | Yes |
| 391 | TCGA-TQ-A7RH | Brain Lower Grade Glioma | Liver | Liver | Yes |
| 392 | TCGA-D7-A748 | Stomach Adenocarcinoma | Testis | Testis | Yes |
| 393 | TCGA-06-5413 | Glioblastoma Multiforme | Brain | Brain | Yes |
| 394 | TCGA-EB-A430 | Skin Cutaneous Melanoma | Stomach | Stomach | Yes |
| 395 | TCGA-LN-A4A2 | Esophageal Carcinoma | Brain | Brain | Yes |
| 396 | TCGA-E2-A573 | Breast Invasive Carcinoma | Breast | Breast | Yes |
| 397 | TCGA-CK-5915 | Colon Adenocarcinoma | Colorectal | Stomach | No |
| 398 | TCGA-G9-7525 | Prostate Adenocarcinoma | Prostate | Prostate | Yes |
| 399 | TCGA-RD-A7C1 | Stomach Adenocarcinoma | Testis | Testis | Yes |
| 400 | TCGA-SW-A7EB | Stomach Adenocarcinoma | Stomach | Stomach | Yes |
| 401 | TCGA-CR-7369 | Head and Neck Squamous Cell Carcinoma | Head and Neck | Head and Neck | Yes |
| 402 | TCGA-FC-A4JI | Prostate Adenocarcinoma | Prostate | Prostate | Yes |
| 403 | TCGA-BP-5174 | Kidney Renal Clear Cell Carcinoma | Kidney | Kidney | Yes |
| 404 | TCGA-VQ-A8PH | Stomach Adenocarcinoma | Stomach | Stomach | Yes |
| 405 | TCGA-SC-A6LP | Mesothelioma | Pancreas | Pancreas | Yes |
| 406 | TCGA-G5-6641 | Rectum Adenocarcinoma | Soft Tissue | Soft Tissue | Yes |
| 407 | TCGA-75-5122 | Lung Adenocarcinoma | Lung | Lung | Yes |
| 408 | TCGA-HC-7821 | Prostate Adenocarcinoma | Colorectal | Colorectal | Yes |
| 409 | TCGA-ED-A5KG | Liver Hepatocellular Carcinoma | Lung | Lung | Yes |
| 410 | TCGA-XF-A9T5 | Bladder Urothelial Carcinoma | Bladder | Esophagus | No |
| 411 | TCGA-YL-A8SF | Prostate Adenocarcinoma | Prostate | Prostate | Yes |
| 412 | TCGA-J8-A3YH | Thyroid Carcinoma | Thymus | Thymus | Yes |
| 413 | TCGA-DX-AB2T | Sarcoma | Skin | Skin | Yes |
| 414 | TCGA-XF-A9SJ | Bladder Urothelial Carcinoma | Bladder | Bladder | Yes |
| 415 | TCGA-43-6773 | Lung Squamous Cell Carcinoma | Pancreas | Pancreas | Yes |
| 416 | TCGA-KQ-A41N | Bladder Urothelial Carcinoma | Bladder | Bladder | Yes |
| 417 | TCGA-XF-AAN7 | Bladder Urothelial Carcinoma | Bladder | Bladder | Yes |
| 418 | TCGA-CM-4747 | Colon Adenocarcinoma | Colorectal | Colorectal | Yes |
| 419 | TCGA-A2-A3XV | Breast Invasive Carcinoma | Breast | Breast | Yes |
| 420 | TCGA-ET-A3DR | Thyroid Carcinoma | Thyroid | Thyroid | Yes |
| 421 | TCGA-CZ-5460 | Kidney Renal Clear Cell Carcinoma | Kidney | Kidney | Yes |
| 422 | TCGA-P5-A72U | Brain Lower Grade Glioma | Brain | Brain | Yes |
| 423 | TCGA-R6-A6XQ | Esophageal Carcinoma | Brain | Brain | Yes |
| 424 | TCGA-OL-A5RW | Breast Invasive Carcinoma | Breast | Breast | Yes |
| 425 | TCGA-BH-A0C0 | Breast Invasive Carcinoma | Breast | Breast | Yes |
| 426 | TCGA-MZ-A5BI | Head and Neck Squamous Cell Carcinoma | Kidney | Kidney | Yes |
| 427 | TCGA-HC-8259 | Prostate Adenocarcinoma | Prostate | Prostate | Yes |
| 428 | TCGA-KK-A6E8 | Prostate Adenocarcinoma | Soft Tissue | Soft Tissue | Yes |
| 429 | TCGA-LN-A9FO | Esophageal Carcinoma | Esophagus | Stomach | No |
| 430 | TCGA-V1-A8MK | Prostate Adenocarcinoma | Soft Tissue | Soft Tissue | Yes |
| 431 | TCGA-OL-A66P | Breast Invasive Carcinoma | Breast | Breast | Yes |
| 432 | TCGA-06-5412 | Glioblastoma Multiforme | Head and Neck | Head and Neck | Yes |
| 433 | TCGA-HT-8015 | Brain Lower Grade Glioma | Brain | Brain | Yes |
| 434 | TCGA-IW-A3M5 | Sarcoma | Skin | Skin | Yes |
| 435 | TCGA-CV-7104 | Head and Neck Squamous Cell Carcinoma | Kidney | Kidney | Yes |
| 436 | TCGA-A4-7584 | Kidney Renal Papillary Cell Carcinoma | Kidney | Kidney | Yes |
| 437 | TCGA-DU-7290 | Brain Lower Grade Glioma | Brain | Brain | Yes |
| 438 | TCGA-VP-A87J | Prostate Adenocarcinoma | Soft Tissue | Soft Tissue | Yes |
| 439 | TCGA-D8-A27F | Breast Invasive Carcinoma | Breast | Breast | Yes |
| 440 | TCGA-AQ-A04L | Breast Invasive Carcinoma | Breast | Breast | Yes |
| 441 | TCGA-G4-6295 | Colon Adenocarcinoma | Colorectal | Colorectal | Yes |
| 442 | TCGA-49-AAR4 | Lung Adenocarcinoma | Lung | Lung | Yes |
| 443 | TCGA-ED-A627 | Liver Hepatocellular Carcinoma | Liver | Liver | Yes |
| 444 | TCGA-CV-7430 | Head and Neck Squamous Cell Carcinoma | Head and Neck | Head and Neck | Yes |
| 445 | TCGA-SC-A6LR | Mesothelioma | Pancreas | Pancreas | Yes |
| 446 | TCGA-AO-A0JF | Breast Invasive Carcinoma | Breast | Breast | Yes |
| 447 | TCGA-BR-8081 | Stomach Adenocarcinoma | Testis | Testis | Yes |
| 448 | TCGA-39-5030 | Lung Squamous Cell Carcinoma | Lung | Lung | Yes |
| 449 | TCGA-DU-8161 | Brain Lower Grade Glioma | Liver | Liver | Yes |
| 450 | TCGA-43-8118 | Lung Squamous Cell Carcinoma | Lung | Lung | Yes |
| 451 | TCGA-CM-6165 | Colon Adenocarcinoma | Colorectal | Colorectal | Yes |
| 452 | TCGA-DC-6157 | Rectum Adenocarcinoma | Soft Tissue | Soft Tissue | Yes |
| 453 | TCGA-HZ-A4BH | Pancreatic Adenocarcinoma | Adrenal Gland | Adrenal Gland | Yes |
| 454 | TCGA-LL-A8F5 | Breast Invasive Carcinoma | Breast | Breast | Yes |
| 455 | TCGA-BR-6565 | Stomach Adenocarcinoma | Stomach | Stomach | Yes |
| 456 | TCGA-CD-8524 | Stomach Adenocarcinoma | Thyroid | Thyroid | Yes |
| 457 | TCGA-CN-6988 | Head and Neck Squamous Cell Carcinoma | Head and Neck | Head and Neck | Yes |
| 458 | TCGA-IE-A4EH | Sarcoma | Stomach | Stomach | Yes |
| 459 | TCGA-TM-A84O | Brain Lower Grade Glioma | Brain | Brain | Yes |
| 460 | TCGA-KL-8345 | Kidney Chromophobe | Kidney | Kidney | Yes |
| 461 | TCGA-DU-5871 | Brain Lower Grade Glioma | Brain | Brain | Yes |
| 462 | TCGA-IB-A5SS | Pancreatic Adenocarcinoma | Adrenal Gland | Adrenal Gland | Yes |
| 463 | TCGA-DJ-A3UV | Thyroid Carcinoma | Thymus | Thymus | Yes |
| 464 | TCGA-KK-A6DY | Prostate Adenocarcinoma | Prostate | Prostate | Yes |
| 465 | TCGA-26-5135 | Glioblastoma Multiforme | Head and Neck | Head and Neck | Yes |
| 466 | TCGA-CH-5751 | Prostate Adenocarcinoma | Colorectal | Colorectal | Yes |
| 467 | TCGA-YS-AA4M | Mesothelioma | Pancreas | Pancreas | Yes |
| 468 | TCGA-BA-5152 | Head and Neck Squamous Cell Carcinoma | Head and Neck | Head and Neck | Yes |
| 469 | TCGA-L5-A8NG | Esophageal Carcinoma | Brain | Brain | Yes |
| 470 | TCGA-KC-A7F6 | Prostate Adenocarcinoma | Soft Tissue | Soft Tissue | Yes |
| 471 | TCGA-B0-5712 | Kidney Renal Clear Cell Carcinoma | Kidney | Kidney | Yes |
| 472 | TCGA-TS-A8AV | Mesothelioma | Pancreas | Pancreas | Yes |
| 473 | TCGA-CV-7245 | Head and Neck Squamous Cell Carcinoma | Head and Neck | Head and Neck | Yes |
| 474 | TCGA-DD-A4NB | Liver Hepatocellular Carcinoma | Liver | Liver | Yes |
| 475 | TCGA-AM-5820 | Colon Adenocarcinoma | Colorectal | Colorectal | Yes |
| 476 | TCGA-HT-7470 | Brain Lower Grade Glioma | Brain | Brain | Yes |
| 477 | TCGA-NC-A5HL | Lung Squamous Cell Carcinoma | Lung | Lung | Yes |
| 478 | TCGA-S9-A6WG | Brain Lower Grade Glioma | Brain | Brain | Yes |
| 479 | TCGA-EL-A3GZ | Thyroid Carcinoma | Thyroid | Thyroid | Yes |
| 480 | TCGA-LI-A67I | Sarcoma | Soft Tissue | Soft Tissue | Yes |
| 481 | TCGA-MQ-A6BN | Mesothelioma | Pancreas | Pancreas | Yes |
| 482 | TCGA-G6-A8L8 | Kidney Renal Clear Cell Carcinoma | Kidney | Kidney | Yes |
| 483 | TCGA-BA-A4IH | Head and Neck Squamous Cell Carcinoma | Head and Neck | Head and Neck | Yes |
| 484 | TCGA-62-A46O | Lung Adenocarcinoma | Lung | Lung | Yes |
| 485 | TCGA-EJ-AB20 | Prostate Adenocarcinoma | Soft Tissue | Soft Tissue | Yes |
| 486 | TCGA-3B-A9HI | Sarcoma | Soft Tissue | Soft Tissue | Yes |
| 487 | TCGA-PR-A5PF | Pheochromocytoma and Paraganglioma | Prostate | Prostate | Yes |
| 488 | TCGA-CQ-A4CE | Head and Neck Squamous Cell Carcinoma | Head and Neck | Esophagus | No |
| 489 | TCGA-AZ-4682 | Colon Adenocarcinoma | Colorectal | Colorectal | Yes |
| 490 | TCGA-DK-A3IL | Bladder Urothelial Carcinoma | Bladder | Bladder | Yes |
| 491 | TCGA-MO-A47P | Sarcoma | Soft Tissue | Soft Tissue | Yes |
| 492 | TCGA-55-7994 | Lung Adenocarcinoma | Lung | Lung | Yes |
| 493 | TCGA-55-7727 | Lung Adenocarcinoma | Lung | Lung | Yes |
| 494 | TCGA-DD-AADF | Liver Hepatocellular Carcinoma | Liver | Liver | Yes |
| 495 | TCGA-4A-A93Y | Kidney Renal Papillary Cell Carcinoma | Kidney | Kidney | Yes |
| 496 | TCGA-RW-A681 | Pheochromocytoma and Paraganglioma | Prostate | Prostate | Yes |
| 497 | TCGA-MP-A4T8 | Lung Adenocarcinoma | Lung | Lung | Yes |
| 498 | TCGA-EJ-5531 | Prostate Adenocarcinoma | Colorectal | Colorectal | Yes |
| 499 | TCGA-AO-A12E | Breast Invasive Carcinoma | Breast | Breast | Yes |
| 500 | TCGA-BH-A1FG | Breast Invasive Carcinoma | Breast | Breast | Yes |
| 501 | TCGA-D6-6826 | Head and Neck Squamous Cell Carcinoma | Head and Neck | Head and Neck | Yes |
| 502 | TCGA-MW-A4EC | Kidney Renal Clear Cell Carcinoma | Kidney | Kidney | Yes |
| 503 | TCGA-12-5299 | Glioblastoma Multiforme | Head and Neck | Head and Neck | Yes |
| 504 | TCGA-2H-A9GM | Esophageal Carcinoma | Esophagus | Bladder | No |
| 505 | TCGA-91-8496 | Lung Adenocarcinoma | Lung | Lung | Yes |
| 506 | TCGA-RD-A7BT | Stomach Adenocarcinoma | Stomach | Stomach | Yes |
| 507 | TCGA-CR-6471 | Head and Neck Squamous Cell Carcinoma | Kidney | Kidney | Yes |
| 508 | TCGA-TS-A7P7 | Mesothelioma | Pancreas | Pancreas | Yes |
| 509 | TCGA-D6-8569 | Head and Neck Squamous Cell Carcinoma | Head and Neck | Esophagus | No |
| 510 | TCGA-HC-A6AS | Prostate Adenocarcinoma | Prostate | Prostate | Yes |
| 511 | TCGA-FZ-5922 | Pancreatic Adenocarcinoma | Adrenal Gland | Adrenal Gland | Yes |
| 512 | TCGA-TM-A7CA | Brain Lower Grade Glioma | Brain | Brain | Yes |
| 513 | TCGA-GI-A2C9 | Breast Invasive Carcinoma | Breast | Breast | Yes |
| 514 | TCGA-EJ-A8FN | Prostate Adenocarcinoma | Prostate | Prostate | Yes |
| 515 | TCGA-ZH-A8Y5 | Cholangiocarcinoma | Colorectal | Colorectal | Yes |
| 516 | TCGA-XQ-A8TB | Prostate Adenocarcinoma | Soft Tissue | Soft Tissue | Yes |
| 517 | TCGA-55-6984 | Lung Adenocarcinoma | Lung | Lung | Yes |
| 518 | TCGA-D5-6930 | Colon Adenocarcinoma | Colorectal | Colorectal | Yes |
| 519 | TCGA-E7-A4IJ | Bladder Urothelial Carcinoma | Bladder | Bladder | Yes |
| 520 | TCGA-DU-A5TS | Brain Lower Grade Glioma | Brain | Brain | Yes |
| 521 | TCGA-SP-A6QI | Pheochromocytoma and Paraganglioma | Prostate | Prostate | Yes |
| 522 | TCGA-E2-A1LG | Breast Invasive Carcinoma | Breast | Breast | Yes |
| 523 | TCGA-B0-5696 | Kidney Renal Clear Cell Carcinoma | Kidney | Kidney | Yes |
| 524 | TCGA-E2-A105 | Breast Invasive Carcinoma | Breast | Breast | Yes |
| 525 | TCGA-S9-A6U5 | Brain Lower Grade Glioma | Brain | Brain | Yes |
| 526 | TCGA-J8-A3YD | Thyroid Carcinoma | Thyroid | Thyroid | Yes |
| 527 | TCGA-VQ-A8PP | Stomach Adenocarcinoma | Stomach | Stomach | Yes |
| 528 | TCGA-A2-A1FV | Breast Invasive Carcinoma | Breast | Breast | Yes |
| 529 | TCGA-VQ-A91Z | Stomach Adenocarcinoma | Stomach | Stomach | Yes |
| 530 | TCGA-F7-A61W | Head and Neck Squamous Cell Carcinoma | Head and Neck | Head and Neck | Yes |
| 531 | TCGA-FD-A3SM | Bladder Urothelial Carcinoma | Bladder | Bladder | Yes |
| 532 | TCGA-NJ-A55R | Lung Adenocarcinoma | Lung | Lung | Yes |
| 533 | TCGA-CC-A123 | Liver Hepatocellular Carcinoma | Liver | Liver | Yes |
| 534 | TCGA-CM-4750 | Colon Adenocarcinoma | Colorectal | Colorectal | Yes |
| 535 | TCGA-EM-A3FM | Thyroid Carcinoma | Thyroid | Thyroid | Yes |
| 536 | TCGA-BA-5555 | Head and Neck Squamous Cell Carcinoma | Head and Neck | Head and Neck | Yes |
| 537 | TCGA-RC-A7SH | Liver Hepatocellular Carcinoma | Liver | Liver | Yes |
| 538 | TCGA-EW-A1PE | Breast Invasive Carcinoma | Breast | Breast | Yes |
| 539 | TCGA-CR-6493 | Head and Neck Squamous Cell Carcinoma | Kidney | Kidney | Yes |
| 540 | TCGA-D7-6524 | Stomach Adenocarcinoma | Testis | Testis | Yes |
| 541 | TCGA-2A-A8VT | Prostate Adenocarcinoma | Prostate | Prostate | Yes |
| 542 | TCGA-P5-A5F4 | Brain Lower Grade Glioma | Brain | Brain | Yes |
| 543 | TCGA-YL-A9WK | Prostate Adenocarcinoma | Colorectal | Colorectal | Yes |
| 544 | TCGA-F7-A623 | Head and Neck Squamous Cell Carcinoma | Head and Neck | Head and Neck | Yes |
| 545 | TCGA-BP-5198 | Kidney Renal Clear Cell Carcinoma | Kidney | Kidney | Yes |
| 546 | TCGA-UY-A78M | Bladder Urothelial Carcinoma | Bladder | Bladder | Yes |
| 547 | TCGA-LK-A4NW | Mesothelioma | Pancreas | Pancreas | Yes |
| 548 | TCGA-EB-A44O | Skin Cutaneous Melanoma | Stomach | Stomach | Yes |
| 549 | TCGA-EW-A1IX | Breast Invasive Carcinoma | Breast | Breast | Yes |
| 550 | TCGA-EB-A550 | Skin Cutaneous Melanoma | Stomach | Stomach | Yes |
| 551 | TCGA-VQ-A8PT | Stomach Adenocarcinoma | Thyroid | Thyroid | Yes |
| 552 | TCGA-H7-8502 | Head and Neck Squamous Cell Carcinoma | Head and Neck | Head and Neck | Yes |
| 553 | TCGA-CG-5730 | Stomach Adenocarcinoma | Stomach | Stomach | Yes |
| 554 | TCGA-VQ-AA68 | Stomach Adenocarcinoma | Testis | Testis | Yes |
| 555 | TCGA-CD-5803 | Stomach Adenocarcinoma | Stomach | Esophagus | No |
| 556 | TCGA-IB-AAUO | Pancreatic Adenocarcinoma | Adrenal Gland | Adrenal Gland | Yes |
| 557 | TCGA-AC-A6IV | Breast Invasive Carcinoma | Breast | Breast | Yes |
| 558 | TCGA-3A-A9IR | Pancreatic Adenocarcinoma | Adrenal Gland | Pancreas | No |
| 559 | TCGA-G5-6235 | Rectum Adenocarcinoma | Soft Tissue | Soft Tissue | Yes |
| 560 | TCGA-DB-A75K | Brain Lower Grade Glioma | Brain | Brain | Yes |
| 561 | TCGA-EL-A3MZ | Thyroid Carcinoma | Thyroid | Thyroid | Yes |
| 562 | TCGA-IB-7645 | Pancreatic Adenocarcinoma | Adrenal Gland | Adrenal Gland | Yes |
| 563 | TCGA-2G-AAGV | Testicular Germ Cell Tumors | Thyroid | Thyroid | Yes |
| 564 | TCGA-WB-A81M | Pheochromocytoma and Paraganglioma | Prostate | Prostate | Yes |
| 565 | TCGA-RW-A68G | Pheochromocytoma and Paraganglioma | Prostate | Prostate | Yes |
| 566 | TCGA-P3-A6T7 | Head and Neck Squamous Cell Carcinoma | Head and Neck | Head and Neck | Yes |
| 567 | TCGA-DX-AB2J | Sarcoma | Skin | Skin | Yes |
| 568 | TCGA-DD-AAEK | Liver Hepatocellular Carcinoma | Lung | Lung | Yes |
| 569 | TCGA-A2-A3XW | Breast Invasive Carcinoma | Breast | Breast | Yes |
| 570 | TCGA-WK-A8XY | Sarcoma | Skin | Skin | Yes |
| 571 | TCGA-J4-A6G3 | Prostate Adenocarcinoma | Colorectal | Colorectal | Yes |
| 572 | TCGA-DX-A8BG | Sarcoma | Soft Tissue | Soft Tissue | Yes |
| 573 | TCGA-DJ-A1QF | Thyroid Carcinoma | Thyroid | Thyroid | Yes |
| 574 | TCGA-IB-7885 | Pancreatic Adenocarcinoma | Adrenal Gland | Adrenal Gland | Yes |
| 575 | TCGA-DV-5573 | Kidney Renal Clear Cell Carcinoma | Kidney | Kidney | Yes |
| 576 | TCGA-E1-A7Z6 | Brain Lower Grade Glioma | Brain | Brain | Yes |
| 577 | TCGA-DU-7302 | Brain Lower Grade Glioma | Brain | Brain | Yes |
| 578 | TCGA-96-7544 | Lung Squamous Cell Carcinoma | Lung | Lung | Yes |
| 579 | TCGA-4A-A93X | Kidney Renal Papillary Cell Carcinoma | Brain | Brain | Yes |
| 580 | TCGA-G9-6351 | Prostate Adenocarcinoma | Prostate | Prostate | Yes |
| 581 | TCGA-OR-A5KO | Adrenocortical Carcinoma | Adrenal Gland | Adrenal Gland | Yes |
| 582 | TCGA-55-6986 | Lung Adenocarcinoma | Lung | Lung | Yes |
| 583 | TCGA-B1-A655 | Kidney Renal Papillary Cell Carcinoma | Kidney | Kidney | Yes |
| 584 | TCGA-DM-A28A | Colon Adenocarcinoma | Esophagus | Stomach | No |
| 585 | TCGA-91-8497 | Lung Adenocarcinoma | Lung | Lung | Yes |
| 586 | TCGA-VN-A88P | Prostate Adenocarcinoma | Prostate | Prostate | Yes |
| 587 | TCGA-IB-7888 | Pancreatic Adenocarcinoma | Adrenal Gland | Adrenal Gland | Yes |
| 588 | TCGA-KT-A7W1 | Brain Lower Grade Glioma | Liver | Liver | Yes |
| 589 | TCGA-EL-A3CL | Thyroid Carcinoma | Thyroid | Thyroid | Yes |
| 590 | TCGA-HC-A6AL | Prostate Adenocarcinoma | Colorectal | Colorectal | Yes |
| 591 | TCGA-IQ-7631 | Head and Neck Squamous Cell Carcinoma | Kidney | Kidney | Yes |
| 592 | TCGA-EJ-8470 | Prostate Adenocarcinoma | Soft Tissue | Soft Tissue | Yes |
| 593 | TCGA-IF-A4AJ | Sarcoma | Skin | Skin | Yes |
| 594 | TCGA-S9-A6WN | Brain Lower Grade Glioma | Brain | Brain | Yes |
| 595 | TCGA-78-7147 | Lung Adenocarcinoma | Lung | Lung | Yes |
| 596 | TCGA-58-8387 | Lung Squamous Cell Carcinoma | Pancreas | Pancreas | Yes |
| 597 | TCGA-DD-AAE1 | Liver Hepatocellular Carcinoma | Liver | Liver | Yes |
| 598 | TCGA-CK-4947 | Colon Adenocarcinoma | Esophagus | Esophagus | Yes |
| 599 | TCGA-55-7816 | Lung Adenocarcinoma | Lung | Lung | Yes |
| 600 | TCGA-AG-A01W | Rectum Adenocarcinoma | Soft Tissue | Soft Tissue | Yes |
| 601 | TCGA-K1-A6RT | Sarcoma | Soft Tissue | Soft Tissue | Yes |
| 602 | TCGA-EI-6882 | Rectum Adenocarcinoma | Soft Tissue | Soft Tissue | Yes |
| 603 | TCGA-33-4586 | Lung Squamous Cell Carcinoma | Pancreas | Pancreas | Yes |
| 604 | TCGA-HZ-8317 | Pancreatic Adenocarcinoma | Adrenal Gland | Adrenal Gland | Yes |
| 605 | TCGA-HU-A4H2 | Stomach Adenocarcinoma | Stomach | Stomach | Yes |
| 606 | TCGA-OR-A5JF | Adrenocortical Carcinoma | Adrenal Gland | Adrenal Gland | Yes |
| 607 | TCGA-67-4679 | Lung Adenocarcinoma | Lung | Lung | Yes |
| 608 | TCGA-G8-6324 | Lymphoid Neoplasm Diffuse Large B-cell Lymphoma | Esophagus | Esophagus | Yes |
| 609 | TCGA-CG-5717 | Stomach Adenocarcinoma | Testis | Testis | Yes |
| 610 | TCGA-5P-A9K4 | Kidney Renal Papillary Cell Carcinoma | Kidney | Kidney | Yes |
| 611 | TCGA-QR-A70U | Pheochromocytoma and Paraganglioma | Prostate | Prostate | Yes |
| 612 | TCGA-AR-A0TU | Breast Invasive Carcinoma | Breast | Breast | Yes |
| 613 | TCGA-UB-A7MB | Liver Hepatocellular Carcinoma | Lung | Lung | Yes |
| 614 | TCGA-43-7656 | Lung Squamous Cell Carcinoma | Lung | Lung | Yes |
| 615 | TCGA-EJ-7312 | Prostate Adenocarcinoma | Prostate | Prostate | Yes |
| 616 | TCGA-CI-6621 | Rectum Adenocarcinoma | Soft Tissue | Soft Tissue | Yes |
| 617 | TCGA-BR-8058 | Stomach Adenocarcinoma | Stomach | Stomach | Yes |
| 618 | TCGA-CZ-4859 | Kidney Renal Clear Cell Carcinoma | Kidney | Kidney | Yes |
| 619 | TCGA-FG-A70Y | Brain Lower Grade Glioma | Brain | Brain | Yes |
| 620 | TCGA-L9-A443 | Lung Adenocarcinoma | Lung | Lung | Yes |
| 621 | TCGA-BA-A6DA | Head and Neck Squamous Cell Carcinoma | Kidney | Kidney | Yes |
| 622 | TCGA-CJ-5680 | Kidney Renal Clear Cell Carcinoma | Kidney | Kidney | Yes |
| 623 | TCGA-DJ-A3UR | Thyroid Carcinoma | Thymus | Thymus | Yes |
| 624 | TCGA-QU-A6IO | Prostate Adenocarcinoma | Soft Tissue | Soft Tissue | Yes |
| 625 | TCGA-HT-7687 | Brain Lower Grade Glioma | Brain | Brain | Yes |
| 626 | TCGA-AO-A03N | Breast Invasive Carcinoma | Breast | Breast | Yes |
| 627 | TCGA-OR-A5J3 | Adrenocortical Carcinoma | Adrenal Gland | Adrenal Gland | Yes |
| 628 | TCGA-AK-3458 | Kidney Renal Clear Cell Carcinoma | Kidney | Kidney | Yes |
| 629 | TCGA-D7-A6F0 | Stomach Adenocarcinoma | Stomach | Stomach | Yes |
| 630 | TCGA-IE-A4EK | Sarcoma | Stomach | Esophagus | No |
| 631 | TCGA-CJ-4901 | Kidney Renal Clear Cell Carcinoma | Kidney | Kidney | Yes |
| 632 | TCGA-EJ-A8FP | Prostate Adenocarcinoma | Prostate | Prostate | Yes |
| 633 | TCGA-55-8614 | Lung Adenocarcinoma | Lung | Lung | Yes |
| 634 | TCGA-S9-A6TW | Brain Lower Grade Glioma | Brain | Brain | Yes |
| 635 | TCGA-KN-8433 | Kidney Chromophobe | Kidney | Kidney | Yes |
| 636 | TCGA-68-8250 | Lung Squamous Cell Carcinoma | Lung | Lung | Yes |
| 637 | TCGA-2Z-A9JK | Kidney Renal Papillary Cell Carcinoma | Kidney | Kidney | Yes |
| 638 | TCGA-55-7284 | Lung Adenocarcinoma | Lung | Lung | Yes |
| 639 | TCGA-2G-AALN |  | Thyroid | Thyroid | Yes |
| 640 | TCGA-AR-A2LJ | Breast Invasive Carcinoma | Breast | Breast | Yes |
| 641 | TCGA-TT-A6YJ | Pheochromocytoma and Paraganglioma | Prostate | Prostate | Yes |
| 642 | TCGA-AF-A56N | Rectum Adenocarcinoma | Soft Tissue | Soft Tissue | Yes |
| 643 | TCGA-DX-A3LS | Sarcoma | Stomach | Esophagus | No |
| 644 | TCGA-J4-A67Q | Prostate Adenocarcinoma | Prostate | Prostate | Yes |
| 645 | TCGA-EM-A3FN | Thyroid Carcinoma | Thymus | Thymus | Yes |
| 646 | TCGA-NJ-A4YF | Lung Adenocarcinoma | Lung | Lung | Yes |
| 647 | TCGA-GU-A42Q | Bladder Urothelial Carcinoma | Bladder | Bladder | Yes |
| 648 | TCGA-IE-A4EI | Sarcoma | Skin | Skin | Yes |
| 649 | TCGA-77-7140 | Lung Squamous Cell Carcinoma | Lung | Lung | Yes |
| 650 | TCGA-BR-8286 | Stomach Adenocarcinoma | Stomach | Stomach | Yes |
| 651 | TCGA-CF-A47Y | Bladder Urothelial Carcinoma | Bladder | Bladder | Yes |
| 652 | TCGA-A1-A0SE | Breast Invasive Carcinoma | Breast | Breast | Yes |
| 653 | TCGA-CV-5432 | Head and Neck Squamous Cell Carcinoma | Head and Neck | Head and Neck | Yes |
| 654 | TCGA-FF-8041 | Lymphoid Neoplasm Diffuse Large B-cell Lymphoma | Esophagus | Esophagus | Yes |
| 655 | TCGA-SX-A71U | Kidney Renal Papillary Cell Carcinoma | Brain | Brain | Yes |
| 656 | TCGA-63-A5MJ | Lung Squamous Cell Carcinoma | Lung | Lung | Yes |
| 657 | TCGA-L5-A891 | Esophageal Carcinoma | Esophagus | Esophagus | Yes |
| 658 | TCGA-BF-A1PX | Skin Cutaneous Melanoma | Stomach | Stomach | Yes |
| 659 | TCGA-GM-A3XG | Breast Invasive Carcinoma | Breast | Breast | Yes |
| 660 | TCGA-DB-5279 | Brain Lower Grade Glioma | Brain | Brain | Yes |
| 661 | TCGA-DD-AAC9 | Liver Hepatocellular Carcinoma | Lung | Lung | Yes |
| 662 | TCGA-B0-5703 | Kidney Renal Clear Cell Carcinoma | Kidney | Kidney | Yes |
| 663 | TCGA-55-6968 | Lung Adenocarcinoma | Lung | Lung | Yes |
| 664 | TCGA-DM-A280 | Colon Adenocarcinoma | Colorectal | Colorectal | Yes |
| 665 | TCGA-VQ-A91N | Stomach Adenocarcinoma | Stomach | Stomach | Yes |
| 666 | TCGA-DU-7019 | Brain Lower Grade Glioma | Liver | Liver | Yes |
| 667 | TCGA-KK-A6E4 | Prostate Adenocarcinoma | Prostate | Prostate | Yes |
| 668 | TCGA-CV-A6JO | Head and Neck Squamous Cell Carcinoma | Head and Neck | Head and Neck | Yes |
| 669 | TCGA-QQ-A5VB | Sarcoma | Stomach | Stomach | Yes |
| 670 | TCGA-63-A5MG | Lung Squamous Cell Carcinoma | Lung | Lung | Yes |
| 671 | TCGA-KO-8407 | Kidney Chromophobe | Kidney | Kidney | Yes |
| 672 | TCGA-CN-4738 | Head and Neck Squamous Cell Carcinoma | Head and Neck | Esophagus | No |
| 673 | TCGA-G8-6906 | Lymphoid Neoplasm Diffuse Large B-cell Lymphoma | Esophagus | Esophagus | Yes |
| 674 | TCGA-86-8073 | Lung Adenocarcinoma | Lung | Lung | Yes |
| 675 | TCGA-AC-A8OP | Breast Invasive Carcinoma | Breast | Breast | Yes |
| 676 | TCGA-CN-6019 | Head and Neck Squamous Cell Carcinoma | Head and Neck | Head and Neck | Yes |
| 677 | TCGA-AA-3496 | Colon Adenocarcinoma | Colorectal | Colorectal | Yes |
| 678 | TCGA-S8-A6BW | Esophageal Carcinoma | Esophagus | Esophagus | Yes |
| 679 | TCGA-CV-6948 | Head and Neck Squamous Cell Carcinoma | Head and Neck | Head and Neck | Yes |
| 680 | TCGA-76-4926 | Glioblastoma Multiforme | Head and Neck | Head and Neck | Yes |
| 681 | TCGA-EM-A1YD | Thyroid Carcinoma | Thyroid | Thyroid | Yes |
| 682 | TCGA-RB-A7B8 | Pancreatic Adenocarcinoma | Adrenal Gland | Adrenal Gland | Yes |
| 683 | TCGA-XF-A8HG | Bladder Urothelial Carcinoma | Bladder | Bladder | Yes |
| 684 | TCGA-ZF-A9RG | Bladder Urothelial Carcinoma | Bladder | Bladder | Yes |
| 685 | TCGA-SO-A8JP | Testicular Germ Cell Tumors | Thyroid | Thyroid | Yes |
| 686 | TCGA-TQ-A7RR | Brain Lower Grade Glioma | Brain | Brain | Yes |
| 687 | TCGA-55-6971 | Lung Adenocarcinoma | Lung | Lung | Yes |
| 688 | TCGA-EL-A3ZP | Thyroid Carcinoma | Thymus | Thymus | Yes |
| 689 | TCGA-OR-A5LP | Adrenocortical Carcinoma | Adrenal Gland | Adrenal Gland | Yes |
| 690 | TCGA-85-7950 | Lung Squamous Cell Carcinoma | Lung | Lung | Yes |
| 691 | TCGA-X9-A973 | Sarcoma | Skin | Skin | Yes |
| 692 | TCGA-HE-A5NF | Kidney Renal Papillary Cell Carcinoma | Kidney | Kidney | Yes |
| 693 | TCGA-CV-7422 | Head and Neck Squamous Cell Carcinoma | Head and Neck | Head and Neck | Yes |
| 694 | TCGA-IQ-A61G | Head and Neck Squamous Cell Carcinoma | Head and Neck | Head and Neck | Yes |
| 695 | TCGA-BA-5149 | Head and Neck Squamous Cell Carcinoma | Head and Neck | Head and Neck | Yes |
| 696 | TCGA-M7-A71Y | Prostate Adenocarcinoma | Prostate | Prostate | Yes |
| 697 | TCGA-A2-A3XY | Breast Invasive Carcinoma | Breast | Breast | Yes |
| 698 | TCGA-FG-6692 | Brain Lower Grade Glioma | Brain | Brain | Yes |
| 699 | TCGA-55-7728 | Lung Adenocarcinoma | Lung | Lung | Yes |
| 700 | TCGA-22-A5C4 | Lung Squamous Cell Carcinoma | Pancreas | Pancreas | Yes |
| 701 | TCGA-FG-A4MU | Brain Lower Grade Glioma | Brain | Brain | Yes |
| 702 | TCGA-GM-A2DC | Breast Invasive Carcinoma | Breast | Breast | Yes |
| 703 | TCGA-JY-A93D | Esophageal Carcinoma | Esophagus | Stomach | No |
| 704 | TCGA-DD-AACJ | Liver Hepatocellular Carcinoma | Liver | Liver | Yes |
| 705 | TCGA-IB-7893 | Pancreatic Adenocarcinoma | Adrenal Gland | Adrenal Gland | Yes |
| 706 | TCGA-HU-A4HD | Stomach Adenocarcinoma | Thyroid | Thyroid | Yes |
| 707 | TCGA-HT-7692 | Brain Lower Grade Glioma | Brain | Brain | Yes |
| 708 | TCGA-DV-A4VX | Kidney Renal Clear Cell Carcinoma | Kidney | Kidney | Yes |
| 709 | TCGA-DM-A0X9 | Colon Adenocarcinoma | Colorectal | Colorectal | Yes |
| 710 | TCGA-DE-A0XZ | Thyroid Carcinoma | Thyroid | Thyroid | Yes |
| 711 | TCGA-EL-A4K2 | Thyroid Carcinoma | Thyroid | Thyroid | Yes |
| 712 | TCGA-VQ-A8P5 | Stomach Adenocarcinoma | Testis | Testis | Yes |
| 713 | TCGA-5P-A9JU | Kidney Renal Papillary Cell Carcinoma | Brain | Brain | Yes |
| 714 | TCGA-19-5950 | Glioblastoma Multiforme | Head and Neck | Head and Neck | Yes |
| 715 | TCGA-HT-8012 | Brain Lower Grade Glioma | Liver | Liver | Yes |
| 716 | TCGA-AN-A0XS | Breast Invasive Carcinoma | Breast | Breast | Yes |
| 717 | TCGA-HC-8261 | Prostate Adenocarcinoma | Colorectal | Colorectal | Yes |
| 718 | TCGA-HC-A8D0 | Prostate Adenocarcinoma | Prostate | Prostate | Yes |
| 719 | TCGA-HI-7169 | Prostate Adenocarcinoma | Prostate | Prostate | Yes |
| 720 | TCGA-L1-A7W4 | Pancreatic Adenocarcinoma | Adrenal Gland | Adrenal Gland | Yes |
| 721 | TCGA-EM-A2OY | Thyroid Carcinoma | Thymus | Thymus | Yes |
| 722 | TCGA-LL-A440 | Breast Invasive Carcinoma | Breast | Breast | Yes |
| 723 | TCGA-GM-A2DL | Breast Invasive Carcinoma | Breast | Breast | Yes |
| 724 | TCGA-DX-AB3B | Sarcoma | Soft Tissue | Soft Tissue | Yes |
| 725 | TCGA-CV-7415 | Head and Neck Squamous Cell Carcinoma | Head and Neck | Head and Neck | Yes |
| 726 | TCGA-A2-A0YD | Breast Invasive Carcinoma | Breast | Breast | Yes |
| 727 | TCGA-IB-7887 | Pancreatic Adenocarcinoma | Adrenal Gland | Adrenal Gland | Yes |
| 728 | TCGA-ET-A3BU | Thyroid Carcinoma | Thyroid | Thyroid | Yes |
| 729 | TCGA-HN-A2NL | Breast Invasive Carcinoma | Breast | Breast | Yes |
| 730 | TCGA-55-8511 | Lung Adenocarcinoma | Lung | Lung | Yes |
| 731 | TCGA-FD-A3SQ | Bladder Urothelial Carcinoma | Bladder | Bladder | Yes |
| 732 | TCGA-DC-6683 | Rectum Adenocarcinoma | Soft Tissue | Soft Tissue | Yes |
| 733 | TCGA-2G-AAGX | Testicular Germ Cell Tumors | Thyroid | Thyroid | Yes |
| 734 | TCGA-AD-6888 | Colon Adenocarcinoma | Colorectal | Colorectal | Yes |
| 735 | TCGA-97-A4M1 | Lung Adenocarcinoma | Lung | Lung | Yes |
| 736 | TCGA-2K-A9WE | Kidney Renal Papillary Cell Carcinoma | Brain | Brain | Yes |
| 737 | TCGA-CZ-5988 | Kidney Renal Clear Cell Carcinoma | Kidney | Kidney | Yes |
| 738 | TCGA-US-A774 | Pancreatic Adenocarcinoma | Prostate | Prostate | Yes |
| 739 | TCGA-G9-6329 | Prostate Adenocarcinoma | Soft Tissue | Soft Tissue | Yes |
| 740 | TCGA-BJ-A2N7 | Thyroid Carcinoma | Thyroid | Thyroid | Yes |
| 741 | TCGA-VN-A88Q | Prostate Adenocarcinoma | Prostate | Prostate | Yes |
| 742 | TCGA-D7-5579 | Stomach Adenocarcinoma | Testis | Testis | Yes |
| 743 | TCGA-BL-A13J | Bladder Urothelial Carcinoma | Bladder | Bladder | Yes |
| 744 | TCGA-BR-6801 | Stomach Adenocarcinoma | Stomach | Stomach | Yes |
| 745 | TCGA-DX-A3U6 | Sarcoma | Stomach | Stomach | Yes |
| 746 | TCGA-HC-7736 | Prostate Adenocarcinoma | Soft Tissue | Soft Tissue | Yes |
| 747 | TCGA-O2-A52N | Lung Squamous Cell Carcinoma | Lung | Lung | Yes |
| 748 | TCGA-FY-A4B3 | Thyroid Carcinoma | Thyroid | Thyroid | Yes |
| 749 | TCGA-EL-A3ZG | Thyroid Carcinoma | Thyroid | Thyroid | Yes |
| 750 | TCGA-CV-6939 | Head and Neck Squamous Cell Carcinoma | Head and Neck | Head and Neck | Yes |
| 751 | TCGA-AR-A0TQ | Breast Invasive Carcinoma | Breast | Breast | Yes |
| 752 | TCGA-A6-3809 | Colon Adenocarcinoma | Colorectal | Colorectal | Yes |
| 753 | TCGA-UZ-A9Q0 | Kidney Renal Papillary Cell Carcinoma | Kidney | Kidney | Yes |
| 754 | TCGA-70-6722 | Lung Squamous Cell Carcinoma | Lung | Lung | Yes |
| 755 | TCGA-55-8096 | Lung Adenocarcinoma | Lung | Lung | Yes |
| 756 | TCGA-TS-A7PB | Mesothelioma | Pancreas | Pancreas | Yes |
| 757 | TCGA-EW-A6S9 | Breast Invasive Carcinoma | Breast | Breast | Yes |
| 758 | TCGA-D8-A1XB | Breast Invasive Carcinoma | Breast | Breast | Yes |
| 759 | TCGA-56-7730 | Lung Squamous Cell Carcinoma | Pancreas | Pancreas | Yes |
| 760 | TCGA-DD-A11D | Liver Hepatocellular Carcinoma | Liver | Liver | Yes |
| 761 | TCGA-X6-A7WA | Sarcoma | Soft Tissue | Soft Tissue | Yes |
| 762 | TCGA-VQ-A94R | Stomach Adenocarcinoma | Testis | Testis | Yes |
| 763 | TCGA-V1-A9ZG | Prostate Adenocarcinoma | Colorectal | Colorectal | Yes |
| 764 | TCGA-CM-4744 | Colon Adenocarcinoma | Colorectal | Colorectal | Yes |
| 765 | TCGA-F9-A7VF | Kidney Renal Papillary Cell Carcinoma | Kidney | Kidney | Yes |
| 766 | TCGA-50-6592 | Lung Adenocarcinoma | Lung | Lung | Yes |
| 767 | TCGA-BA-A6DB | Head and Neck Squamous Cell Carcinoma | Kidney | Kidney | Yes |
| 768 | TCGA-VP-A879 | Prostate Adenocarcinoma | Prostate | Prostate | Yes |
| 769 | TCGA-CV-6433 | Head and Neck Squamous Cell Carcinoma | Head and Neck | Head and Neck | Yes |
| 770 | TCGA-KM-8438 | Kidney Chromophobe | Kidney | Kidney | Yes |
| 771 | TCGA-FA-A4BB | Lymphoid Neoplasm Diffuse Large B-cell Lymphoma | Esophagus | Esophagus | Yes |
| 772 | TCGA-HU-A4GD | Stomach Adenocarcinoma | Stomach | Pancreas | No |
| 773 | TCGA-EB-A41B | Skin Cutaneous Melanoma | Stomach | Stomach | Yes |
| 774 | TCGA-BR-7715 | Stomach Adenocarcinoma | Testis | Testis | Yes |
| 775 | TCGA-ZH-A8Y4 | Cholangiocarcinoma | Colorectal | Colorectal | Yes |
| 776 | TCGA-BQ-5878 | Kidney Renal Papillary Cell Carcinoma | Kidney | Kidney | Yes |
| 777 | TCGA-SP-A6QG | Pheochromocytoma and Paraganglioma | Prostate | Prostate | Yes |
| 778 | TCGA-49-6745 | Lung Adenocarcinoma | Lung | Lung | Yes |
| 779 | TCGA-DX-A7EU | Sarcoma | Stomach | Stomach | Yes |
| 780 | TCGA-MS-A51U | Breast Invasive Carcinoma | Breast | Breast | Yes |
| 781 | TCGA-XF-AAMG | Bladder Urothelial Carcinoma | Bladder | Bladder | Yes |
| 782 | TCGA-L5-A893 | Esophageal Carcinoma | Brain | Brain | Yes |
| 783 | TCGA-VQ-A8P3 | Stomach Adenocarcinoma | Testis | Testis | Yes |
| 784 | TCGA-CQ-6222 | Head and Neck Squamous Cell Carcinoma | Head and Neck | Head and Neck | Yes |
| 785 | TCGA-QR-A6GS | Pheochromocytoma and Paraganglioma | Prostate | Prostate | Yes |
| 786 | TCGA-AH-6544 | Rectum Adenocarcinoma | Soft Tissue | Soft Tissue | Yes |
| 787 | TCGA-NQ-A638 | Mesothelioma | Pancreas | Pancreas | Yes |
| 788 | TCGA-98-A53B | Lung Squamous Cell Carcinoma | Lung | Lung | Yes |
| 789 | TCGA-BP-5196 | Kidney Renal Clear Cell Carcinoma | Kidney | Kidney | Yes |
| 790 | TCGA-DB-5270 | Brain Lower Grade Glioma | Brain | Brain | Yes |
| 791 | TCGA-HC-7081 | Prostate Adenocarcinoma | Soft Tissue | Soft Tissue | Yes |
| 792 | TCGA-L5-A4OT | Esophageal Carcinoma | Brain | Brain | Yes |
| 793 | TCGA-DD-A73G | Liver Hepatocellular Carcinoma | Liver | Liver | Yes |
| 794 | TCGA-VP-A87C | Prostate Adenocarcinoma | Prostate | Prostate | Yes |
| 795 | TCGA-DD-A114 | Liver Hepatocellular Carcinoma | Lung | Lung | Yes |
| 796 | TCGA-KN-8436 | Kidney Chromophobe | Kidney | Kidney | Yes |
| 797 | TCGA-06-6695 | Glioblastoma Multiforme | Head and Neck | Head and Neck | Yes |
| 798 | TCGA-VR-A8EY | Esophageal Carcinoma | Brain | Brain | Yes |
| 799 | TCGA-EI-6881 | Rectum Adenocarcinoma | Soft Tissue | Soft Tissue | Yes |
| 800 | TCGA-S7-A7WU | Pheochromocytoma and Paraganglioma | Prostate | Prostate | Yes |
| 801 | TCGA-KS-A41F | Thyroid Carcinoma | Thymus | Thymus | Yes |
| 802 | TCGA-TQ-A7RV | Brain Lower Grade Glioma | Brain | Brain | Yes |
| 803 | TCGA-QT-A5XM | Pheochromocytoma and Paraganglioma | Prostate | Prostate | Yes |
| 804 | TCGA-69-7974 | Lung Adenocarcinoma | Lung | Lung | Yes |
| 805 | TCGA-KO-8409 | Kidney Chromophobe | Kidney | Kidney | Yes |
| 806 | TCGA-78-7143 | Lung Adenocarcinoma | Lung | Lung | Yes |
| 807 | TCGA-G4-6307 | Colon Adenocarcinoma | Colorectal | Colorectal | Yes |
| 808 | TCGA-OR-A5JV | Adrenocortical Carcinoma | Adrenal Gland | Adrenal Gland | Yes |
| 809 | TCGA-DD-AAVP | Liver Hepatocellular Carcinoma | Liver | Liver | Yes |
| 810 | TCGA-06-5417 | Glioblastoma Multiforme | Brain | Brain | Yes |
| 811 | TCGA-A7-A26E | Breast Invasive Carcinoma | Breast | Breast | Yes |
| 812 | TCGA-ET-A39N | Thyroid Carcinoma | Thyroid | Thyroid | Yes |
| 813 | TCGA-DM-A282 | Colon Adenocarcinoma | Colorectal | Colorectal | Yes |
| 814 | TCGA-FG-A6J1 | Brain Lower Grade Glioma | Brain | Brain | Yes |
| 815 | TCGA-22-5472 | Lung Squamous Cell Carcinoma | Lung | Lung | Yes |
| 816 | TCGA-DB-5278 | Brain Lower Grade Glioma | Brain | Brain | Yes |
| 817 | TCGA-CV-6952 | Head and Neck Squamous Cell Carcinoma | Head and Neck | Head and Neck | Yes |
| 818 | TCGA-2G-AAH4 | Testicular Germ Cell Tumors | Thyroid | Thyroid | Yes |
| 819 | TCGA-E9-A1R6 | Breast Invasive Carcinoma | Breast | Breast | Yes |
| 820 | TCGA-AR-A2LO | Breast Invasive Carcinoma | Breast | Breast | Yes |
| 821 | TCGA-85-A4CL | Lung Squamous Cell Carcinoma | Pancreas | Pancreas | Yes |
| 822 | TCGA-LD-A66U | Breast Invasive Carcinoma | Breast | Breast | Yes |
| 823 | TCGA-EL-A4JX | Thyroid Carcinoma | Thymus | Thymus | Yes |
| 824 | TCGA-B0-5119 | Kidney Renal Clear Cell Carcinoma | Kidney | Kidney | Yes |
| 825 | TCGA-HC-7232 | Prostate Adenocarcinoma | Prostate | Prostate | Yes |
| 826 | TCGA-D8-A1JE | Breast Invasive Carcinoma | Breast | Breast | Yes |
| 827 | TCGA-FD-A3SJ | Bladder Urothelial Carcinoma | Bladder | Bladder | Yes |
| 828 | TCGA-77-7138 | Lung Squamous Cell Carcinoma | Lung | Lung | Yes |
| 829 | TCGA-DB-A64V | Brain Lower Grade Glioma | Brain | Brain | Yes |
| 830 | TCGA-QR-A70E | Pheochromocytoma and Paraganglioma | Prostate | Prostate | Yes |
| 831 | TCGA-D7-A4YT | Stomach Adenocarcinoma | Stomach | Stomach | Yes |
| 832 | TCGA-K4-A6FZ | Bladder Urothelial Carcinoma | Bladder | Bladder | Yes |
| 833 | TCGA-DD-AADW | Liver Hepatocellular Carcinoma | Liver | Liver | Yes |
| 834 | TCGA-CQ-5327 | Head and Neck Squamous Cell Carcinoma | Head and Neck | Head and Neck | Yes |
| 835 | TCGA-EM-A1CT | Thyroid Carcinoma | Thyroid | Thyroid | Yes |
| 836 | TCGA-B0-5812 | Kidney Renal Clear Cell Carcinoma | Kidney | Kidney | Yes |
| 837 | TCGA-FG-A87N | Brain Lower Grade Glioma | Brain | Brain | Yes |
| 838 | TCGA-XF-AAN2 | Bladder Urothelial Carcinoma | Bladder | Bladder | Yes |
| 839 | TCGA-DU-A6S6 | Brain Lower Grade Glioma | Liver | Liver | Yes |
| 840 | TCGA-91-6849 | Lung Adenocarcinoma | Lung | Lung | Yes |
| 841 | TCGA-DU-5874 | Brain Lower Grade Glioma | Brain | Brain | Yes |
| 842 | TCGA-49-AARE | Lung Adenocarcinoma | Lung | Lung | Yes |
| 843 | TCGA-CN-6998 | Head and Neck Squamous Cell Carcinoma | Head and Neck | Head and Neck | Yes |
| 844 | TCGA-55-7227 | Lung Adenocarcinoma | Lung | Lung | Yes |
| 845 | TCGA-CS-5397 | Brain Lower Grade Glioma | Brain | Brain | Yes |
| 846 | TCGA-2G-AALQ |  | Thyroid | Thyroid | Yes |
| 847 | TCGA-RY-A847 | Brain Lower Grade Glioma | Liver | Liver | Yes |
| 848 | TCGA-KK-A8I8 | Prostate Adenocarcinoma | Prostate | Prostate | Yes |
| 849 | TCGA-EW-A1P5 | Breast Invasive Carcinoma | Breast | Breast | Yes |
| 850 | TCGA-2G-AALR |  | Thyroid | Thyroid | Yes |
| 851 | TCGA-QK-A8ZB | Head and Neck Squamous Cell Carcinoma | Head and Neck | Head and Neck | Yes |
| 852 | TCGA-BA-A6DL | Head and Neck Squamous Cell Carcinoma | Head and Neck | Head and Neck | Yes |
| 853 | TCGA-KK-A7AU | Prostate Adenocarcinoma | Prostate | Prostate | Yes |
| 854 | TCGA-EJ-5506 | Prostate Adenocarcinoma | Soft Tissue | Soft Tissue | Yes |
| 855 | TCGA-DK-A3IN | Bladder Urothelial Carcinoma | Bladder | Bladder | Yes |
| 856 | TCGA-W5-AA2Q | Cholangiocarcinoma | Colorectal | Colorectal | Yes |
| 857 | TCGA-53-A4EZ | Lung Adenocarcinoma | Lung | Lung | Yes |
| 858 | TCGA-QH-A6X3 | Brain Lower Grade Glioma | Brain | Brain | Yes |
| 859 | TCGA-D6-A74Q | Head and Neck Squamous Cell Carcinoma | Head and Neck | Head and Neck | Yes |
| 860 | TCGA-AZ-6603 | Colon Adenocarcinoma | Colorectal | Colorectal | Yes |
| 861 | TCGA-GS-A9U4 | Lymphoid Neoplasm Diffuse Large B-cell Lymphoma | Esophagus | Esophagus | Yes |
| 862 | TCGA-ZG-A9LU | Prostate Adenocarcinoma | Prostate | Prostate | Yes |
| 863 | TCGA-DX-A48O | Sarcoma | Skin | Skin | Yes |
| 864 | TCGA-E3-A3E1 | Thyroid Carcinoma | Thymus | Thymus | Yes |
| 865 | TCGA-HC-7750 | Prostate Adenocarcinoma | Prostate | Prostate | Yes |
| 866 | TCGA-BR-7723 | Stomach Adenocarcinoma | Testis | Testis | Yes |
| 867 | TCGA-EL-A3CN | Thyroid Carcinoma | Thyroid | Thyroid | Yes |
| 868 | TCGA-VQ-A8PZ | Stomach Adenocarcinoma | Thyroid | Thyroid | Yes |
| 869 | TCGA-KO-8404 | Kidney Chromophobe | Kidney | Kidney | Yes |
| 870 | TCGA-CD-5813 | Stomach Adenocarcinoma | Stomach | Stomach | Yes |
| 871 | TCGA-XF-A9SG | Bladder Urothelial Carcinoma | Bladder | Bladder | Yes |
| 872 | TCGA-AF-4110 | Rectum Adenocarcinoma | Soft Tissue | Soft Tissue | Yes |
| 873 | TCGA-49-6744 | Lung Adenocarcinoma | Lung | Lung | Yes |
| 874 | TCGA-AR-A1AI | Breast Invasive Carcinoma | Breast | Breast | Yes |
| 875 | TCGA-R6-A6DN | Esophageal Carcinoma | Esophagus | Stomach | No |
| 876 | TCGA-E2-A10C | Breast Invasive Carcinoma | Breast | Breast | Yes |
| 877 | TCGA-DQ-7589 | Head and Neck Squamous Cell Carcinoma | Head and Neck | Head and Neck | Yes |
| 878 | TCGA-60-2709 | Lung Squamous Cell Carcinoma | Pancreas | Pancreas | Yes |
| 879 | TCGA-75-7031 | Lung Adenocarcinoma | Lung | Lung | Yes |
| 880 | TCGA-EJ-5503 | Prostate Adenocarcinoma | Colorectal | Colorectal | Yes |
| 881 | TCGA-BH-A0RX | Breast Invasive Carcinoma | Breast | Breast | Yes |
| 882 | TCGA-CN-4729 | Head and Neck Squamous Cell Carcinoma | Kidney | Kidney | Yes |
| 883 | TCGA-CJ-4905 | Kidney Renal Clear Cell Carcinoma | Kidney | Kidney | Yes |
| 884 | TCGA-3M-AB47 | Stomach Adenocarcinoma | Thyroid | Thyroid | Yes |
| 885 | TCGA-AR-A1AP | Breast Invasive Carcinoma | Breast | Breast | Yes |
| 886 | TCGA-B6-A0I1 | Breast Invasive Carcinoma | Breast | Breast | Yes |
| 887 | TCGA-EB-A551 | Skin Cutaneous Melanoma | Stomach | Stomach | Yes |
| 888 | TCGA-DM-A28F | Colon Adenocarcinoma | Colorectal | Colorectal | Yes |
| 889 | TCGA-CM-6162 | Colon Adenocarcinoma | Colorectal | Colorectal | Yes |
| 890 | TCGA-YL-A9WJ | Prostate Adenocarcinoma | Prostate | Prostate | Yes |
| 891 | TCGA-MP-A4T7 | Lung Adenocarcinoma | Lung | Lung | Yes |
| 892 | TCGA-E2-A1LI | Breast Invasive Carcinoma | Breast | Breast | Yes |
| 893 | TCGA-CZ-5984 | Kidney Renal Clear Cell Carcinoma | Kidney | Kidney | Yes |
| 894 | TCGA-IN-A7NT | Stomach Adenocarcinoma | Stomach | Stomach | Yes |
| 895 | TCGA-VQ-A8PU | Stomach Adenocarcinoma | Stomach | Stomach | Yes |
| 896 | TCGA-91-6829 | Lung Adenocarcinoma | Lung | Lung | Yes |
| 897 | TCGA-SB-A6J6 | Testicular Germ Cell Tumors | Thyroid | Thyroid | Yes |
| 898 | TCGA-BH-A42U | Breast Invasive Carcinoma | Breast | Breast | Yes |
| 899 | TCGA-BD-A3ER | Liver Hepatocellular Carcinoma | Liver | Liver | Yes |
| 900 | TCGA-DD-A119 | Liver Hepatocellular Carcinoma | Lung | Lung | Yes |
| 901 | TCGA-A4-7286 | Kidney Renal Papillary Cell Carcinoma | Kidney | Kidney | Yes |
| 902 | TCGA-VQ-A927 | Stomach Adenocarcinoma | Stomach | Stomach | Yes |
| 903 | TCGA-FX-A2QS | Sarcoma | Skin | Skin | Yes |
| 904 | TCGA-MZ-A6I9 | Head and Neck Squamous Cell Carcinoma | Head and Neck | Head and Neck | Yes |
| 905 | TCGA-ZF-AA54 | Bladder Urothelial Carcinoma | Bladder | Bladder | Yes |
| 906 | TCGA-US-A779 | Pancreatic Adenocarcinoma | Adrenal Gland | Adrenal Gland | Yes |
| 907 | TCGA-CE-A13K | Thyroid Carcinoma | Thymus | Thymus | Yes |
| 908 | TCGA-GM-A2DI | Breast Invasive Carcinoma | Breast | Breast | Yes |
| 909 | TCGA-F5-6812 | Rectum Adenocarcinoma | Soft Tissue | Soft Tissue | Yes |
| 910 | TCGA-BH-A0B2 |  | Breast | Breast | Yes |
| 911 | TCGA-L6-A4ET | Thyroid Carcinoma | Thyroid | Thyroid | Yes |
| 912 | TCGA-H2-A26U | Thyroid Carcinoma | Thyroid | Thyroid | Yes |
| 913 | TCGA-D8-A1XY | Breast Invasive Carcinoma | Breast | Breast | Yes |
| 914 | TCGA-S9-A6WH | Brain Lower Grade Glioma | Brain | Brain | Yes |
| 915 | TCGA-74-6581 | Glioblastoma Multiforme | Head and Neck | Head and Neck | Yes |
| 916 | TCGA-BR-A4J4 | Stomach Adenocarcinoma | Testis | Testis | Yes |
| 917 | TCGA-FD-A6TK | Bladder Urothelial Carcinoma | Bladder | Bladder | Yes |
| 918 | TCGA-2H-A9GO | Esophageal Carcinoma | Brain | Brain | Yes |
| 919 | TCGA-06-6390 | Glioblastoma Multiforme | Head and Neck | Head and Neck | Yes |
| 920 | TCGA-GM-A5PV | Breast Invasive Carcinoma | Breast | Breast | Yes |
| 921 | TCGA-94-8491 | Lung Squamous Cell Carcinoma | Pancreas | Pancreas | Yes |
| 922 | TCGA-HU-A4GH | Stomach Adenocarcinoma | Testis | Testis | Yes |
| 923 | TCGA-2G-AAFV | Testicular Germ Cell Tumors | Thyroid | Thyroid | Yes |
| 924 | TCGA-MQ-A4LJ | Mesothelioma | Pancreas | Pancreas | Yes |
| 925 | TCGA-78-7153 | Lung Adenocarcinoma | Lung | Lung | Yes |
| 926 | TCGA-AC-A2FB | Breast Invasive Carcinoma | Breast | Breast | Yes |
| 927 | TCGA-B8-A7U6 | Kidney Renal Clear Cell Carcinoma | Kidney | Kidney | Yes |
| 928 | TCGA-CN-6995 | Head and Neck Squamous Cell Carcinoma | Head and Neck | Head and Neck | Yes |
| 929 | TCGA-D6-6515 | Head and Neck Squamous Cell Carcinoma | Kidney | Kidney | Yes |
| 930 | TCGA-NC-A5HG | Lung Squamous Cell Carcinoma | Lung | Lung | Yes |
| 931 | TCGA-BR-8384 | Stomach Adenocarcinoma | Stomach | Stomach | Yes |
| 932 | TCGA-KN-8431 | Kidney Chromophobe | Kidney | Kidney | Yes |
| 933 | TCGA-AZ-4308 | Colon Adenocarcinoma | Colorectal | Colorectal | Yes |
| 934 | TCGA-B0-4697 | Kidney Renal Clear Cell Carcinoma | Kidney | Kidney | Yes |
| 935 | TCGA-BL-A13I | Bladder Urothelial Carcinoma | Bladder | Bladder | Yes |
| 936 | TCGA-IQ-A61O | Head and Neck Squamous Cell Carcinoma | Kidney | Kidney | Yes |
| 937 | TCGA-MH-A561 | Kidney Renal Papillary Cell Carcinoma | Brain | Brain | Yes |
| 938 | TCGA-L5-A4OM | Esophageal Carcinoma | Brain | Brain | Yes |
| 939 | TCGA-GK-A6C7 | Kidney Renal Clear Cell Carcinoma | Kidney | Kidney | Yes |
| 940 | TCGA-QR-A6H1 | Pheochromocytoma and Paraganglioma | Prostate | Prostate | Yes |
| 941 | TCGA-34-5232 | Lung Squamous Cell Carcinoma | Lung | Lung | Yes |
| 942 | TCGA-ZM-AA0F | Testicular Germ Cell Tumors | Thyroid | Thyroid | Yes |
| 943 | TCGA-EW-A1P4 | Breast Invasive Carcinoma | Breast | Breast | Yes |
| 944 | TCGA-DH-5142 | Brain Lower Grade Glioma | Liver | Liver | Yes |
| 945 | TCGA-PC-A5DO | Sarcoma | Skin | Skin | Yes |
| 946 | TCGA-BH-A0DP | Breast Invasive Carcinoma | Breast | Breast | Yes |
| 947 | TCGA-CR-6492 | Head and Neck Squamous Cell Carcinoma | Head and Neck | Head and Neck | Yes |
| 948 | TCGA-BR-A4J1 | Stomach Adenocarcinoma | Thyroid | Thyroid | Yes |
| 949 | TCGA-B6-A0X5 | Breast Invasive Carcinoma | Breast | Breast | Yes |
| 950 | TCGA-Y8-A898 | Kidney Renal Papillary Cell Carcinoma | Kidney | Kidney | Yes |
| 951 | TCGA-CV-7242 | Head and Neck Squamous Cell Carcinoma | Head and Neck | Head and Neck | Yes |
| 952 | TCGA-NH-A50V | Colon Adenocarcinoma | Esophagus | Esophagus | Yes |
| 953 | TCGA-CN-5369 | Head and Neck Squamous Cell Carcinoma | Kidney | Kidney | Yes |
| 954 | TCGA-VR-A8EO | Esophageal Carcinoma | Esophagus | Esophagus | Yes |
| 955 | TCGA-86-8054 | Lung Adenocarcinoma | Lung | Lung | Yes |
| 956 | TCGA-AD-6899 | Colon Adenocarcinoma | Colorectal | Colorectal | Yes |
| 957 | TCGA-CN-6010 | Head and Neck Squamous Cell Carcinoma | Head and Neck | Head and Neck | Yes |
| 958 | TCGA-B0-4816 | Kidney Renal Clear Cell Carcinoma | Kidney | Kidney | Yes |
| 959 | TCGA-YL-A8HK | Prostate Adenocarcinoma | Prostate | Prostate | Yes |
| 960 | TCGA-DD-AAC8 | Liver Hepatocellular Carcinoma | Liver | Soft Tissue | No |
| 961 | TCGA-HI-7170 | Prostate Adenocarcinoma | Prostate | Prostate | Yes |
| 962 | TCGA-2G-AAGO | Testicular Germ Cell Tumors | Thyroid | Thyroid | Yes |
| 963 | TCGA-D7-A6F2 | Stomach Adenocarcinoma | Stomach | Stomach | Yes |
| 964 | TCGA-HZ-8002 | Pancreatic Adenocarcinoma | Adrenal Gland | Adrenal Gland | Yes |
| 965 | TCGA-BR-A4QI | Stomach Adenocarcinoma | Stomach | Stomach | Yes |
| 966 | TCGA-2H-A9GI | Esophageal Carcinoma | Esophagus | Head and Neck | No |
| 967 | TCGA-CN-A63V | Head and Neck Squamous Cell Carcinoma | Head and Neck | Breast | No |
| 968 | TCGA-E9-A243 | Breast Invasive Carcinoma | Breast | Breast | Yes |
| 969 | TCGA-UB-AA0U | Liver Hepatocellular Carcinoma | Liver | Liver | Yes |
| 970 | TCGA-85-A510 | Lung Squamous Cell Carcinoma | Pancreas | Pancreas | Yes |
| 971 | TCGA-KL-8346 | Kidney Chromophobe | Kidney | Kidney | Yes |
| 972 | TCGA-E9-A226 | Breast Invasive Carcinoma | Breast | Breast | Yes |
| 973 | TCGA-E2-A1II | Breast Invasive Carcinoma | Breast | Breast | Yes |
| 974 | TCGA-DU-7294 | Brain Lower Grade Glioma | Brain | Brain | Yes |
| 975 | TCGA-KL-8328 | Kidney Chromophobe | Kidney | Kidney | Yes |
| 976 | TCGA-BQ-7056 | Kidney Renal Papillary Cell Carcinoma | Brain | Brain | Yes |
| 977 | TCGA-TM-A84L | Brain Lower Grade Glioma | Liver | Liver | Yes |
| 978 | TCGA-CW-5590 | Kidney Renal Clear Cell Carcinoma | Kidney | Kidney | Yes |
| 979 | TCGA-77-6842 | Lung Squamous Cell Carcinoma | Pancreas | Pancreas | Yes |
| 980 | TCGA-DC-5869 | Rectum Adenocarcinoma | Soft Tissue | Soft Tissue | Yes |
| 981 | TCGA-2G-AAHC | Testicular Germ Cell Tumors | Thyroid | Thyroid | Yes |
| 982 | TCGA-CD-A4MI | Stomach Adenocarcinoma | Stomach | Stomach | Yes |
| 983 | TCGA-E5-A4U1 | Bladder Urothelial Carcinoma | Bladder | Bladder | Yes |
| 984 | TCGA-FD-A43S | Bladder Urothelial Carcinoma | Bladder | Bladder | Yes |
| 985 | TCGA-DK-A3IV | Bladder Urothelial Carcinoma | Bladder | Bladder | Yes |
| 986 | TCGA-DJ-A3VI | Thyroid Carcinoma | Thymus | Thymus | Yes |
| 987 | TCGA-QT-A5XJ | Pheochromocytoma and Paraganglioma | Prostate | Prostate | Yes |
| 988 | TCGA-LN-A9FQ | Esophageal Carcinoma | Esophagus | Head and Neck | No |
| 989 | TCGA-GM-A2DO | Breast Invasive Carcinoma | Breast | Breast | Yes |
| 990 | TCGA-KO-8413 | Kidney Chromophobe | Kidney | Kidney | Yes |
| 991 | TCGA-DD-AAVW | Liver Hepatocellular Carcinoma | Liver | Liver | Yes |
| 992 | TCGA-DJ-A1QM | Thyroid Carcinoma | Thyroid | Thyroid | Yes |
| 993 | TCGA-CV-7438 | Head and Neck Squamous Cell Carcinoma | Head and Neck | Head and Neck | Yes |
| 994 | TCGA-XE-AANV | Testicular Germ Cell Tumors | Thyroid | Thyroid | Yes |
| 995 | TCGA-CV-6937 | Head and Neck Squamous Cell Carcinoma | Head and Neck | Head and Neck | Yes |
| 996 | TCGA-D7-A6ET | Stomach Adenocarcinoma | Testis | Testis | Yes |
| 997 | TCGA-86-7953 | Lung Adenocarcinoma | Lung | Lung | Yes |
| 998 | TCGA-BR-7197 | Stomach Adenocarcinoma | Testis | Testis | Yes |
| 999 | TCGA-BQ-7048 | Kidney Renal Papillary Cell Carcinoma | Kidney | Kidney | Yes |
| 1000 | TCGA-S9-A7IQ | Brain Lower Grade Glioma | Brain | Brain | Yes |
| 1001 | TCGA-E2-A10F | Breast Invasive Carcinoma | Breast | Breast | Yes |
| 1002 | TCGA-QQ-A8VH | Sarcoma | Skin | Skin | Yes |
| 1003 | TCGA-WD-A7RX | Cholangiocarcinoma | Colorectal | Colorectal | Yes |
| 1004 | TCGA-06-0190 | Glioblastoma Multiforme | Head and Neck | Head and Neck | Yes |
| 1005 | TCGA-HC-7752 | Prostate Adenocarcinoma | Prostate | Prostate | Yes |
| 1006 | TCGA-DJ-A2QB | Thyroid Carcinoma | Thyroid | Thyroid | Yes |
| 1007 | TCGA-ET-A39K | Thyroid Carcinoma | Thyroid | Thyroid | Yes |
| 1008 | TCGA-E2-A1BD | Breast Invasive Carcinoma | Breast | Breast | Yes |
| 1009 | TCGA-D5-6929 | Colon Adenocarcinoma | Colorectal | Colorectal | Yes |
| 1010 | TCGA-MK-A4N9 | Thyroid Carcinoma | Thyroid | Thyroid | Yes |
| 1011 | TCGA-92-8064 | Lung Squamous Cell Carcinoma | Lung | Lung | Yes |
| 1012 | TCGA-BR-8370 | Stomach Adenocarcinoma | Stomach | Stomach | Yes |
| 1013 | TCGA-12-5295 | Glioblastoma Multiforme | Head and Neck | Head and Neck | Yes |
| 1014 | TCGA-G4-6309 | Colon Adenocarcinoma | Colorectal | Colorectal | Yes |
| 1015 | TCGA-GD-A3OP | Bladder Urothelial Carcinoma | Bladder | Bladder | Yes |
| 1016 | TCGA-VQ-A94U | Stomach Adenocarcinoma | Stomach | Stomach | Yes |
| 1017 | TCGA-2G-AAGN | Testicular Germ Cell Tumors | Thyroid | Thyroid | Yes |
| 1018 | TCGA-BH-A0BF | Breast Invasive Carcinoma | Breast | Breast | Yes |
| 1019 | TCGA-HW-7487 | Brain Lower Grade Glioma | Brain | Brain | Yes |
| 1020 | TCGA-BH-A5IZ | Breast Invasive Carcinoma | Breast | Breast | Yes |
| 1021 | TCGA-DJ-A2PS | Thyroid Carcinoma | Thyroid | Thyroid | Yes |
| 1022 | TCGA-V1-A9O5 | Prostate Adenocarcinoma | Prostate | Prostate | Yes |
| 1023 | TCGA-UD-AAC6 | Mesothelioma | Pancreas | Pancreas | Yes |
| 1024 | TCGA-S5-A6DX | Bladder Urothelial Carcinoma | Bladder | Bladder | Yes |
| 1025 | TCGA-CN-5370 | Head and Neck Squamous Cell Carcinoma | Head and Neck | Head and Neck | Yes |
| 1026 | TCGA-XE-A8H5 | Testicular Germ Cell Tumors | Thyroid | Thyroid | Yes |
| 1027 | TCGA-XF-AAME | Bladder Urothelial Carcinoma | Bladder | Bladder | Yes |
| 1028 | TCGA-VQ-AA6I | Stomach Adenocarcinoma | Stomach | Stomach | Yes |
| 1029 | TCGA-AR-A24U | Breast Invasive Carcinoma | Breast | Breast | Yes |
| 1030 | TCGA-CR-7393 | Head and Neck Squamous Cell Carcinoma | Head and Neck | Head and Neck | Yes |
| 1031 | TCGA-2Y-A9GZ | Liver Hepatocellular Carcinoma | Liver | Liver | Yes |
| 1032 | TCGA-AC-A3W5 | Breast Invasive Carcinoma | Breast | Breast | Yes |
| 1033 | TCGA-53-7813 | Lung Adenocarcinoma | Lung | Lung | Yes |
| 1034 | TCGA-21-5783 | Lung Squamous Cell Carcinoma | Lung | Lung | Yes |
| 1035 | TCGA-CV-6962 | Head and Neck Squamous Cell Carcinoma | Head and Neck | Head and Neck | Yes |
| 1036 | TCGA-BQ-7055 | Kidney Renal Papillary Cell Carcinoma | Kidney | Kidney | Yes |
| 1037 | TCGA-RS-A6TP | Head and Neck Squamous Cell Carcinoma | Head and Neck | Head and Neck | Yes |
| 1038 | TCGA-EW-A1OY | Breast Invasive Carcinoma | Breast | Breast | Yes |
| 1039 | TCGA-NK-A5CT | Lung Squamous Cell Carcinoma | Lung | Lung | Yes |
| 1040 | TCGA-L5-A43I | Esophageal Carcinoma | Brain | Brain | Yes |
| 1041 | TCGA-86-6562 | Lung Adenocarcinoma | Lung | Lung | Yes |
| 1042 | TCGA-77-A5G8 | Lung Squamous Cell Carcinoma | Lung | Lung | Yes |
| 1043 | TCGA-B6-A0RP | Breast Invasive Carcinoma | Breast | Breast | Yes |
| 1044 | TCGA-EL-A3CY | Thyroid Carcinoma | Thyroid | Thyroid | Yes |
| 1045 | TCGA-IN-A6RN | Stomach Adenocarcinoma | Stomach | Stomach | Yes |
| 1046 | TCGA-85-7697 | Lung Squamous Cell Carcinoma | Lung | Lung | Yes |
| 1047 | TCGA-RB-AA9M | Pancreatic Adenocarcinoma | Adrenal Gland | Adrenal Gland | Yes |
| 1048 | TCGA-AR-A0TT | Breast Invasive Carcinoma | Breast | Breast | Yes |
| 1049 | TCGA-BP-5181 | Kidney Renal Clear Cell Carcinoma | Kidney | Kidney | Yes |
| 1050 | TCGA-E9-A5FL | Breast Invasive Carcinoma | Breast | Breast | Yes |
| 1051 | TCGA-ZM-AA0E | Testicular Germ Cell Tumors | Thyroid | Thyroid | Yes |
| 1052 | TCGA-LL-A5YM | Breast Invasive Carcinoma | Breast | Breast | Yes |
| 1053 | TCGA-IP-7968 | Stomach Adenocarcinoma | Stomach | Stomach | Yes |
| 1054 | TCGA-A4-A4ZT | Kidney Renal Papillary Cell Carcinoma | Kidney | Kidney | Yes |
| 1055 | TCGA-BH-A1EU | Breast Invasive Carcinoma | Breast | Breast | Yes |
| 1056 | TCGA-HT-7616 | Brain Lower Grade Glioma | Brain | Brain | Yes |
| 1057 | TCGA-K4-A5RI | Bladder Urothelial Carcinoma | Bladder | Bladder | Yes |
| 1058 | TCGA-B0-5110 | Kidney Renal Clear Cell Carcinoma | Kidney | Kidney | Yes |
| 1059 | TCGA-AR-A1AU | Breast Invasive Carcinoma | Breast | Breast | Yes |
| 1060 | TCGA-BF-AAP1 | Skin Cutaneous Melanoma | Stomach | Stomach | Yes |
| 1061 | TCGA-69-7764 | Lung Adenocarcinoma | Lung | Lung | Yes |
| 1062 | TCGA-37-A5EL | Lung Squamous Cell Carcinoma | Lung | Lung | Yes |
| 1063 | TCGA-D8-A1JL | Breast Invasive Carcinoma | Breast | Breast | Yes |
| 1064 | TCGA-2Y-A9H6 | Liver Hepatocellular Carcinoma | Liver | Liver | Yes |
| 1065 | TCGA-J9-A8CP | Prostate Adenocarcinoma | Soft Tissue | Soft Tissue | Yes |
| 1066 | TCGA-D7-A4Z0 | Stomach Adenocarcinoma | Stomach | Stomach | Yes |
| 1067 | TCGA-YS-A95C | Mesothelioma | Pancreas | Pancreas | Yes |
| 1068 | TCGA-28-5211 | Glioblastoma Multiforme | Head and Neck | Head and Neck | Yes |
| 1069 | TCGA-D6-A6EK | Head and Neck Squamous Cell Carcinoma | Head and Neck | Head and Neck | Yes |
| 1070 | TCGA-C8-A1HN | Breast Invasive Carcinoma | Breast | Breast | Yes |
| 1071 | TCGA-B4-5377 | Kidney Renal Clear Cell Carcinoma | Kidney | Kidney | Yes |
| 1072 | TCGA-CZ-4864 | Kidney Renal Clear Cell Carcinoma | Kidney | Kidney | Yes |
| 1073 | TCGA-BQ-5885 | Kidney Renal Papillary Cell Carcinoma | Kidney | Kidney | Yes |
| 1074 | TCGA-BJ-A0ZE | Thyroid Carcinoma | Thyroid | Thyroid | Yes |
| 1075 | TCGA-YU-A90Q | Testicular Germ Cell Tumors | Thyroid | Thyroid | Yes |
| 1076 | TCGA-64-5815 | Lung Adenocarcinoma | Lung | Lung | Yes |
| 1077 | TCGA-43-8116 | Lung Squamous Cell Carcinoma | Pancreas | Pancreas | Yes |
| 1078 | TCGA-B0-5399 | Kidney Renal Clear Cell Carcinoma | Kidney | Kidney | Yes |
| 1079 | TCGA-HS-A5N8 | Sarcoma | Stomach | Stomach | Yes |
| 1080 | TCGA-CV-7254 | Head and Neck Squamous Cell Carcinoma | Head and Neck | Esophagus | No |
| 1081 | TCGA-LN-A49N | Esophageal Carcinoma | Esophagus | Esophagus | Yes |
| 1082 | TCGA-95-A4VK | Lung Adenocarcinoma | Lung | Lung | Yes |
| 1083 | TCGA-3L-AA1B | Colon Adenocarcinoma | Colorectal | Colorectal | Yes |
| 1084 | TCGA-5M-AATA |  | Colorectal | Colorectal | Yes |
| 1085 | TCGA-BH-A42T | Breast Invasive Carcinoma | Breast | Breast | Yes |
| 1086 | TCGA-BH-A0WA | Breast Invasive Carcinoma | Breast | Breast | Yes |
| 1087 | TCGA-DK-AA6S | Bladder Urothelial Carcinoma | Bladder | Bladder | Yes |
| 1088 | TCGA-AG-A036 | Rectum Adenocarcinoma | Soft Tissue | Adrenal Gland | No |
| 1089 | TCGA-AC-A5EH | Breast Invasive Carcinoma | Breast | Breast | Yes |
| 1090 | TCGA-28-5215 | Glioblastoma Multiforme | Brain | Brain | Yes |
| 1091 | TCGA-62-A46S | Lung Adenocarcinoma | Lung | Lung | Yes |
| 1092 | TCGA-BF-A5EQ | Skin Cutaneous Melanoma | Stomach | Stomach | Yes |
| 1093 | TCGA-DD-AAVS | Liver Hepatocellular Carcinoma | Liver | Liver | Yes |
| 1094 | TCGA-73-7498 | Lung Adenocarcinoma | Lung | Lung | Yes |
| 1095 | TCGA-G9-6371 | Prostate Adenocarcinoma | Prostate | Prostate | Yes |
| 1096 | TCGA-FY-A3R6 | Thyroid Carcinoma | Thyroid | Thyroid | Yes |
| 1097 | TCGA-EL-A3T3 | Thyroid Carcinoma | Thyroid | Thyroid | Yes |
| 1098 | TCGA-55-8087 | Lung Adenocarcinoma | Lung | Lung | Yes |
| 1099 | TCGA-A4-A7UZ | Kidney Renal Papillary Cell Carcinoma | Kidney | Kidney | Yes |
| 1100 | TCGA-2G-AAF8 | Testicular Germ Cell Tumors | Thyroid | Thyroid | Yes |
| 1101 | TCGA-D8-A1JJ | Breast Invasive Carcinoma | Breast | Breast | Yes |
| 1102 | TCGA-BJ-A0ZC | Thyroid Carcinoma | Thymus | Lung | No |
| 1103 | TCGA-F7-8298 | Head and Neck Squamous Cell Carcinoma | Head and Neck | Head and Neck | Yes |
| 1104 | TCGA-VR-AA4G | Esophageal Carcinoma | Esophagus | Esophagus | Yes |
| 1105 | TCGA-TM-A84J | Brain Lower Grade Glioma | Brain | Brain | Yes |
| 1106 | TCGA-CJ-6032 | Kidney Renal Clear Cell Carcinoma | Kidney | Kidney | Yes |
| 1107 | TCGA-EW-A1OZ | Breast Invasive Carcinoma | Breast | Breast | Yes |
| 1108 | TCGA-CG-5719 | Stomach Adenocarcinoma | Stomach | Stomach | Yes |
| 1109 | TCGA-NC-A5HI | Lung Squamous Cell Carcinoma | Lung | Lung | Yes |
| 1110 | TCGA-D7-6521 | Stomach Adenocarcinoma | Testis | Testis | Yes |
| 1111 | TCGA-BP-5200 | Kidney Renal Clear Cell Carcinoma | Kidney | Kidney | Yes |
| 1112 | TCGA-G8-6326 | Lymphoid Neoplasm Diffuse Large B-cell Lymphoma | Esophagus | Esophagus | Yes |
| 1113 | TCGA-AR-A24W | Breast Invasive Carcinoma | Breast | Breast | Yes |
| 1114 | TCGA-FD-A5BU | Bladder Urothelial Carcinoma | Bladder | Bladder | Yes |
| 1115 | TCGA-CV-7178 | Head and Neck Squamous Cell Carcinoma | Head and Neck | Head and Neck | Yes |
| 1116 | TCGA-HT-7879 | Brain Lower Grade Glioma | Brain | Brain | Yes |
| 1117 | TCGA-SH-A9CU | Mesothelioma | Pancreas | Pancreas | Yes |
| 1118 | TCGA-B0-4847 | Kidney Renal Clear Cell Carcinoma | Kidney | Kidney | Yes |
| 1119 | TCGA-BT-A20V | Bladder Urothelial Carcinoma | Bladder | Bladder | Yes |
| 1120 | TCGA-DD-AADP | Liver Hepatocellular Carcinoma | Lung | Lung | Yes |
| 1121 | TCGA-78-7220 | Lung Adenocarcinoma | Lung | Lung | Yes |
| 1122 | TCGA-CV-A465 | Head and Neck Squamous Cell Carcinoma | Head and Neck | Head and Neck | Yes |
| 1123 | TCGA-PJ-A5Z9 | Kidney Renal Papillary Cell Carcinoma | Brain | Brain | Yes |
| 1124 | TCGA-EJ-7793 | Prostate Adenocarcinoma | Prostate | Prostate | Yes |
| 1125 | TCGA-A1-A0SN | Breast Invasive Carcinoma | Breast | Breast | Yes |
| 1126 | TCGA-2Y-A9H9 | Liver Hepatocellular Carcinoma | Lung | Lung | Yes |
| 1127 | TCGA-AY-A54L | Colon Adenocarcinoma | Colorectal | Colorectal | Yes |
| 1128 | TCGA-94-8035 | Lung Squamous Cell Carcinoma | Lung | Lung | Yes |
| 1129 | TCGA-DU-6408 | Brain Lower Grade Glioma | Brain | Brain | Yes |
| 1130 | TCGA-EJ-5532 | Prostate Adenocarcinoma | Prostate | Prostate | Yes |
| 1131 | TCGA-22-5491 | Lung Squamous Cell Carcinoma | Lung | Lung | Yes |
| 1132 | TCGA-FA-A86F | Lymphoid Neoplasm Diffuse Large B-cell Lymphoma | Esophagus | Stomach | No |
| 1133 | TCGA-A2-A0EP | Breast Invasive Carcinoma | Breast | Breast | Yes |
| 1134 | TCGA-EU-5906 | Kidney Renal Clear Cell Carcinoma | Kidney | Kidney | Yes |
| 1135 | TCGA-RQ-A68N | Lymphoid Neoplasm Diffuse Large B-cell Lymphoma | Esophagus | Esophagus | Yes |
| 1136 | TCGA-UZ-A9PR | Kidney Renal Papillary Cell Carcinoma | Kidney | Kidney | Yes |
| 1137 | TCGA-XF-A9T6 | Bladder Urothelial Carcinoma | Bladder | Bladder | Yes |
| 1138 | TCGA-MP-A4SW | Lung Adenocarcinoma | Lung | Lung | Yes |
| 1139 | TCGA-2J-AABT | Pancreatic Adenocarcinoma | Adrenal Gland | Adrenal Gland | Yes |
| 1140 | TCGA-G9-6494 | Prostate Adenocarcinoma | Colorectal | Colorectal | Yes |
| 1141 | TCGA-R5-A805 | Stomach Adenocarcinoma | Stomach | Esophagus | No |
| 1142 | TCGA-2G-AALF |  | Thyroid | Thyroid | Yes |
| 1143 | TCGA-A6-5661 | Colon Adenocarcinoma | Colorectal | Colorectal | Yes |
| 1144 | TCGA-ZS-A9CE | Liver Hepatocellular Carcinoma | Liver | Liver | Yes |
| 1145 | TCGA-L3-A524 | Lung Squamous Cell Carcinoma | Lung | Lung | Yes |
| 1146 | TCGA-IG-A4QT | Esophageal Carcinoma | Esophagus | Esophagus | Yes |
| 1147 | TCGA-BR-8289 | Stomach Adenocarcinoma | Thyroid | Thyroid | Yes |
| 1148 | TCGA-85-8070 | Lung Squamous Cell Carcinoma | Lung | Lung | Yes |
| 1149 | TCGA-XF-AAMH | Bladder Urothelial Carcinoma | Bladder | Bladder | Yes |
| 1150 | TCGA-EL-A3GY | Thyroid Carcinoma | Thyroid | Thyroid | Yes |
| 1151 | TCGA-33-6738 | Lung Squamous Cell Carcinoma | Lung | Lung | Yes |
| 1152 | TCGA-PK-A5HB | Adrenocortical Carcinoma | Adrenal Gland | Adrenal Gland | Yes |
| 1153 | TCGA-BH-A1F6 | Breast Invasive Carcinoma | Breast | Breast | Yes |
| 1154 | TCGA-CV-6961 | Head and Neck Squamous Cell Carcinoma | Head and Neck | Head and Neck | Yes |
| 1155 | TCGA-79-5596 | Lung Squamous Cell Carcinoma | Lung | Lung | Yes |
| 1156 | TCGA-EL-A3GS | Thyroid Carcinoma | Thymus | Thymus | Yes |
| 1157 | TCGA-W5-AA2Z | Cholangiocarcinoma | Colorectal | Colorectal | Yes |
| 1158 | TCGA-IE-A6BZ | Sarcoma | Skin | Skin | Yes |
| 1159 | TCGA-CN-A6V6 | Head and Neck Squamous Cell Carcinoma | Head and Neck | Head and Neck | Yes |
| 1160 | TCGA-KM-8442 | Kidney Chromophobe | Kidney | Kidney | Yes |
| 1161 | TCGA-A7-A3J0 | Breast Invasive Carcinoma | Breast | Breast | Yes |
| 1162 | TCGA-EM-A3AK | Thyroid Carcinoma | Thyroid | Thyroid | Yes |
| 1163 | TCGA-5L-AAT1 | Breast Invasive Carcinoma | Breast | Breast | Yes |
| 1164 | TCGA-UF-A7JK | Head and Neck Squamous Cell Carcinoma | Kidney | Kidney | Yes |
| 1165 | TCGA-FD-A3B6 | Bladder Urothelial Carcinoma | Bladder | Bladder | Yes |
| 1166 | TCGA-LN-A49X | Esophageal Carcinoma | Esophagus | Esophagus | Yes |
| 1167 | TCGA-D8-A1XD | Breast Invasive Carcinoma | Breast | Breast | Yes |
| 1168 | TCGA-CN-5359 | Head and Neck Squamous Cell Carcinoma | Head and Neck | Head and Neck | Yes |
| 1169 | TCGA-3B-A9HJ | Sarcoma | Soft Tissue | Soft Tissue | Yes |
| 1170 | TCGA-CF-A5UA | Bladder Urothelial Carcinoma | Bladder | Bladder | Yes |
| 1171 | TCGA-DH-A7UR | Brain Lower Grade Glioma | Brain | Brain | Yes |
| 1172 | TCGA-RL-AAAS | Pancreatic Adenocarcinoma | Adrenal Gland | Adrenal Gland | Yes |
| 1173 | TCGA-KK-A6E7 | Prostate Adenocarcinoma | Prostate | Prostate | Yes |
| 1174 | TCGA-B0-5115 | Kidney Renal Clear Cell Carcinoma | Kidney | Kidney | Yes |
| 1175 | TCGA-BR-8677 | Stomach Adenocarcinoma | Testis | Testis | Yes |
| 1176 | TCGA-77-A5FZ | Lung Squamous Cell Carcinoma | Lung | Lung | Yes |
| 1177 | TCGA-HC-7747 | Prostate Adenocarcinoma | Prostate | Prostate | Yes |
| 1178 | TCGA-DB-5277 | Brain Lower Grade Glioma | Brain | Brain | Yes |
| 1179 | TCGA-2F-A9KO | Bladder Urothelial Carcinoma | Bladder | Bladder | Yes |
| 1180 | TCGA-WB-A81R | Pheochromocytoma and Paraganglioma | Prostate | Prostate | Yes |
| 1181 | TCGA-69-7761 | Lung Adenocarcinoma | Lung | Lung | Yes |
| 1182 | TCGA-L5-A88Z | Esophageal Carcinoma | Brain | Brain | Yes |
| 1183 | TCGA-S9-A7J2 | Brain Lower Grade Glioma | Liver | Liver | Yes |
| 1184 | TCGA-44-6775 | Lung Adenocarcinoma | Lung | Lung | Yes |
| 1185 | TCGA-HC-8216 | Prostate Adenocarcinoma | Prostate | Prostate | Yes |
| 1186 | TCGA-KB-A93G | Stomach Adenocarcinoma | Stomach | Stomach | Yes |
| 1187 | TCGA-F2-A44H | Pancreatic Adenocarcinoma | Adrenal Gland | Adrenal Gland | Yes |
| 1188 | TCGA-2H-A9GQ | Esophageal Carcinoma | Brain | Brain | Yes |
| 1189 | TCGA-2G-AAFZ | Testicular Germ Cell Tumors | Thyroid | Thyroid | Yes |
| 1190 | TCGA-99-AA5R | Lung Adenocarcinoma | Lung | Lung | Yes |
| 1191 | TCGA-E1-A7YH | Brain Lower Grade Glioma | Brain | Brain | Yes |
| 1192 | TCGA-EJ-7331 | Prostate Adenocarcinoma | Prostate | Prostate | Yes |
| 1193 | TCGA-55-6972 | Lung Adenocarcinoma | Lung | Lung | Yes |
| 1194 | TCGA-HC-A8CY | Prostate Adenocarcinoma | Colorectal | Colorectal | Yes |
| 1195 | TCGA-E1-A7YI | Brain Lower Grade Glioma | Liver | Liver | Yes |
| 1196 | TCGA-ZH-A8Y1 | Cholangiocarcinoma | Colorectal | Colorectal | Yes |
| 1197 | TCGA-HI-7168 | Prostate Adenocarcinoma | Soft Tissue | Soft Tissue | Yes |
| 1198 | TCGA-E7-A678 | Bladder Urothelial Carcinoma | Bladder | Bladder | Yes |
| 1199 | TCGA-BH-A202 | Breast Invasive Carcinoma | Breast | Breast | Yes |
| 1200 | TCGA-FG-8191 | Brain Lower Grade Glioma | Brain | Brain | Yes |
| 1201 | TCGA-DK-A3IK | Bladder Urothelial Carcinoma | Bladder | Bladder | Yes |
| 1202 | TCGA-VR-A8Q7 | Esophageal Carcinoma | Brain | Brain | Yes |
| 1203 | TCGA-GU-AATQ | Bladder Urothelial Carcinoma | Bladder | Bladder | Yes |
| 1204 | TCGA-VQ-A8PC | Stomach Adenocarcinoma | Testis | Testis | Yes |
| 1205 | TCGA-HT-8564 | Brain Lower Grade Glioma | Brain | Brain | Yes |
| 1206 | TCGA-TS-A7OZ | Mesothelioma | Pancreas | Pancreas | Yes |
| 1207 | TCGA-LN-A9FP | Esophageal Carcinoma | Esophagus | Esophagus | Yes |
| 1208 | TCGA-44-7670 | Lung Adenocarcinoma | Lung | Lung | Yes |
| 1209 | TCGA-F5-6464 | Rectum Adenocarcinoma | Soft Tissue | Soft Tissue | Yes |
| 1210 | TCGA-P3-A5Q5 | Head and Neck Squamous Cell Carcinoma | Head and Neck | Head and Neck | Yes |
| 1211 | TCGA-BH-A0BM | Breast Invasive Carcinoma | Breast | Breast | Yes |
| 1212 | TCGA-CR-7372 | Head and Neck Squamous Cell Carcinoma | Head and Neck | Lung | No |
| 1213 | TCGA-DM-A1HB | Colon Adenocarcinoma | Colorectal | Colorectal | Yes |
| 1214 | TCGA-BR-8690 | Stomach Adenocarcinoma | Stomach | Stomach | Yes |
| 1215 | TCGA-VM-A8C8 | Brain Lower Grade Glioma | Brain | Brain | Yes |
| 1216 | TCGA-MM-A564 | Kidney Renal Clear Cell Carcinoma | Kidney | Kidney | Yes |
| 1217 | TCGA-2Z-A9JI | Kidney Renal Papillary Cell Carcinoma | Brain | Brain | Yes |
| 1218 | TCGA-KL-8325 | Kidney Chromophobe | Kidney | Kidney | Yes |
| 1219 | TCGA-UY-A8OB | Bladder Urothelial Carcinoma | Bladder | Bladder | Yes |
| 1220 | TCGA-GM-A2DH | Breast Invasive Carcinoma | Breast | Breast | Yes |
| 1221 | TCGA-DD-A73B | Liver Hepatocellular Carcinoma | Liver | Liver | Yes |
| 1222 | TCGA-RC-A7S9 | Liver Hepatocellular Carcinoma | Lung | Lung | Yes |
| 1223 | TCGA-AK-3453 | Kidney Renal Clear Cell Carcinoma | Kidney | Kidney | Yes |
| 1224 | TCGA-HC-7740 | Prostate Adenocarcinoma | Colorectal | Colorectal | Yes |
| 1225 | TCGA-HP-A5MZ | Liver Hepatocellular Carcinoma | Liver | Liver | Yes |
| 1226 | TCGA-KU-A6H8 | Head and Neck Squamous Cell Carcinoma | Head and Neck | Kidney | No |
| 1227 | TCGA-J9-A8CM | Prostate Adenocarcinoma | Soft Tissue | Soft Tissue | Yes |
| 1228 | TCGA-RM-A68W | Pheochromocytoma and Paraganglioma | Prostate | Prostate | Yes |
| 1229 | TCGA-WA-A7GZ | Head and Neck Squamous Cell Carcinoma | Head and Neck | Head and Neck | Yes |
| 1230 | TCGA-WB-A81P | Pheochromocytoma and Paraganglioma | Prostate | Prostate | Yes |
| 1231 | TCGA-G4-6626 | Colon Adenocarcinoma | Colorectal | Colorectal | Yes |
| 1232 | TCGA-50-5044 | Lung Adenocarcinoma | Lung | Lung | Yes |
| 1233 | TCGA-CV-5978 | Head and Neck Squamous Cell Carcinoma | Head and Neck | Head and Neck | Yes |
| 1234 | TCGA-3X-AAVE | Cholangiocarcinoma | Colorectal | Colorectal | Yes |
| 1235 | TCGA-CK-6748 | Colon Adenocarcinoma | Colorectal | Colorectal | Yes |
| 1236 | TCGA-AC-A2FM | Breast Invasive Carcinoma | Breast | Breast | Yes |
| 1237 | TCGA-T2-A6X0 | Head and Neck Squamous Cell Carcinoma | Head and Neck | Head and Neck | Yes |
| 1238 | TCGA-B0-4718 | Kidney Renal Clear Cell Carcinoma | Kidney | Kidney | Yes |
| 1239 | TCGA-A6-4107 | Colon Adenocarcinoma | Colorectal | Colorectal | Yes |
| 1240 | TCGA-IB-7647 | Pancreatic Adenocarcinoma | Adrenal Gland | Adrenal Gland | Yes |
| 1241 | TCGA-VQ-A8E7 | Stomach Adenocarcinoma | Stomach | Stomach | Yes |
| 1242 | TCGA-A2-A0CO | Breast Invasive Carcinoma | Breast | Breast | Yes |
| 1243 | TCGA-IQ-A61K | Head and Neck Squamous Cell Carcinoma | Head and Neck | Head and Neck | Yes |
| 1244 | TCGA-HD-8634 | Head and Neck Squamous Cell Carcinoma | Head and Neck | Esophagus | No |
| 1245 | TCGA-CV-7435 | Head and Neck Squamous Cell Carcinoma | Head and Neck | Head and Neck | Yes |
| 1246 | TCGA-3B-A9HQ | Sarcoma | Stomach | Stomach | Yes |
| 1247 | TCGA-RN-A68Q | Sarcoma | Skin | Skin | Yes |
| 1248 | TCGA-XF-A9SY | Bladder Urothelial Carcinoma | Bladder | Bladder | Yes |
| 1249 | TCGA-DH-A7UT | Brain Lower Grade Glioma | Liver | Liver | Yes |
| 1250 | TCGA-XF-A9SK | Bladder Urothelial Carcinoma | Bladder | Bladder | Yes |
| 1251 | TCGA-DO-A1K0 | Thyroid Carcinoma | Thyroid | Thyroid | Yes |
| 1252 | TCGA-EW-A1P0 | Breast Invasive Carcinoma | Breast | Breast | Yes |
| 1253 | TCGA-B6-A409 | Breast Invasive Carcinoma | Breast | Breast | Yes |
| 1254 | TCGA-CH-5789 | Prostate Adenocarcinoma | Prostate | Prostate | Yes |
| 1255 | TCGA-2G-AAM3 |  | Thyroid | Thyroid | Yes |
| 1256 | TCGA-GC-A3RB | Bladder Urothelial Carcinoma | Bladder | Bladder | Yes |
| 1257 | TCGA-BA-5153 | Head and Neck Squamous Cell Carcinoma | Head and Neck | Head and Neck | Yes |
| 1258 | TCGA-D7-8573 | Stomach Adenocarcinoma | Testis | Testis | Yes |
| 1259 | TCGA-EL-A3GQ | Thyroid Carcinoma | Thyroid | Thyroid | Yes |
| 1260 | TCGA-FP-7735 | Stomach Adenocarcinoma | Stomach | Stomach | Yes |
| 1261 | TCGA-55-8092 | Lung Adenocarcinoma | Lung | Lung | Yes |
| 1262 | TCGA-D7-A6EZ | Stomach Adenocarcinoma | Stomach | Stomach | Yes |
| 1263 | TCGA-OL-A5RX | Breast Invasive Carcinoma | Breast | Breast | Yes |
| 1264 | TCGA-EM-A2CS | Thyroid Carcinoma | Thyroid | Thyroid | Yes |
| 1265 | TCGA-CM-6675 | Colon Adenocarcinoma | Colorectal | Colorectal | Yes |
| 1266 | TCGA-85-8071 | Lung Squamous Cell Carcinoma | Lung | Lung | Yes |
| 1267 | TCGA-97-A4M3 | Lung Adenocarcinoma | Lung | Lung | Yes |
| 1268 | TCGA-A6-5656 | Colon Adenocarcinoma | Colorectal | Colorectal | Yes |
| 1269 | TCGA-HZ-8003 | Pancreatic Adenocarcinoma | Adrenal Gland | Adrenal Gland | Yes |
| 1270 | TCGA-06-A7TK | Glioblastoma Multiforme | Brain | Brain | Yes |
| 1271 | TCGA-HU-A4G8 | Stomach Adenocarcinoma | Testis | Testis | Yes |
| 1272 | TCGA-XF-AAMJ | Bladder Urothelial Carcinoma | Bladder | Bladder | Yes |
| 1273 | TCGA-33-A4WN | Lung Squamous Cell Carcinoma | Pancreas | Pancreas | Yes |
| 1274 | TCGA-YA-A8S7 | Liver Hepatocellular Carcinoma | Liver | Liver | Yes |
| 1275 | TCGA-A3-3385 | Kidney Renal Clear Cell Carcinoma | Kidney | Kidney | Yes |
| 1276 | TCGA-SC-A6LN | Mesothelioma | Pancreas | Pancreas | Yes |
| 1277 | TCGA-CG-5727 | Stomach Adenocarcinoma | Stomach | Stomach | Yes |
| 1278 | TCGA-3A-A9IX | Pancreatic Adenocarcinoma | Adrenal Gland | Adrenal Gland | Yes |
| 1279 | TCGA-38-4632 | Lung Adenocarcinoma | Lung | Lung | Yes |
| 1280 | TCGA-44-5644 | Lung Adenocarcinoma | Lung | Lung | Yes |
| 1281 | TCGA-OR-A5L3 | Adrenocortical Carcinoma | Adrenal Gland | Adrenal Gland | Yes |
| 1282 | TCGA-B4-5832 | Kidney Renal Clear Cell Carcinoma | Kidney | Kidney | Yes |
| 1283 | TCGA-DE-A3KN | Thyroid Carcinoma | Thymus | Thymus | Yes |
| 1284 | TCGA-A3-A8OV | Kidney Renal Clear Cell Carcinoma | Kidney | Kidney | Yes |
| 1285 | TCGA-BR-A4J5 | Stomach Adenocarcinoma | Testis | Testis | Yes |
| 1286 | TCGA-G3-A25S | Liver Hepatocellular Carcinoma | Liver | Liver | Yes |
| 1287 | TCGA-S9-A7R4 | Brain Lower Grade Glioma | Brain | Brain | Yes |
| 1288 | TCGA-95-8039 | Lung Adenocarcinoma | Lung | Lung | Yes |
| 1289 | TCGA-CK-4952 | Colon Adenocarcinoma | Esophagus | Stomach | No |
| 1290 | TCGA-91-6848 | Lung Adenocarcinoma | Lung | Lung | Yes |
| 1291 | TCGA-O2-A52V | Lung Squamous Cell Carcinoma | Lung | Head and Neck | No |
| 1292 | TCGA-SX-A7SL | Kidney Renal Papillary Cell Carcinoma | Kidney | Kidney | Yes |
| 1293 | TCGA-EM-A2CP | Thyroid Carcinoma | Thyroid | Thyroid | Yes |
| 1294 | TCGA-ZP-A9D4 | Liver Hepatocellular Carcinoma | Liver | Liver | Yes |
| 1295 | TCGA-2F-A9KR | Bladder Urothelial Carcinoma | Bladder | Bladder | Yes |
| 1296 | TCGA-TM-A84C | Brain Lower Grade Glioma | Brain | Brain | Yes |
| 1297 | TCGA-FA-8693 | Lymphoid Neoplasm Diffuse Large B-cell Lymphoma | Esophagus | Esophagus | Yes |
| 1298 | TCGA-L3-A4E7 | Lung Squamous Cell Carcinoma | Lung | Lung | Yes |
| 1299 | TCGA-BR-7959 | Stomach Adenocarcinoma | Stomach | Stomach | Yes |
| 1300 | TCGA-78-7633 | Lung Adenocarcinoma | Lung | Lung | Yes |
| 1301 | TCGA-A2-A04R | Breast Invasive Carcinoma | Breast | Breast | Yes |
| 1302 | TCGA-R5-A804 | Stomach Adenocarcinoma | Testis | Testis | Yes |
| 1303 | TCGA-64-5775 | Lung Adenocarcinoma | Lung | Lung | Yes |
| 1304 | TCGA-DX-A7EO | Sarcoma | Soft Tissue | Soft Tissue | Yes |
| 1305 | TCGA-HU-8244 | Stomach Adenocarcinoma | Thyroid | Thyroid | Yes |
| 1306 | TCGA-DJ-A2Q9 | Thyroid Carcinoma | Thyroid | Thyroid | Yes |
| 1307 | TCGA-VQ-AA64 | Stomach Adenocarcinoma | Stomach | Stomach | Yes |
| 1308 | TCGA-G7-7502 | Kidney Renal Papillary Cell Carcinoma | Brain | Brain | Yes |
| 1309 | TCGA-OR-A5JP | Adrenocortical Carcinoma | Adrenal Gland | Adrenal Gland | Yes |
| 1310 | TCGA-CI-6620 | Rectum Adenocarcinoma | Soft Tissue | Soft Tissue | Yes |
| 1311 | TCGA-MQ-A4LM | Mesothelioma | Pancreas | Pancreas | Yes |
| 1312 | TCGA-78-7152 | Lung Adenocarcinoma | Lung | Breast | No |
| 1313 | TCGA-WB-A81A | Pheochromocytoma and Paraganglioma | Prostate | Prostate | Yes |
| 1314 | TCGA-CR-7370 | Head and Neck Squamous Cell Carcinoma | Head and Neck | Head and Neck | Yes |
| 1315 | TCGA-CC-A7IH | Liver Hepatocellular Carcinoma | Liver | Liver | Yes |
| 1316 | TCGA-CJ-5678 | Kidney Renal Clear Cell Carcinoma | Kidney | Kidney | Yes |
| 1317 | TCGA-22-4613 | Lung Squamous Cell Carcinoma | Lung | Lung | Yes |
| 1318 | TCGA-ET-A2N1 | Thyroid Carcinoma | Thymus | Thymus | Yes |
| 1319 | TCGA-B0-5698 | Kidney Renal Clear Cell Carcinoma | Kidney | Kidney | Yes |
| 1320 | TCGA-91-A4BD | Lung Adenocarcinoma | Lung | Lung | Yes |
| 1321 | TCGA-HT-7602 | Brain Lower Grade Glioma | Brain | Brain | Yes |
| 1322 | TCGA-19-0957 | Glioblastoma Multiforme | Brain | Brain | Yes |
| 1323 | TCGA-44-8117 | Lung Adenocarcinoma | Lung | Lung | Yes |
| 1324 | TCGA-EM-A2OV | Thyroid Carcinoma | Thyroid | Thyroid | Yes |
| 1325 | TCGA-44-A47B | Lung Adenocarcinoma | Lung | Lung | Yes |
| 1326 | TCGA-CM-5348 | Colon Adenocarcinoma | Esophagus | Head and Neck | No |
| 1327 | TCGA-EB-A1NK | Skin Cutaneous Melanoma | Stomach | Stomach | Yes |
| 1328 | TCGA-FT-A61P | Bladder Urothelial Carcinoma | Bladder | Bladder | Yes |
| 1329 | TCGA-LN-A7HZ | Esophageal Carcinoma | Brain | Brain | Yes |
| 1330 | TCGA-50-5936 | Lung Adenocarcinoma | Lung | Lung | Yes |
| 1331 | TCGA-AZ-6605 | Colon Adenocarcinoma | Colorectal | Colorectal | Yes |
| 1332 | TCGA-DM-A1DB | Colon Adenocarcinoma | Colorectal | Colorectal | Yes |
| 1333 | TCGA-S7-A7WX | Pheochromocytoma and Paraganglioma | Prostate | Prostate | Yes |
| 1334 | TCGA-S9-A6TX | Brain Lower Grade Glioma | Brain | Brain | Yes |
| 1335 | TCGA-94-7557 | Lung Squamous Cell Carcinoma | Lung | Lung | Yes |
| 1336 | TCGA-O2-A5IB | Lung Squamous Cell Carcinoma | Pancreas | Pancreas | Yes |
| 1337 | TCGA-29-A5NZ | Ovarian Serous Cystadenocarcinoma | Adrenal Gland | Adrenal Gland | Yes |
| 1338 | TCGA-EL-A3H8 | Thyroid Carcinoma | Thyroid | Thyroid | Yes |
| 1339 | TCGA-HC-7210 | Prostate Adenocarcinoma | Colorectal | Colorectal | Yes |
| 1340 | TCGA-CV-7099 | Head and Neck Squamous Cell Carcinoma | Kidney | Kidney | Yes |
| 1341 | TCGA-FD-A6TB | Bladder Urothelial Carcinoma | Bladder | Bladder | Yes |
| 1342 | TCGA-EP-A2KC | Liver Hepatocellular Carcinoma | Liver | Liver | Yes |
| 1343 | TCGA-DK-AA77 | Bladder Urothelial Carcinoma | Bladder | Bladder | Yes |
| 1344 | TCGA-WK-A8XS | Sarcoma | Soft Tissue | Soft Tissue | Yes |
| 1345 | TCGA-DD-A3A3 | Liver Hepatocellular Carcinoma | Lung | Lung | Yes |
| 1346 | TCGA-MH-A857 | Kidney Renal Papillary Cell Carcinoma | Kidney | Kidney | Yes |
| 1347 | TCGA-P5-A77X | Brain Lower Grade Glioma | Brain | Brain | Yes |
| 1348 | TCGA-2G-AAH8 | Testicular Germ Cell Tumors | Thyroid | Thyroid | Yes |
| 1349 | TCGA-NJ-A55A | Lung Adenocarcinoma | Lung | Lung | Yes |
| 1350 | TCGA-AM-5821 | Colon Adenocarcinoma | Colorectal | Colorectal | Yes |
| 1351 | TCGA-RD-A7BW | Stomach Adenocarcinoma | Stomach | Stomach | Yes |
| 1352 | TCGA-BH-A0DD | Breast Invasive Carcinoma | Breast | Breast | Yes |
| 1353 | TCGA-38-A44F | Lung Adenocarcinoma | Lung | Lung | Yes |
| 1354 | TCGA-CH-5766 | Prostate Adenocarcinoma | Prostate | Prostate | Yes |
| 1355 | TCGA-S9-A7R7 | Brain Lower Grade Glioma | Brain | Brain | Yes |
| 1356 | TCGA-C8-A26W | Breast Invasive Carcinoma | Breast | Breast | Yes |
| 1357 | TCGA-DZ-6132 | Kidney Renal Papillary Cell Carcinoma | Kidney | Kidney | Yes |
| 1358 | TCGA-DX-A3LT | Sarcoma | Soft Tissue | Soft Tissue | Yes |
| 1359 | TCGA-EJ-5505 | Prostate Adenocarcinoma | Prostate | Prostate | Yes |
| 1360 | TCGA-XV-A9VZ | Skin Cutaneous Melanoma | Stomach | Stomach | Yes |
| 1361 | TCGA-EJ-7321 | Prostate Adenocarcinoma | Colorectal | Colorectal | Yes |
| 1362 | TCGA-BJ-A291 | Thyroid Carcinoma | Thymus | Thymus | Yes |
| 1363 | TCGA-05-5429 | Lung Adenocarcinoma | Lung | Lung | Yes |
| 1364 | TCGA-A4-A772 | Kidney Renal Papillary Cell Carcinoma | Kidney | Kidney | Yes |
| 1365 | TCGA-AA-3502 | Colon Adenocarcinoma | Colorectal | Colorectal | Yes |
| 1366 | TCGA-ZG-A9LM | Prostate Adenocarcinoma | Prostate | Prostate | Yes |
| 1367 | TCGA-RY-A83Y | Brain Lower Grade Glioma | Brain | Brain | Yes |
| 1368 | TCGA-HT-7467 | Brain Lower Grade Glioma | Brain | Brain | Yes |
| 1369 | TCGA-CD-A4MG | Stomach Adenocarcinoma | Stomach | Stomach | Yes |
| 1370 | TCGA-IW-A3M6 | Sarcoma | Skin | Skin | Yes |
| 1371 | TCGA-14-1043 | Glioblastoma Multiforme | Head and Neck | Esophagus | No |
| 1372 | TCGA-2Y-A9GS | Liver Hepatocellular Carcinoma | Liver | Liver | Yes |
| 1373 | TCGA-ZF-AA5H | Bladder Urothelial Carcinoma | Bladder | Bladder | Yes |
| 1374 | TCGA-EL-A3ZR | Thyroid Carcinoma | Thyroid | Thyroid | Yes |
| 1375 | TCGA-43-A56V | Lung Squamous Cell Carcinoma | Lung | Lung | Yes |
| 1376 | TCGA-DU-6394 | Brain Lower Grade Glioma | Brain | Brain | Yes |
| 1377 | TCGA-55-7574 | Lung Adenocarcinoma | Lung | Lung | Yes |
| 1378 | TCGA-ET-A3DS | Thyroid Carcinoma | Thyroid | Thyroid | Yes |
| 1379 | TCGA-BC-A10W | Liver Hepatocellular Carcinoma | Lung | Lung | Yes |
| 1380 | TCGA-BJ-A190 | Thyroid Carcinoma | Thymus | Thymus | Yes |
| 1381 | TCGA-85-6798 | Lung Squamous Cell Carcinoma | Lung | Esophagus | No |
| 1382 | TCGA-T3-A92M | Head and Neck Squamous Cell Carcinoma | Head and Neck | Head and Neck | Yes |
| 1383 | TCGA-CN-4735 | Head and Neck Squamous Cell Carcinoma | Kidney | Kidney | Yes |
| 1384 | TCGA-AD-6963 | Colon Adenocarcinoma | Colorectal | Colorectal | Yes |
| 1385 | TCGA-EJ-8472 | Prostate Adenocarcinoma | Prostate | Prostate | Yes |
| 1386 | TCGA-ZM-AA0H | Testicular Germ Cell Tumors | Thyroid | Thyroid | Yes |
| 1387 | TCGA-55-7726 | Lung Adenocarcinoma | Lung | Lung | Yes |
| 1388 | TCGA-E8-A414 | Thyroid Carcinoma | Thymus | Breast | No |
| 1389 | TCGA-44-A4SU | Lung Adenocarcinoma | Lung | Lung | Yes |
| 1390 | TCGA-G9-6378 | Prostate Adenocarcinoma | Soft Tissue | Soft Tissue | Yes |
| 1391 | TCGA-EM-A2CM | Thyroid Carcinoma | Thymus | Thymus | Yes |
| 1392 | TCGA-BP-5202 | Kidney Renal Clear Cell Carcinoma | Kidney | Kidney | Yes |
| 1393 | TCGA-DX-A8BT | Sarcoma | Skin | Skin | Yes |
| 1394 | TCGA-E9-A6HE | Breast Invasive Carcinoma | Breast | Breast | Yes |
| 1395 | TCGA-HD-8314 | Head and Neck Squamous Cell Carcinoma | Head and Neck | Head and Neck | Yes |
| 1396 | TCGA-DE-A4MB | Thyroid Carcinoma | Thyroid | Thyroid | Yes |
| 1397 | TCGA-CI-6623 | Rectum Adenocarcinoma | Soft Tissue | Soft Tissue | Yes |
| 1398 | TCGA-Z5-AAPL | Pancreatic Adenocarcinoma | Adrenal Gland | Adrenal Gland | Yes |
| 1399 | TCGA-G4-6322 | Colon Adenocarcinoma | Colorectal | Colorectal | Yes |
| 1400 | TCGA-A7-A3RF | Breast Invasive Carcinoma | Breast | Breast | Yes |
| 1401 | TCGA-S7-A7WQ | Pheochromocytoma and Paraganglioma | Prostate | Prostate | Yes |
| 1402 | TCGA-55-8513 | Lung Adenocarcinoma | Lung | Lung | Yes |
| 1403 | TCGA-SN-A84Y | Testicular Germ Cell Tumors | Thyroid | Thyroid | Yes |
| 1404 | TCGA-HW-7489 | Brain Lower Grade Glioma | Brain | Brain | Yes |
| 1405 | TCGA-FD-A6TA | Bladder Urothelial Carcinoma | Bladder | Bladder | Yes |
| 1406 | TCGA-CN-6018 | Head and Neck Squamous Cell Carcinoma | Head and Neck | Head and Neck | Yes |
| 1407 | TCGA-BW-A5NP | Liver Hepatocellular Carcinoma | Liver | Liver | Yes |
| 1408 | TCGA-DJ-A2Q2 | Thyroid Carcinoma | Thymus | Thymus | Yes |
| 1409 | TCGA-JY-A938 | Esophageal Carcinoma | Brain | Brain | Yes |
| 1410 | TCGA-VN-A88K | Prostate Adenocarcinoma | Prostate | Prostate | Yes |
| 1411 | TCGA-76-6663 | Glioblastoma Multiforme | Brain | Brain | Yes |
| 1412 | TCGA-DV-5575 | Kidney Renal Clear Cell Carcinoma | Kidney | Kidney | Yes |
| 1413 | TCGA-06-A5U0 | Glioblastoma Multiforme | Brain | Brain | Yes |
| 1414 | TCGA-05-4405 | Lung Adenocarcinoma | Lung | Lung | Yes |
| 1415 | TCGA-MI-A75E | Liver Hepatocellular Carcinoma | Lung | Lung | Yes |
| 1416 | TCGA-DU-5870 | Brain Lower Grade Glioma | Brain | Brain | Yes |
| 1417 | TCGA-EL-A3CP | Thyroid Carcinoma | Thymus | Thymus | Yes |
| 1418 | TCGA-L5-A4ON | Esophageal Carcinoma | Brain | Brain | Yes |
| 1419 | TCGA-80-5607 | Lung Adenocarcinoma | Lung | Lung | Yes |
| 1420 | TCGA-UY-A78O | Bladder Urothelial Carcinoma | Bladder | Bladder | Yes |
| 1421 | TCGA-DC-6160 | Rectum Adenocarcinoma | Soft Tissue | Soft Tissue | Yes |
| 1422 | TCGA-HT-7482 | Brain Lower Grade Glioma | Brain | Brain | Yes |
| 1423 | TCGA-2H-A9GR | Esophageal Carcinoma | Esophagus | Esophagus | Yes |
| 1424 | TCGA-VN-A88N | Prostate Adenocarcinoma | Prostate | Prostate | Yes |
| 1425 | TCGA-EV-5902 | Kidney Renal Papillary Cell Carcinoma | Brain | Brain | Yes |
| 1426 | TCGA-EL-A3ZL | Thyroid Carcinoma | Thymus | Thymus | Yes |
| 1427 | TCGA-OR-A5J2 | Adrenocortical Carcinoma | Adrenal Gland | Adrenal Gland | Yes |
| 1428 | TCGA-50-8460 | Lung Adenocarcinoma | Lung | Lung | Yes |
| 1429 | TCGA-QR-A70N | Pheochromocytoma and Paraganglioma | Prostate | Prostate | Yes |
| 1430 | TCGA-CF-A3MG | Bladder Urothelial Carcinoma | Bladder | Bladder | Yes |
| 1431 | TCGA-DK-AA76 | Bladder Urothelial Carcinoma | Bladder | Bladder | Yes |
| 1432 | TCGA-DK-A1A6 | Bladder Urothelial Carcinoma | Bladder | Bladder | Yes |
| 1433 | TCGA-KN-8423 | Kidney Chromophobe | Kidney | Kidney | Yes |
| 1434 | TCGA-AN-A0XR | Breast Invasive Carcinoma | Breast | Breast | Yes |
| 1435 | TCGA-O2-A52W | Lung Squamous Cell Carcinoma | Lung | Lung | Yes |
| 1436 | TCGA-19-5955 | Glioblastoma Multiforme | Brain | Brain | Yes |
| 1437 | TCGA-IN-A7NU | Stomach Adenocarcinoma | Stomach | Stomach | Yes |
| 1438 | TCGA-EI-7004 | Rectum Adenocarcinoma | Soft Tissue | Soft Tissue | Yes |
| 1439 | TCGA-T2-A6WX | Head and Neck Squamous Cell Carcinoma | Head and Neck | Head and Neck | Yes |
| 1440 | TCGA-FG-8188 | Brain Lower Grade Glioma | Liver | Liver | Yes |
| 1441 | TCGA-G9-6336 | Prostate Adenocarcinoma | Prostate | Prostate | Yes |
| 1442 | TCGA-OR-A5KY | Adrenocortical Carcinoma | Adrenal Gland | Adrenal Gland | Yes |
| 1443 | TCGA-BF-A1PU | Skin Cutaneous Melanoma | Stomach | Stomach | Yes |
| 1444 | TCGA-DX-A7ER | Sarcoma | Stomach | Stomach | Yes |
| 1445 | TCGA-TS-A7P6 | Mesothelioma | Pancreas | Pancreas | Yes |
| 1446 | TCGA-DU-6403 | Brain Lower Grade Glioma | Brain | Brain | Yes |
| 1447 | TCGA-4A-A93W | Kidney Renal Papillary Cell Carcinoma | Brain | Brain | Yes |
| 1448 | TCGA-XY-A89B | Testicular Germ Cell Tumors | Thyroid | Thyroid | Yes |
| 1449 | TCGA-EL-A4KH | Thyroid Carcinoma | Thymus | Thymus | Yes |
| 1450 | TCGA-BT-A20N | Bladder Urothelial Carcinoma | Bladder | Bladder | Yes |
| 1451 | TCGA-DD-AAE3 | Liver Hepatocellular Carcinoma | Liver | Liver | Yes |
| 1452 | TCGA-A1-A0SG | Breast Invasive Carcinoma | Breast | Breast | Yes |
| 1453 | TCGA-2H-A9GF | Esophageal Carcinoma | Esophagus | Esophagus | Yes |
| 1454 | TCGA-B7-A5TJ | Stomach Adenocarcinoma | Stomach | Stomach | Yes |
| 1455 | TCGA-BQ-7050 | Kidney Renal Papillary Cell Carcinoma | Kidney | Kidney | Yes |
| 1456 | TCGA-RW-A68B | Pheochromocytoma and Paraganglioma | Prostate | Prostate | Yes |
| 1457 | TCGA-2Y-A9GV | Liver Hepatocellular Carcinoma | Liver | Liver | Yes |
| 1458 | TCGA-P5-A77W | Brain Lower Grade Glioma | Liver | Liver | Yes |
| 1459 | TCGA-DX-A3UF | Sarcoma | Stomach | Stomach | Yes |
| 1460 | TCGA-DD-A73C | Liver Hepatocellular Carcinoma | Lung | Lung | Yes |
| 1461 | TCGA-LL-A7SZ | Breast Invasive Carcinoma | Breast | Breast | Yes |
| 1462 | TCGA-CN-4723 | Head and Neck Squamous Cell Carcinoma | Head and Neck | Head and Neck | Yes |
| 1463 | TCGA-AC-A2QH | Breast Invasive Carcinoma | Breast | Breast | Yes |
| 1464 | TCGA-DX-AB30 | Sarcoma | Stomach | Stomach | Yes |
| 1465 | TCGA-BR-6710 | Stomach Adenocarcinoma | Stomach | Stomach | Yes |
| 1466 | TCGA-E8-A2EA | Thyroid Carcinoma | Thyroid | Thyroid | Yes |
| 1467 | TCGA-33-4582 | Lung Squamous Cell Carcinoma | Lung | Lung | Yes |
| 1468 | TCGA-BH-A42V | Breast Invasive Carcinoma | Breast | Breast | Yes |
