# Supplementary material for: Predicting cancer origins with a DNA methylation-based deep neural network model
Source: PLoS One. 2020 May 8;15(5):e0226461. doi: 10.1371/journal.pone.0226461 (PMC7209244; doi:10.1371/journal.pone.0226461)
Supplement: S3 Table — (DOCX) [file pone.0226461.s003.docx]

**S3 Table. Cancer tissue origin predictions for 143 metastatic cancer samples.**

|  | **Patient** | **Diagnosis** | **Primary_site** | **Prediction** | **Correct** |
| --- | --- | --- | --- | --- | --- |
| 1 | TCGA-BP-5178 | Kidney Renal Clear Cell Carcinoma | Kidney | Kidney | Yes |
| 2 | TCGA-CV-7252 | Head and Neck Squamous Cell Carcinoma | Head and Neck | Head and Neck | Yes |
| 3 | TCGA-CU-A72E | Bladder Urothelial Carcinoma | Bladder | Bladder | Yes |
| 4 | TCGA-HD-A634 | Head and Neck Squamous Cell Carcinoma | Head and Neck | Esophagus | No |
| 5 | TCGA-CN-5366 | Head and Neck Squamous Cell Carcinoma | Head and Neck | Head and Neck | Yes |
| 6 | TCGA-OR-A5J4 | Adrenocortical Carcinoma | Adrenal Gland | Adrenal Gland | Yes |
| 7 | TCGA-HD-7754 | Head and Neck Squamous Cell Carcinoma | Head and Neck | Head and Neck | Yes |
| 8 | TCGA-HD-7831 | Head and Neck Squamous Cell Carcinoma | Head and Neck | Head and Neck | Yes |
| 9 | TCGA-R5-A7O7 | Stomach Adenocarcinoma | Stomach | Stomach | Yes |
| 10 | TCGA-LN-A5U5 | Esophageal Carcinoma | Esophagus | Esophagus | Yes |
| 11 | TCGA-CR-7386 | Head and Neck Squamous Cell Carcinoma | Head and Neck | Head and Neck | Yes |
| 12 | TCGA-CN-4739 | Head and Neck Squamous Cell Carcinoma | Head and Neck | Head and Neck | Yes |
| 13 | TCGA-BT-A20T | Bladder Urothelial Carcinoma | Bladder | Bladder | Yes |
| 14 | TCGA-CV-7236 | Head and Neck Squamous Cell Carcinoma | Head and Neck | Head and Neck | Yes |
| 15 | TCGA-H7-A6C4 | Head and Neck Squamous Cell Carcinoma | Head and Neck | Head and Neck | Yes |
| 16 | TCGA-B0-5712 | Kidney Renal Clear Cell Carcinoma | Kidney | Kidney | Yes |
| 17 | TCGA-CN-4726 | Head and Neck Squamous Cell Carcinoma | Head and Neck | Head and Neck | Yes |
| 18 | TCGA-BW-A5NP | Liver Hepatocellular Carcinoma | Liver | Liver | Yes |
| 19 | TCGA-55-8620 | Lung Adenocarcinoma | Lung | Liver | No |
| 20 | TCGA-CQ-5334 | Head and Neck Squamous Cell Carcinoma | Head and Neck | Head and Neck | Yes |
| 21 | TCGA-B0-5080 | Kidney Renal Clear Cell Carcinoma | Kidney | Kidney | Yes |
| 22 | TCGA-CN-6992 | Head and Neck Squamous Cell Carcinoma | Head and Neck | Head and Neck | Yes |
| 23 | TCGA-ZF-AA5P | Bladder Urothelial Carcinoma | Bladder | Bladder | Yes |
| 24 | TCGA-A4-A57E | Kidney Renal Papillary Cell Carcinoma | Kidney | Kidney | Yes |
| 25 | TCGA-G4-6315 | Colon Adenocarcinoma | Colorectal | Colorectal | Yes |
| 26 | TCGA-D6-A6EQ | Head and Neck Squamous Cell Carcinoma | Head and Neck | Head and Neck | Yes |
| 27 | TCGA-AA-3662 | Colon Adenocarcinoma | Colorectal | Colorectal | Yes |
| 28 | TCGA-CN-5367 | Head and Neck Squamous Cell Carcinoma | Head and Neck | Head and Neck | Yes |
| 29 | TCGA-CU-A0YR | Bladder Urothelial Carcinoma | Bladder | Bladder | Yes |
| 30 | TCGA-ZF-AA4T | Bladder Urothelial Carcinoma | Bladder | Bladder | Yes |
| 31 | TCGA-ZF-AA58 | Bladder Urothelial Carcinoma | Bladder | Bladder | Yes |
| 32 | TCGA-CV-7103 | Head and Neck Squamous Cell Carcinoma | Head and Neck | Head and Neck | Yes |
| 33 | TCGA-VR-AA7B | Esophageal Carcinoma | Esophagus | Esophagus | Yes |
| 34 | TCGA-KU-A6H8 | Head and Neck Squamous Cell Carcinoma | Head and Neck | Head and Neck | Yes |
| 35 | TCGA-BJ-A3EZ | Thyroid Carcinoma | Thyroid | Thyroid | Yes |
| 36 | TCGA-CJ-5678 | Kidney Renal Clear Cell Carcinoma | Kidney | Kidney | Yes |
| 37 | TCGA-XF-A9T5 | Bladder Urothelial Carcinoma | Bladder | Esophagus | No |
| 38 | TCGA-XF-A9SJ | Bladder Urothelial Carcinoma | Bladder | Bladder | Yes |
| 39 | TCGA-XF-AAN7 | Bladder Urothelial Carcinoma | Bladder | Bladder | Yes |
| 40 | TCGA-CV-5431 | Head and Neck Squamous Cell Carcinoma | Head and Neck | Head and Neck | Yes |
| 41 | TCGA-BA-5555 | Head and Neck Squamous Cell Carcinoma | Head and Neck | Head and Neck | Yes |
| 42 | TCGA-CN-4723 | Head and Neck Squamous Cell Carcinoma | Head and Neck | Head and Neck | Yes |
| 43 | TCGA-B0-4844 | Kidney Renal Clear Cell Carcinoma | Kidney | Kidney | Yes |
| 44 | TCGA-CQ-6228 | Head and Neck Squamous Cell Carcinoma | Head and Neck | Head and Neck | Yes |
| 45 | TCGA-CN-5361 | Head and Neck Squamous Cell Carcinoma | Head and Neck | Esophagus | No |
| 46 | TCGA-DK-A3IL | Bladder Urothelial Carcinoma | Bladder | Bladder | Yes |
| 47 | TCGA-UF-A71D | Head and Neck Squamous Cell Carcinoma | Head and Neck | Head and Neck | Yes |
| 48 | TCGA-FD-A3SM | Bladder Urothelial Carcinoma | Bladder | Bladder | Yes |
| 49 | TCGA-H7-8502 | Head and Neck Squamous Cell Carcinoma | Head and Neck | Head and Neck | Yes |
| 50 | TCGA-CR-7397 | Head and Neck Squamous Cell Carcinoma | Head and Neck | Head and Neck | Yes |
| 51 | TCGA-UY-A78M | Bladder Urothelial Carcinoma | Bladder | Bladder | Yes |
| 52 | TCGA-UF-A7JA | Head and Neck Squamous Cell Carcinoma | Head and Neck | Head and Neck | Yes |
| 53 | TCGA-FE-A231 | Thyroid Carcinoma | Thyroid | Thyroid | Yes |
| 54 | TCGA-DC-6154 | Rectum Adenocarcinoma | Colorectal | Colorectal | Yes |
| 55 | TCGA-OR-A5KO | Adrenocortical Carcinoma | Adrenal Gland | Adrenal Gland | Yes |
| 56 | TCGA-BR-7957 | Stomach Adenocarcinoma | Stomach | Stomach | Yes |
| 57 | TCGA-78-7145 | Lung Adenocarcinoma | Lung | Lung | Yes |
| 58 | TCGA-BJ-A0ZH | Thyroid Carcinoma | Thyroid | Thyroid | Yes |
| 59 | TCGA-CV-5441 | Head and Neck Squamous Cell Carcinoma | Head and Neck | Head and Neck | Yes |
| 60 | TCGA-CR-7382 | Head and Neck Squamous Cell Carcinoma | Head and Neck | Head and Neck | Yes |
| 61 | TCGA-T3-A92M | Head and Neck Squamous Cell Carcinoma | Head and Neck | Esophagus | No |
| 62 | TCGA-CV-6962 | Head and Neck Squamous Cell Carcinoma | Head and Neck | Head and Neck | Yes |
| 63 | TCGA-CN-6016 | Head and Neck Squamous Cell Carcinoma | Head and Neck | Head and Neck | Yes |
| 64 | TCGA-CN-6010 | Head and Neck Squamous Cell Carcinoma | Head and Neck | Head and Neck | Yes |
| 65 | TCGA-CV-7089 | Head and Neck Squamous Cell Carcinoma | Head and Neck | Head and Neck | Yes |
| 66 | TCGA-UF-A7JK | Head and Neck Squamous Cell Carcinoma | Head and Neck | Head and Neck | Yes |
| 67 | TCGA-RC-A6M5 | Liver Hepatocellular Carcinoma | Liver | Liver | Yes |
| 68 | TCGA-FD-A3SQ | Bladder Urothelial Carcinoma | Bladder | Bladder | Yes |
| 69 | TCGA-BL-A13J | Bladder Urothelial Carcinoma | Bladder | Bladder | Yes |
| 70 | TCGA-EM-A2OX | Thyroid Carcinoma | Thyroid | Thyroid | Yes |
| 71 | TCGA-CQ-5326 | Head and Neck Squamous Cell Carcinoma | Head and Neck | Head and Neck | Yes |
| 72 | TCGA-IB-7644 | Pancreatic Adenocarcinoma | Pancreas | Pancreas | Yes |
| 73 | TCGA-SX-A7SM | Kidney Renal Papillary Cell Carcinoma | Kidney | Kidney | Yes |
| 74 | TCGA-KL-8339 | Kidney Chromophobe | Kidney | Kidney | Yes |
| 75 | TCGA-UF-A7JT | Head and Neck Squamous Cell Carcinoma | Head and Neck | Head and Neck | Yes |
| 76 | TCGA-VQ-A8PP | Stomach Adenocarcinoma | Stomach | Stomach | Yes |
| 77 | TCGA-CN-4728 | Head and Neck Squamous Cell Carcinoma | Head and Neck | Head and Neck | Yes |
| 78 | TCGA-FD-A3SJ | Bladder Urothelial Carcinoma | Bladder | Bladder | Yes |
| 79 | TCGA-BR-7196 | Stomach Adenocarcinoma | Stomach | Stomach | Yes |
| 80 | TCGA-CN-5365 | Head and Neck Squamous Cell Carcinoma | Head and Neck | Head and Neck | Yes |
| 81 | TCGA-EL-A3H5 | Thyroid Carcinoma | Thyroid | Thyroid | Yes |
| 82 | TCGA-P3-A6T0 | Head and Neck Squamous Cell Carcinoma | Head and Neck | Head and Neck | Yes |
| 83 | TCGA-DM-A285 | Colon Adenocarcinoma | Colorectal | Colorectal | Yes |
| 84 | TCGA-CV-7104 | Head and Neck Squamous Cell Carcinoma | Head and Neck | Head and Neck | Yes |
| 85 | TCGA-A2-A0SV | Breast Invasive Carcinoma | Breast | Breast | Yes |
| 86 | TCGA-CR-6471 | Head and Neck Squamous Cell Carcinoma | Head and Neck | Head and Neck | Yes |
| 87 | TCGA-CV-7409 | Head and Neck Squamous Cell Carcinoma | Head and Neck | Head and Neck | Yes |
| 88 | TCGA-EL-A3D4 | Thyroid Carcinoma | Thyroid | Thyroid | Yes |
| 89 | TCGA-5M-AAT6 | Colon Adenocarcinoma | Colorectal | Colorectal | Yes |
| 90 | TCGA-B0-4845 | Kidney Renal Clear Cell Carcinoma | Kidney | Kidney | Yes |
| 91 | TCGA-CN-A640 | Head and Neck Squamous Cell Carcinoma | Head and Neck | Head and Neck | Yes |
| 92 | TCGA-CV-6954 | Head and Neck Squamous Cell Carcinoma | Head and Neck | Head and Neck | Yes |
| 93 | TCGA-E8-A436 | Thyroid Carcinoma | Thyroid | Thyroid | Yes |
| 94 | TCGA-MT-A7BN | Head and Neck Squamous Cell Carcinoma | Head and Neck | Breast | No |
| 95 | TCGA-B0-4847 | Kidney Renal Clear Cell Carcinoma | Kidney | Kidney | Yes |
| 96 | TCGA-CV-6939 | Head and Neck Squamous Cell Carcinoma | Head and Neck | Head and Neck | Yes |
| 97 | TCGA-GD-A3OP | Bladder Urothelial Carcinoma | Bladder | Bladder | Yes |
| 98 | TCGA-S5-A6DX | Bladder Urothelial Carcinoma | Bladder | Bladder | Yes |
| 99 | TCGA-CV-A45V | Head and Neck Squamous Cell Carcinoma | Head and Neck | Head and Neck | Yes |
| 100 | TCGA-IQ-A61G | Head and Neck Squamous Cell Carcinoma | Head and Neck | Head and Neck | Yes |
| 101 | TCGA-B0-5092 | Kidney Renal Clear Cell Carcinoma | Kidney | Kidney | Yes |
| 102 | TCGA-HD-7229 | Head and Neck Squamous Cell Carcinoma | Head and Neck | Head and Neck | Yes |
| 103 | TCGA-HD-8224 | Head and Neck Squamous Cell Carcinoma | Head and Neck | Esophagus | No |
| 104 | TCGA-A6-6142 | Colon Adenocarcinoma | Colorectal | Colorectal | Yes |
| 105 | TCGA-55-6968 | Lung Adenocarcinoma | Lung | Lung | Yes |
| 106 | TCGA-CN-A6UY | Head and Neck Squamous Cell Carcinoma | Head and Neck | Head and Neck | Yes |
| 107 | TCGA-VQ-A91Q | Stomach Adenocarcinoma | Stomach | Stomach | Yes |
| 108 | TCGA-B0-4846 | Kidney Renal Clear Cell Carcinoma | Kidney | Kidney | Yes |
| 109 | TCGA-BT-A20V | Bladder Urothelial Carcinoma | Bladder | Bladder | Yes |
| 110 | TCGA-XF-AAMH | Bladder Urothelial Carcinoma | Bladder | Bladder | Yes |
| 111 | TCGA-B0-5115 | Kidney Renal Clear Cell Carcinoma | Kidney | Kidney | Yes |
| 112 | TCGA-5L-AAT1 | Breast Invasive Carcinoma | Breast | Breast | Yes |
| 113 | TCGA-CN-A641 | Head and Neck Squamous Cell Carcinoma | Head and Neck | Head and Neck | Yes |
| 114 | TCGA-CW-5591 | Kidney Renal Clear Cell Carcinoma | Kidney | Kidney | Yes |
| 115 | TCGA-2F-A9KO | Bladder Urothelial Carcinoma | Bladder | Bladder | Yes |
| 116 | TCGA-DK-A3IK | Bladder Urothelial Carcinoma | Bladder | Bladder | Yes |
| 117 | TCGA-CV-A45Y | Head and Neck Squamous Cell Carcinoma | Head and Neck | Head and Neck | Yes |
| 118 | TCGA-UY-A8OB | Bladder Urothelial Carcinoma | Bladder | Bladder | Yes |
| 119 | TCGA-UF-A7JD | Head and Neck Squamous Cell Carcinoma | Head and Neck | Head and Neck | Yes |
| 120 | TCGA-AZ-4614 | Colon Adenocarcinoma | Colorectal | Colorectal | Yes |
| 121 | TCGA-CV-6948 | Head and Neck Squamous Cell Carcinoma | Head and Neck | Esophagus | No |
| 122 | TCGA-BB-7864 | Head and Neck Squamous Cell Carcinoma | Head and Neck | Head and Neck | Yes |
| 123 | TCGA-XF-A9SY | Bladder Urothelial Carcinoma | Bladder | Bladder | Yes |
| 124 | TCGA-XF-A9SK | Bladder Urothelial Carcinoma | Bladder | Bladder | Yes |
| 125 | TCGA-A6-2671 | Colon Adenocarcinoma | Colorectal | Colorectal | Yes |
| 126 | TCGA-R5-A7ZI | Stomach Adenocarcinoma | Stomach | Stomach | Yes |
| 127 | TCGA-P3-A6T8 | Head and Neck Squamous Cell Carcinoma | Head and Neck | Head and Neck | Yes |
| 128 | TCGA-CZ-5461 | Kidney Renal Clear Cell Carcinoma | Kidney | Kidney | Yes |
| 129 | TCGA-FT-A61P | Bladder Urothelial Carcinoma | Bladder | Bladder | Yes |
| 130 | TCGA-E3-A3E5 | Thyroid Carcinoma | Thyroid | Thyroid | Yes |
| 131 | TCGA-CG-5716 | Stomach Adenocarcinoma | Stomach | Stomach | Yes |
| 132 | TCGA-UF-A71A | Head and Neck Squamous Cell Carcinoma | Head and Neck | Esophagus | No |
| 133 | TCGA-ZF-AA5H | Bladder Urothelial Carcinoma | Bladder | Bladder | Yes |
| 134 | TCGA-QK-A8Z9 | Head and Neck Squamous Cell Carcinoma | Head and Neck | Head and Neck | Yes |
| 135 | TCGA-CN-5369 | Head and Neck Squamous Cell Carcinoma | Head and Neck | Head and Neck | Yes |
| 136 | TCGA-E8-A3X7 | Thyroid Carcinoma | Thyroid | Thyroid | Yes |
| 137 | TCGA-FD-A6TA | Bladder Urothelial Carcinoma | Bladder | Bladder | Yes |
| 138 | TCGA-CW-5585 | Kidney Renal Clear Cell Carcinoma | Kidney | Kidney | Yes |
| 139 | TCGA-OR-A5J2 | Adrenocortical Carcinoma | Adrenal Gland | Adrenal Gland | Yes |
| 140 | TCGA-DK-A1A6 | Bladder Urothelial Carcinoma | Bladder | Bladder | Yes |
| 141 | TCGA-OR-A5KY | Adrenocortical Carcinoma | Adrenal Gland | Adrenal Gland | Yes |
| 142 | TCGA-BH-A1FH | Breast Invasive Carcinoma | Breast | Breast | Yes |
| 143 | TCGA-MT-A67G | Head and Neck Squamous Cell Carcinoma | Head and Neck | Head and Neck | Yes |
