# Supplementary material for: Predicting cancer origins with a DNA methylation-based deep neural network model
Source: PLoS One. 2020 May 8;15(5):e0226461. doi: 10.1371/journal.pone.0226461 (PMC7209244; doi:10.1371/journal.pone.0226461)
Supplement: S5 Table — (DOCX) [file pone.0226461.s005.docx]

**S5 Table. Cancer origin predictions for 581 samples from GEO datasets**

|  | **GEO_ID** | **Primary_site** | **Prediction** | **Correct** |
| --- | --- | --- | --- | --- |
| 1 | GSM2060832 | Adrenal Gland | Lung | No |
| 2 | GSM2060833 | Adrenal Gland | Adrenal Gland | Yes |
| 3 | GSM2060834 | Adrenal Gland | Lung | No |
| 4 | GSM2060835 | Adrenal Gland | Adrenal Gland | Yes |
| 5 | GSM2060836 | Adrenal Gland | Adrenal Gland | Yes |
| 6 | GSM2060837 | Adrenal Gland | Soft Tissue | No |
| 7 | GSM2060838 | Adrenal Gland | Adrenal Gland | Yes |
| 8 | GSM2060839 | Adrenal Gland | Adrenal Gland | Yes |
| 9 | GSM2060840 | Adrenal Gland | Adrenal Gland | Yes |
| 10 | GSM2060841 | Adrenal Gland | Adrenal Gland | Yes |
| 11 | GSM2060842 | Adrenal Gland | Adrenal Gland | Yes |
| 12 | GSM2060843 | Adrenal Gland | Adrenal Gland | Yes |
| 13 | GSM2060844 | Adrenal Gland | Adrenal Gland | Yes |
| 14 | GSM2060845 | Adrenal Gland | Adrenal Gland | Yes |
| 15 | GSM2060846 | Adrenal Gland | Soft Tissue | No |
| 16 | GSM2060847 | Adrenal Gland | Adrenal Gland | Yes |
| 17 | GSM2060848 | Adrenal Gland | Adrenal Gland | Yes |
| 18 | GSM2060849 | Adrenal Gland | Adrenal Gland | Yes |
| 19 | GSM2075589 | Breast | Breast | Yes |
| 20 | GSM2075590 | Breast | Breast | Yes |
| 21 | GSM2075591 | Breast | Breast | Yes |
| 22 | GSM2075592 | Breast | Breast | Yes |
| 23 | GSM2075593 | Breast | Breast | Yes |
| 24 | GSM2075594 | Breast | Breast | Yes |
| 25 | GSM2075595 | Breast | Breast | Yes |
| 26 | GSM2075596 | Breast | Breast | Yes |
| 27 | GSM2075597 | Breast | Breast | Yes |
| 28 | GSM2075598 | Breast | Breast | Yes |
| 29 | GSM2075599 | Breast | Breast | Yes |
| 30 | GSM2075600 | Breast | Breast | Yes |
| 31 | GSM2075601 | Breast | Breast | Yes |
| 32 | GSM2075602 | Breast | Breast | Yes |
| 33 | GSM2075603 | Breast | Breast | Yes |
| 34 | GSM2075604 | Breast | Breast | Yes |
| 35 | GSM2075605 | Breast | Breast | Yes |
| 36 | GSM2075606 | Breast | Breast | Yes |
| 37 | GSM2075607 | Breast | Breast | Yes |
| 38 | GSM2075608 | Breast | Breast | Yes |
| 39 | GSM2075609 | Breast | Breast | Yes |
| 40 | GSM2075610 | Breast | Breast | Yes |
| 41 | GSM2075611 | Breast | Breast | Yes |
| 42 | GSM2075612 | Breast | Breast | Yes |
| 43 | GSM2075613 | Breast | Lung | No |
| 44 | GSM2075614 | Breast | Breast | Yes |
| 45 | GSM2075615 | Breast | Breast | Yes |
| 46 | GSM2075616 | Breast | Breast | Yes |
| 47 | GSM2075617 | Breast | Breast | Yes |
| 48 | GSM2075618 | Breast | Breast | Yes |
| 49 | GSM2075619 | Breast | Breast | Yes |
| 50 | GSM2075620 | Breast | Breast | Yes |
| 51 | GSM2075621 | Breast | Breast | Yes |
| 52 | GSM2075622 | Breast | Breast | Yes |
| 53 | GSM2075623 | Breast | Breast | Yes |
| 54 | GSM2714569 | Colorectal | Colorectal | Yes |
| 55 | GSM2714570 | Colorectal | Colorectal | Yes |
| 56 | GSM2714573 | Colorectal | Colorectal | Yes |
| 57 | GSM2714574 | Colorectal | Colorectal | Yes |
| 58 | GSM2714577 | Colorectal | Colorectal | Yes |
| 59 | GSM2714579 | Colorectal | Colorectal | Yes |
| 60 | GSM2714580 | Colorectal | Colorectal | Yes |
| 61 | GSM2714583 | Colorectal | Colorectal | Yes |
| 62 | GSM2714584 | Colorectal | Colorectal | Yes |
| 63 | GSM2714586 | Colorectal | Colorectal | Yes |
| 64 | GSM2714590 | Colorectal | Colorectal | Yes |
| 65 | GSM2714591 | Colorectal | Colorectal | Yes |
| 66 | GSM2714594 | Colorectal | Colorectal | Yes |
| 67 | GSM2714595 | Colorectal | Colorectal | Yes |
| 68 | GSM2714596 | Colorectal | Colorectal | Yes |
| 69 | GSM2714598 | Colorectal | Colorectal | Yes |
| 70 | GSM2714599 | Colorectal | Colorectal | Yes |
| 71 | GSM2714602 | Colorectal | Colorectal | Yes |
| 72 | GSM2714603 | Colorectal | Colorectal | Yes |
| 73 | GSM2714606 | Colorectal | Colorectal | Yes |
| 74 | GSM2714607 | Colorectal | Colorectal | Yes |
| 75 | GSM2714610 | Colorectal | Colorectal | Yes |
| 76 | GSM2714611 | Colorectal | Stomach | No |
| 77 | GSM2714614 | Colorectal | Colorectal | Yes |
| 78 | GSM2714615 | Colorectal | Colorectal | Yes |
| 79 | GSM2714618 | Colorectal | Colorectal | Yes |
| 80 | GSM2714619 | Colorectal | Colorectal | Yes |
| 81 | GSM2714622 | Colorectal | Colorectal | Yes |
| 82 | GSM2714623 | Colorectal | Colorectal | Yes |
| 83 | GSM2714626 | Colorectal | Colorectal | Yes |
| 84 | GSM2714629 | Colorectal | Colorectal | Yes |
| 85 | GSM2714630 | Colorectal | Colorectal | Yes |
| 86 | GSM2714631 | Colorectal | Colorectal | Yes |
| 87 | GSM2714634 | Colorectal | Colorectal | Yes |
| 88 | GSM2714635 | Colorectal | Colorectal | Yes |
| 89 | GSM2714641 | Colorectal | Colorectal | Yes |
| 90 | GSM2714649 | Colorectal | Colorectal | Yes |
| 91 | GSM2714650 | Colorectal | Colorectal | Yes |
| 92 | GSM2714651 | Colorectal | Colorectal | Yes |
| 93 | GSM2714652 | Colorectal | Colorectal | Yes |
| 94 | GSM2714653 | Colorectal | Colorectal | Yes |
| 95 | GSM2714655 | Colorectal | Colorectal | Yes |
| 96 | GSM2714656 | Colorectal | Colorectal | Yes |
| 97 | GSM2714657 | Colorectal | Colorectal | Yes |
| 98 | GSM2714664 | Colorectal | Colorectal | Yes |
| 99 | GSM2714669 | Colorectal | Colorectal | Yes |
| 100 | GSM2714671 | Colorectal | Colorectal | Yes |
| 101 | GSM2714672 | Colorectal | Colorectal | Yes |
| 102 | GSM2714675 | Colorectal | Colorectal | Yes |
| 103 | GSM2714676 | Colorectal | Colorectal | Yes |
| 104 | GSM2714677 | Colorectal | Colorectal | Yes |
| 105 | GSM2714678 | Colorectal | Colorectal | Yes |
| 106 | GSM2714681 | Colorectal | Colorectal | Yes |
| 107 | GSM2714689 | Colorectal | Colorectal | Yes |
| 108 | GSM2714691 | Colorectal | Colorectal | Yes |
| 109 | GSM2714692 | Colorectal | Colorectal | Yes |
| 110 | GSM2714694 | Colorectal | Colorectal | Yes |
| 111 | GSM2714695 | Colorectal | Colorectal | Yes |
| 112 | GSM2714696 | Colorectal | Colorectal | Yes |
| 113 | GSM2714703 | Colorectal | Lung | No |
| 114 | GSM2714704 | Colorectal | Colorectal | Yes |
| 115 | GSM2714705 | Colorectal | Colorectal | Yes |
| 116 | GSM2714706 | Colorectal | Colorectal | Yes |
| 117 | GSM2714707 | Colorectal | Colorectal | Yes |
| 118 | GSM2714712 | Colorectal | Colorectal | Yes |
| 119 | GSM2714713 | Colorectal | Colorectal | Yes |
| 120 | GSM2714715 | Colorectal | Colorectal | Yes |
| 121 | GSM2714717 | Colorectal | Colorectal | Yes |
| 122 | GSM2714719 | Colorectal | Colorectal | Yes |
| 123 | GSM2714754 | Colorectal | Colorectal | Yes |
| 124 | GSM2714756 | Colorectal | Breast | No |
| 125 | GSM2714758 | Colorectal | Colorectal | Yes |
| 126 | GSM2714760 | Colorectal | Colorectal | Yes |
| 127 | GSM2714762 | Colorectal | Stomach | No |
| 128 | GSM2714764 | Colorectal | Colorectal | Yes |
| 129 | GSM2714787 | Colorectal | Colorectal | Yes |
| 130 | GSM2714789 | Colorectal | Colorectal | Yes |
| 131 | GSM2714793 | Colorectal | Colorectal | Yes |
| 132 | GSM2714795 | Colorectal | Colorectal | Yes |
| 133 | GSM2714797 | Colorectal | Colorectal | Yes |
| 134 | GSM2714799 | Colorectal | Colorectal | Yes |
| 135 | GSM2714801 | Colorectal | Colorectal | Yes |
| 136 | GSM2714803 | Colorectal | Colorectal | Yes |
| 137 | GSM2714804 | Colorectal | Colorectal | Yes |
| 138 | GSM2714806 | Colorectal | Colorectal | Yes |
| 139 | GSM2714807 | Colorectal | Colorectal | Yes |
| 140 | GSM2714810 | Colorectal | Colorectal | Yes |
| 141 | GSM2714811 | Colorectal | Colorectal | Yes |
| 142 | GSM2714815 | Colorectal | Colorectal | Yes |
| 143 | GSM2714816 | Colorectal | Colorectal | Yes |
| 144 | GSM2714819 | Colorectal | Colorectal | Yes |
| 145 | GSM2714821 | Colorectal | Colorectal | Yes |
| 146 | GSM2714822 | Colorectal | Colorectal | Yes |
| 147 | GSM2714825 | Colorectal | Colorectal | Yes |
| 148 | GSM2714826 | Colorectal | Colorectal | Yes |
| 149 | GSM2714829 | Colorectal | Colorectal | Yes |
| 150 | GSM2714830 | Colorectal | Colorectal | Yes |
| 151 | GSM2714833 | Colorectal | Colorectal | Yes |
| 152 | GSM2714834 | Colorectal | Colorectal | Yes |
| 153 | GSM2714837 | Colorectal | Colorectal | Yes |
| 154 | GSM2714838 | Colorectal | Colorectal | Yes |
| 155 | GSM2714840 | Colorectal | Colorectal | Yes |
| 156 | GSM2714841 | Colorectal | Colorectal | Yes |
| 157 | GSM2714844 | Colorectal | Colorectal | Yes |
| 158 | GSM2714845 | Colorectal | Colorectal | Yes |
| 159 | GSM2714848 | Colorectal | Colorectal | Yes |
| 160 | GSM2714849 | Colorectal | Colorectal | Yes |
| 161 | GSM2714850 | Colorectal | Colorectal | Yes |
| 162 | GSM2714853 | Colorectal | Colorectal | Yes |
| 163 | GSM2714854 | Colorectal | Colorectal | Yes |
| 164 | GSM2714857 | Colorectal | Colorectal | Yes |
| 165 | GSM2714858 | Colorectal | Colorectal | Yes |
| 166 | GSM937820 | Head and Neck | Head and Neck | Yes |
| 167 | GSM937821 | Head and Neck | Lung | No |
| 168 | GSM937822 | Head and Neck | Head and Neck | Yes |
| 169 | GSM937823 | Head and Neck | Head and Neck | Yes |
| 170 | GSM937824 | Head and Neck | Head and Neck | Yes |
| 171 | GSM937825 | Head and Neck | Head and Neck | Yes |
| 172 | GSM2391565 | Liver | Liver | Yes |
| 173 | GSM2391566 | Liver | Liver | Yes |
| 174 | GSM2391567 | Liver | Liver | Yes |
| 175 | GSM2391568 | Liver | Liver | Yes |
| 176 | GSM2391569 | Liver | Liver | Yes |
| 177 | GSM2391570 | Liver | Liver | Yes |
| 178 | GSM2391571 | Liver | Liver | Yes |
| 179 | GSM2391572 | Liver | Liver | Yes |
| 180 | GSM2391573 | Liver | Liver | Yes |
| 181 | GSM2391574 | Liver | Liver | Yes |
| 182 | GSM2391575 | Liver | Liver | Yes |
| 183 | GSM2391576 | Liver | Liver | Yes |
| 184 | GSM2391577 | Liver | Liver | Yes |
| 185 | GSM2391578 | Liver | Liver | Yes |
| 186 | GSM2391579 | Liver | Liver | Yes |
| 187 | GSM2391580 | Liver | Liver | Yes |
| 188 | GSM2391581 | Liver | Liver | Yes |
| 189 | GSM2391582 | Liver | Liver | Yes |
| 190 | GSM2391583 | Liver | Liver | Yes |
| 191 | GSM2391584 | Liver | Liver | Yes |
| 192 | GSM2391585 | Liver | Liver | Yes |
| 193 | GSM2391586 | Liver | Liver | Yes |
| 194 | GSM2391587 | Liver | Liver | Yes |
| 195 | GSM2391588 | Liver | Liver | Yes |
| 196 | GSM2391589 | Liver | Liver | Yes |
| 197 | GSM2391590 | Liver | Liver | Yes |
| 198 | GSM2391591 | Liver | Liver | Yes |
| 199 | GSM2391592 | Liver | Liver | Yes |
| 200 | GSM2391593 | Liver | Liver | Yes |
| 201 | GSM2391594 | Liver | Liver | Yes |
| 202 | GSM2391595 | Liver | Liver | Yes |
| 203 | GSM2391596 | Liver | Liver | Yes |
| 204 | GSM2391597 | Liver | Liver | Yes |
| 205 | GSM2391598 | Liver | Liver | Yes |
| 206 | GSM2391599 | Liver | Liver | Yes |
| 207 | GSM2391600 | Liver | Liver | Yes |
| 208 | GSM2391601 | Liver | Liver | Yes |
| 209 | GSM1194354 | Pancreas | Lung | No |
| 210 | GSM1194355 | Pancreas | Pancreas | Yes |
| 211 | GSM1194357 | Pancreas | Pancreas | Yes |
| 212 | GSM1194358 | Pancreas | Pancreas | Yes |
| 213 | GSM1194359 | Pancreas | Pancreas | Yes |
| 214 | GSM1194360 | Pancreas | Pancreas | Yes |
| 215 | GSM1194361 | Pancreas | Pancreas | Yes |
| 216 | GSM1194363 | Pancreas | Lung | No |
| 217 | GSM1194364 | Pancreas | Pancreas | Yes |
| 218 | GSM1194366 | Pancreas | Pancreas | Yes |
| 219 | GSM1194367 | Pancreas | Pancreas | Yes |
| 220 | GSM1194368 | Pancreas | Pancreas | Yes |
| 221 | GSM1194369 | Pancreas | Pancreas | Yes |
| 222 | GSM1194370 | Pancreas | Pancreas | Yes |
| 223 | GSM1194371 | Pancreas | Pancreas | Yes |
| 224 | GSM1194372 | Pancreas | Pancreas | Yes |
| 225 | GSM1194373 | Pancreas | Stomach | No |
| 226 | GSM1194374 | Pancreas | Stomach | No |
| 227 | GSM1194375 | Pancreas | Pancreas | Yes |
| 228 | GSM1194376 | Pancreas | Pancreas | Yes |
| 229 | GSM1194377 | Pancreas | Stomach | No |
| 230 | GSM1194378 | Pancreas | Pancreas | Yes |
| 231 | GSM1194379 | Pancreas | Stomach | No |
| 232 | GSM1194380 | Pancreas | Pancreas | Yes |
| 233 | GSM1194381 | Pancreas | Pancreas | Yes |
| 234 | GSM1194382 | Pancreas | Stomach | No |
| 235 | GSM1194383 | Pancreas | Pancreas | Yes |
| 236 | GSM1194384 | Pancreas | Pancreas | Yes |
| 237 | GSM1194385 | Pancreas | Liver | No |
| 238 | GSM1194386 | Pancreas | Stomach | No |
| 239 | GSM1194387 | Pancreas | Pancreas | Yes |
| 240 | GSM1194388 | Pancreas | Stomach | No |
| 241 | GSM1194389 | Pancreas | Pancreas | Yes |
| 242 | GSM1194390 | Pancreas | Stomach | No |
| 243 | GSM1194391 | Pancreas | Pancreas | Yes |
| 244 | GSM1194392 | Pancreas | Pancreas | Yes |
| 245 | GSM1194393 | Pancreas | Pancreas | Yes |
| 246 | GSM1194394 | Pancreas | Pancreas | Yes |
| 247 | GSM1194395 | Pancreas | Pancreas | Yes |
| 248 | GSM1194396 | Pancreas | Pancreas | Yes |
| 249 | GSM1194397 | Pancreas | Pancreas | Yes |
| 250 | GSM1194398 | Pancreas | Pancreas | Yes |
| 251 | GSM1194399 | Pancreas | Pancreas | Yes |
| 252 | GSM1194400 | Pancreas | Pancreas | Yes |
| 253 | GSM1194401 | Pancreas | Stomach | No |
| 254 | GSM1194402 | Pancreas | Pancreas | Yes |
| 255 | GSM1194403 | Pancreas | Stomach | No |
| 256 | GSM1194404 | Pancreas | Pancreas | Yes |
| 257 | GSM1194405 | Pancreas | Pancreas | Yes |
| 258 | GSM1194406 | Pancreas | Pancreas | Yes |
| 259 | GSM1194407 | Pancreas | Pancreas | Yes |
| 260 | GSM1194408 | Pancreas | Stomach | No |
| 261 | GSM1194410 | Pancreas | Pancreas | Yes |
| 262 | GSM1194411 | Pancreas | Pancreas | Yes |
| 263 | GSM1194412 | Pancreas | Pancreas | Yes |
| 264 | GSM1194413 | Pancreas | Pancreas | Yes |
| 265 | GSM1194414 | Pancreas | Pancreas | Yes |
| 266 | GSM1194415 | Pancreas | Pancreas | Yes |
| 267 | GSM1194416 | Pancreas | Pancreas | Yes |
| 268 | GSM1194417 | Pancreas | Pancreas | Yes |
| 269 | GSM1194418 | Pancreas | Pancreas | Yes |
| 270 | GSM1194419 | Pancreas | Pancreas | Yes |
| 271 | GSM1194420 | Pancreas | Liver | No |
| 272 | GSM1194421 | Pancreas | Stomach | No |
| 273 | GSM1194423 | Pancreas | Pancreas | Yes |
| 274 | GSM1194424 | Pancreas | Pancreas | Yes |
| 275 | GSM1194425 | Pancreas | Lung | No |
| 276 | GSM1194426 | Pancreas | Pancreas | Yes |
| 277 | GSM1194427 | Pancreas | Pancreas | Yes |
| 278 | GSM1194428 | Pancreas | Pancreas | Yes |
| 279 | GSM1194429 | Pancreas | Pancreas | Yes |
| 280 | GSM1194430 | Pancreas | Pancreas | Yes |
| 281 | GSM1194433 | Pancreas | Pancreas | Yes |
| 282 | GSM1194434 | Pancreas | Lung | No |
| 283 | GSM1194435 | Pancreas | Pancreas | Yes |
| 284 | GSM1194436 | Pancreas | Pancreas | Yes |
| 285 | GSM1194437 | Pancreas | Pancreas | Yes |
| 286 | GSM1194438 | Pancreas | Pancreas | Yes |
| 287 | GSM1194439 | Pancreas | Pancreas | Yes |
| 288 | GSM1194441 | Pancreas | Pancreas | Yes |
| 289 | GSM1194442 | Pancreas | Pancreas | Yes |
| 290 | GSM1194443 | Pancreas | Pancreas | Yes |
| 291 | GSM1194444 | Pancreas | Pancreas | Yes |
| 292 | GSM1194445 | Pancreas | Pancreas | Yes |
| 293 | GSM1194446 | Pancreas | Lung | No |
| 294 | GSM1194448 | Pancreas | Pancreas | Yes |
| 295 | GSM1194449 | Pancreas | Pancreas | Yes |
| 296 | GSM1194450 | Pancreas | Pancreas | Yes |
| 297 | GSM1194451 | Pancreas | Lung | No |
| 298 | GSM1194452 | Pancreas | Pancreas | Yes |
| 299 | GSM1194454 | Pancreas | Pancreas | Yes |
| 300 | GSM1194455 | Pancreas | Pancreas | Yes |
| 301 | GSM1194456 | Pancreas | Pancreas | Yes |
| 302 | GSM1194457 | Pancreas | Pancreas | Yes |
| 303 | GSM1194458 | Pancreas | Pancreas | Yes |
| 304 | GSM1194459 | Pancreas | Stomach | No |
| 305 | GSM1194461 | Pancreas | Pancreas | Yes |
| 306 | GSM1194463 | Pancreas | Pancreas | Yes |
| 307 | GSM1194464 | Pancreas | Stomach | No |
| 308 | GSM1194465 | Pancreas | Pancreas | Yes |
| 309 | GSM1194466 | Pancreas | Pancreas | Yes |
| 310 | GSM1194467 | Pancreas | Pancreas | Yes |
| 311 | GSM1194469 | Pancreas | Liver | No |
| 312 | GSM1194470 | Pancreas | Pancreas | Yes |
| 313 | GSM1194471 | Pancreas | Pancreas | Yes |
| 314 | GSM1194474 | Pancreas | Pancreas | Yes |
| 315 | GSM1194475 | Pancreas | Pancreas | Yes |
| 316 | GSM1194476 | Pancreas | Pancreas | Yes |
| 317 | GSM1194479 | Pancreas | Pancreas | Yes |
| 318 | GSM1194480 | Pancreas | Pancreas | Yes |
| 319 | GSM1194481 | Pancreas | Pancreas | Yes |
| 320 | GSM1194482 | Pancreas | Pancreas | Yes |
| 321 | GSM1194483 | Pancreas | Pancreas | Yes |
| 322 | GSM1194484 | Pancreas | Pancreas | Yes |
| 323 | GSM1194485 | Pancreas | Pancreas | Yes |
| 324 | GSM1194486 | Pancreas | Pancreas | Yes |
| 325 | GSM1194487 | Pancreas | Stomach | No |
| 326 | GSM1194489 | Pancreas | Pancreas | Yes |
| 327 | GSM1194490 | Pancreas | Pancreas | Yes |
| 328 | GSM1194491 | Pancreas | Pancreas | Yes |
| 329 | GSM1194492 | Pancreas | Pancreas | Yes |
| 330 | GSM1194494 | Pancreas | Pancreas | Yes |
| 331 | GSM1194495 | Pancreas | Stomach | No |
| 332 | GSM1194496 | Pancreas | Stomach | No |
| 333 | GSM1194497 | Pancreas | Pancreas | Yes |
| 334 | GSM1194498 | Pancreas | Stomach | No |
| 335 | GSM1194501 | Pancreas | Pancreas | Yes |
| 336 | GSM1194502 | Pancreas | Pancreas | Yes |
| 337 | GSM1194503 | Pancreas | Pancreas | Yes |
| 338 | GSM1194504 | Pancreas | Pancreas | Yes |
| 339 | GSM1194505 | Pancreas | Pancreas | Yes |
| 340 | GSM1194506 | Pancreas | Pancreas | Yes |
| 341 | GSM1194507 | Pancreas | Pancreas | Yes |
| 342 | GSM1194508 | Pancreas | Pancreas | Yes |
| 343 | GSM1194509 | Pancreas | Pancreas | Yes |
| 344 | GSM1194510 | Pancreas | Pancreas | Yes |
| 345 | GSM1194511 | Pancreas | Pancreas | Yes |
| 346 | GSM1194512 | Pancreas | Pancreas | Yes |
| 347 | GSM1194513 | Pancreas | Stomach | No |
| 348 | GSM1194514 | Pancreas | Pancreas | Yes |
| 349 | GSM1194515 | Pancreas | Pancreas | Yes |
| 350 | GSM1194516 | Pancreas | Stomach | No |
| 351 | GSM1194517 | Pancreas | Stomach | No |
| 352 | GSM1194518 | Pancreas | Pancreas | Yes |
| 353 | GSM1194519 | Pancreas | Pancreas | Yes |
| 354 | GSM1194520 | Pancreas | Pancreas | Yes |
| 355 | GSM1194521 | Pancreas | Pancreas | Yes |
| 356 | GSM1194522 | Pancreas | Pancreas | Yes |
| 357 | GSM1194524 | Pancreas | Pancreas | Yes |
| 358 | GSM1194526 | Pancreas | Pancreas | Yes |
| 359 | GSM1194527 | Pancreas | Pancreas | Yes |
| 360 | GSM1194528 | Pancreas | Pancreas | Yes |
| 361 | GSM1194530 | Pancreas | Pancreas | Yes |
| 362 | GSM1194531 | Pancreas | Pancreas | Yes |
| 363 | GSM1194532 | Pancreas | Stomach | No |
| 364 | GSM1435224 | Pancreas | Pancreas | Yes |
| 365 | GSM1435226 | Pancreas | Pancreas | Yes |
| 366 | GSM1435228 | Pancreas | Pancreas | Yes |
| 367 | GSM1435230 | Pancreas | Stomach | No |
| 368 | GSM1435232 | Pancreas | Pancreas | Yes |
| 369 | GSM1435235 | Pancreas | Pancreas | Yes |
| 370 | GSM1435237 | Pancreas | Pancreas | Yes |
| 371 | GSM1435239 | Pancreas | Pancreas | Yes |
| 372 | GSM1435241 | Pancreas | Pancreas | Yes |
| 373 | GSM1435243 | Pancreas | Pancreas | Yes |
| 374 | GSM1435244 | Pancreas | Pancreas | Yes |
| 375 | GSM1435245 | Pancreas | Pancreas | Yes |
| 376 | GSM3053770 | Prostate | Prostate | Yes |
| 377 | GSM3053771 | Prostate | Prostate | Yes |
| 378 | GSM3053772 | Prostate | Prostate | Yes |
| 379 | GSM3053775 | Prostate | Prostate | Yes |
| 380 | GSM3053776 | Prostate | Prostate | Yes |
| 381 | GSM3053778 | Prostate | Prostate | Yes |
| 382 | GSM3053779 | Prostate | Prostate | Yes |
| 383 | GSM3053780 | Prostate | Prostate | Yes |
| 384 | GSM3053783 | Prostate | Prostate | Yes |
| 385 | GSM3053784 | Prostate | Prostate | Yes |
| 386 | GSM3053786 | Prostate | Prostate | Yes |
| 387 | GSM3053787 | Prostate | Prostate | Yes |
| 388 | GSM3053788 | Prostate | Prostate | Yes |
| 389 | GSM3053790 | Prostate | Prostate | Yes |
| 390 | GSM3053791 | Prostate | Prostate | Yes |
| 391 | GSM3053792 | Prostate | Prostate | Yes |
| 392 | GSM3053795 | Prostate | Prostate | Yes |
| 393 | GSM3053796 | Prostate | Prostate | Yes |
| 394 | GSM3053798 | Prostate | Prostate | Yes |
| 395 | GSM3053799 | Prostate | Prostate | Yes |
| 396 | GSM3053802 | Prostate | Prostate | Yes |
| 397 | GSM3053803 | Prostate | Prostate | Yes |
| 398 | GSM3053804 | Prostate | Prostate | Yes |
| 399 | GSM3053806 | Prostate | Prostate | Yes |
| 400 | GSM3053807 | Prostate | Prostate | Yes |
| 401 | GSM3053808 | Prostate | Prostate | Yes |
| 402 | GSM3053810 | Prostate | Prostate | Yes |
| 403 | GSM3053811 | Prostate | Prostate | Yes |
| 404 | GSM3053813 | Prostate | Prostate | Yes |
| 405 | GSM3053814 | Prostate | Prostate | Yes |
| 406 | GSM3053815 | Prostate | Prostate | Yes |
| 407 | GSM937258 | Prostate | Prostate | Yes |
| 408 | GSM937259 | Prostate | Prostate | Yes |
| 409 | GSM937260 | Prostate | Prostate | Yes |
| 410 | GSM937261 | Prostate | Prostate | Yes |
| 411 | GSM937262 | Prostate | Prostate | Yes |
| 412 | GSM937264 | Prostate | Prostate | Yes |
| 413 | GSM937266 | Prostate | Prostate | Yes |
| 414 | GSM937268 | Prostate | Prostate | Yes |
| 415 | GSM1897625 | Prostate | Prostate | Yes |
| 416 | GSM1897629 | Prostate | Prostate | Yes |
| 417 | GSM1897634 | Prostate | Prostate | Yes |
| 418 | GSM1897639 | Prostate | Prostate | Yes |
| 419 | GSM1897640 | Prostate | Prostate | Yes |
| 420 | GSM1897645 | Prostate | Prostate | Yes |
| 421 | GSM1897653 | Prostate | Prostate | Yes |
| 422 | GSM1897660 | Prostate | Prostate | Yes |
| 423 | GSM1897667 | Prostate | Prostate | Yes |
| 424 | GSM1897674 | Prostate | Prostate | Yes |
| 425 | GSM1897678 | Prostate | Prostate | Yes |
| 426 | GSM1897683 | Prostate | Prostate | Yes |
| 427 | GSM1897686 | Prostate | Prostate | Yes |
| 428 | GSM1897687 | Prostate | Prostate | Yes |
| 429 | GSM1897694 | Prostate | Prostate | Yes |
| 430 | GSM1897701 | Prostate | Prostate | Yes |
| 431 | GSM1897706 | Prostate | Prostate | Yes |
| 432 | GSM1897711 | Prostate | Prostate | Yes |
| 433 | GSM2311032 | Thyroid | Breast | No |
| 434 | GSM2311033 | Thyroid | Thyroid | Yes |
| 435 | GSM2311034 | Thyroid | Thyroid | Yes |
| 436 | GSM2311035 | Thyroid | Thyroid | Yes |
| 437 | GSM2311036 | Thyroid | Thyroid | Yes |
| 438 | GSM2311037 | Thyroid | Thyroid | Yes |
| 439 | GSM2311038 | Thyroid | Thyroid | Yes |
| 440 | GSM2311039 | Thyroid | Thyroid | Yes |
| 441 | GSM2311040 | Thyroid | Thyroid | Yes |
| 442 | GSM2311041 | Thyroid | Thyroid | Yes |
| 443 | GSM2311042 | Thyroid | Thyroid | Yes |
| 444 | GSM2311043 | Thyroid | Thyroid | Yes |
| 445 | GSM2311044 | Thyroid | Thyroid | Yes |
| 446 | GSM2311045 | Thyroid | Thyroid | Yes |
| 447 | GSM2311046 | Thyroid | Thyroid | Yes |
| 448 | GSM2311047 | Thyroid | Thyroid | Yes |
| 449 | GSM2311048 | Thyroid | Thyroid | Yes |
| 450 | GSM2311049 | Thyroid | Thyroid | Yes |
| 451 | GSM2311050 | Thyroid | Thyroid | Yes |
| 452 | GSM2311051 | Thyroid | Thyroid | Yes |
| 453 | GSM2311052 | Thyroid | Thyroid | Yes |
| 454 | GSM2311053 | Thyroid | Thyroid | Yes |
| 455 | GSM2311054 | Thyroid | Thyroid | Yes |
| 456 | GSM2311055 | Thyroid | Thyroid | Yes |
| 457 | GSM2311056 | Thyroid | Thyroid | Yes |
| 458 | GSM2311057 | Thyroid | Thyroid | Yes |
| 459 | GSM2311058 | Thyroid | Thyroid | Yes |
| 460 | GSM2311059 | Thyroid | Thyroid | Yes |
| 461 | GSM2311060 | Thyroid | Thyroid | Yes |
| 462 | GSM2311061 | Thyroid | Thyroid | Yes |
| 463 | GSM2311062 | Thyroid | Thyroid | Yes |
| 464 | GSM2311063 | Thyroid | Thyroid | Yes |
| 465 | GSM2311064 | Thyroid | Thyroid | Yes |
| 466 | GSM2311065 | Thyroid | Thyroid | Yes |
| 467 | GSM2311066 | Thyroid | Thyroid | Yes |
| 468 | GSM2311067 | Thyroid | Thyroid | Yes |
| 469 | GSM2311068 | Thyroid | Thyroid | Yes |
| 470 | GSM2311069 | Thyroid | Thyroid | Yes |
| 471 | GSM2311070 | Thyroid | Thyroid | Yes |
| 472 | GSM2311071 | Thyroid | Thyroid | Yes |
| 473 | GSM2311072 | Thyroid | Thyroid | Yes |
| 474 | GSM2311073 | Thyroid | Thyroid | Yes |
| 475 | GSM2311074 | Thyroid | Thyroid | Yes |
| 476 | GSM2311075 | Thyroid | Thyroid | Yes |
| 477 | GSM2311076 | Thyroid | Thyroid | Yes |
| 478 | GSM2311077 | Thyroid | Thyroid | Yes |
| 479 | GSM2311078 | Thyroid | Thyroid | Yes |
| 480 | GSM2311079 | Thyroid | Thyroid | Yes |
| 481 | GSM2311080 | Thyroid | Thyroid | Yes |
| 482 | GSM2311081 | Thyroid | Thyroid | Yes |
| 483 | GSM2311082 | Thyroid | Thyroid | Yes |
| 484 | GSM2311083 | Thyroid | Thyroid | Yes |
| 485 | GSM2311084 | Thyroid | Thyroid | Yes |
| 486 | GSM2311085 | Thyroid | Thyroid | Yes |
| 487 | GSM2311086 | Thyroid | Thyroid | Yes |
| 488 | GSM2311087 | Thyroid | Thyroid | Yes |
| 489 | GSM2311088 | Thyroid | Thyroid | Yes |
| 490 | GSM2311089 | Thyroid | Thyroid | Yes |
| 491 | GSM2311090 | Thyroid | Thyroid | Yes |
| 492 | GSM2311091 | Thyroid | Thyroid | Yes |
| 493 | GSM2311092 | Thyroid | Thyroid | Yes |
| 494 | GSM2311093 | Thyroid | Thyroid | Yes |
| 495 | GSM2311094 | Thyroid | Thyroid | Yes |
| 496 | GSM2311095 | Thyroid | Thyroid | Yes |
| 497 | GSM2311096 | Thyroid | Thyroid | Yes |
| 498 | GSM2311097 | Thyroid | Thyroid | Yes |
| 499 | GSM2311098 | Thyroid | Thyroid | Yes |
| 500 | GSM2311099 | Thyroid | Thyroid | Yes |
| 501 | GSM2311100 | Thyroid | Thyroid | Yes |
| 502 | GSM2311101 | Thyroid | Thyroid | Yes |
| 503 | GSM2311102 | Thyroid | Thyroid | Yes |
| 504 | GSM2311103 | Thyroid | Thyroid | Yes |
| 505 | GSM2311104 | Thyroid | Thyroid | Yes |
| 506 | GSM2311105 | Thyroid | Thyroid | Yes |
| 507 | GSM2311106 | Thyroid | Thyroid | Yes |
| 508 | GSM2311107 | Thyroid | Thyroid | Yes |
| 509 | GSM2311108 | Thyroid | Thyroid | Yes |
| 510 | GSM2311109 | Thyroid | Thyroid | Yes |
| 511 | GSM2311110 | Thyroid | Thyroid | Yes |
| 512 | GSM2311111 | Thyroid | Thyroid | Yes |
| 513 | GSM2311112 | Thyroid | Thyroid | Yes |
| 514 | GSM2311113 | Thyroid | Thyroid | Yes |
| 515 | GSM1278514 | Kidney | Kidney | Yes |
| 516 | GSM1278515 | Kidney | Kidney | Yes |
| 517 | GSM1278516 | Kidney | Kidney | Yes |
| 518 | GSM1278517 | Kidney | Kidney | Yes |
| 519 | GSM1278518 | Kidney | Kidney | Yes |
| 520 | GSM1278519 | Kidney | Kidney | Yes |
| 521 | GSM1278520 | Kidney | Kidney | Yes |
| 522 | GSM1278521 | Kidney | Kidney | Yes |
| 523 | GSM1278522 | Kidney | Kidney | Yes |
| 524 | GSM1278523 | Kidney | Kidney | Yes |
| 525 | GSM1278524 | Kidney | Kidney | Yes |
| 526 | GSM1278525 | Kidney | Kidney | Yes |
| 527 | GSM1278526 | Kidney | Kidney | Yes |
| 528 | GSM1278527 | Kidney | Kidney | Yes |
| 529 | GSM1278528 | Kidney | Kidney | Yes |
| 530 | GSM1278529 | Kidney | Kidney | Yes |
| 531 | GSM1278530 | Kidney | Kidney | Yes |
| 532 | GSM1278536 | Bladder | Bladder | Yes |
| 533 | GSM1278537 | Bladder | Bladder | Yes |
| 534 | GSM1278538 | Bladder | Bladder | Yes |
| 535 | GSM1278539 | Bladder | Bladder | Yes |
| 536 | GSM1278540 | Bladder | Bladder | Yes |
| 537 | GSM1278541 | Bladder | Bladder | Yes |
| 538 | GSM1278542 | Bladder | Bladder | Yes |
| 539 | GSM1278543 | Bladder | Bladder | Yes |
| 540 | GSM1278544 | Bladder | Bladder | Yes |
| 541 | GSM1278545 | Bladder | Bladder | Yes |
| 542 | GSM1278546 | Bladder | Bladder | Yes |
| 543 | GSM1278547 | Bladder | Bladder | Yes |
| 544 | GSM1278548 | Bladder | Bladder | Yes |
| 545 | GSM1278549 | Bladder | Bladder | Yes |
| 546 | GSM1278550 | Bladder | Bladder | Yes |
| 547 | GSM1278551 | Bladder | Bladder | Yes |
| 548 | GSM1278552 | Bladder | Bladder | Yes |
| 549 | GSM1278553 | Bladder | Bladder | Yes |
| 550 | GSM1278554 | Bladder | Bladder | Yes |
| 551 | GSM1278555 | Bladder | Bladder | Yes |
| 552 | GSM1278556 | Bladder | Bladder | Yes |
| 553 | GSM1278557 | Bladder | Bladder | Yes |
| 554 | GSM1278558 | Bladder | Bladder | Yes |
| 555 | GSM1278559 | Bladder | Bladder | Yes |
| 556 | GSM1278560 | Bladder | Bladder | Yes |
| 557 | GSM1278566 | Prostate | Prostate | Yes |
| 558 | GSM1278567 | Prostate | Prostate | Yes |
| 559 | GSM1278568 | Prostate | Prostate | Yes |
| 560 | GSM1278569 | Prostate | Prostate | Yes |
| 561 | GSM1278570 | Prostate | Prostate | Yes |
| 562 | GSM1278571 | Prostate | Prostate | Yes |
| 563 | GSM1278572 | Prostate | Prostate | Yes |
| 564 | GSM1278573 | Prostate | Prostate | Yes |
| 565 | GSM1278574 | Prostate | Prostate | Yes |
| 566 | GSM1278575 | Prostate | Prostate | Yes |
| 567 | GSM1278576 | Prostate | Prostate | Yes |
| 568 | GSM1278577 | Prostate | Prostate | Yes |
| 569 | GSM1278578 | Prostate | Prostate | Yes |
| 570 | GSM1278579 | Prostate | Prostate | Yes |
| 571 | GSM1278580 | Prostate | Prostate | Yes |
| 572 | GSM1278581 | Prostate | Prostate | Yes |
| 573 | GSM1278582 | Prostate | Prostate | Yes |
| 574 | GSM1278583 | Prostate | Prostate | Yes |
| 575 | GSM1278584 | Prostate | Prostate | Yes |
| 576 | GSM1278585 | Prostate | Prostate | Yes |
| 577 | GSM1278586 | Prostate | Prostate | Yes |
| 578 | GSM1278587 | Prostate | Prostate | Yes |
| 579 | GSM1278588 | Prostate | Prostate | Yes |
| 580 | GSM1278589 | Prostate | Prostate | Yes |
| 581 | GSM1278590 | Prostate | Prostate | Yes |
