# Supplementary material for: Predicting cancer origins with a DNA methylation-based deep neural network model
Source: PLoS One. 2020 May 8;15(5):e0226461. doi: 10.1371/journal.pone.0226461 (PMC7209244; doi:10.1371/journal.pone.0226461)
Supplement: S7 Table — (DOCX) [file pone.0226461.s007.docx]

**S7 Table Cancer cell type predictions for 391 cancer cell lines**

|  | Cell_line | COSMIC identifier | Tissue | Prediction | Correct |
| --- | --- | --- | --- | --- | --- |
| 0 | A253 | 906794 | Head and Neck | Head and Neck | Yes |
| 1 | BB30-HNC | 753531 | Head and Neck | Head and Neck | Yes |
| 2 | BB49-HNC | 753532 | Head and Neck | Head and Neck | Yes |
| 3 | BHY | 753535 | Head and Neck | Head and Neck | Yes |
| 4 | BICR10 | 1290724 | Head and Neck | Head and Neck | Yes |
| 5 | BICR22 | 1240121 | Head and Neck | Head and Neck | Yes |
| 6 | BICR31 | 1290725 | Head and Neck | Head and Neck | Yes |
| 7 | BICR78 | 1240122 | Head and Neck | Head and Neck | Yes |
| 8 | CAL-27 | 910916 | Head and Neck | Head and Neck | Yes |
| 9 | CAL-33 | 753541 | Head and Neck | Head and Neck | Yes |
| 10 | DOK | 910936 | Head and Neck | Head and Neck | Yes |
| 11 | FADU | 906863 | Head and Neck | Esophagus | No |
| 12 | H3118 | 1240140 | Head and Neck | Lung | No |
| 13 | HN | 907059 | Head and Neck | Head and Neck | Yes |
| 14 | HO-1-N-1 | 924111 | Head and Neck | Head and Neck | Yes |
| 15 | HSC-2 | 753562 | Head and Neck | Head and Neck | Yes |
| 16 | HSC-3 | 907061 | Head and Neck | Head and Neck | Yes |
| 17 | HSC-4 | 907062 | Head and Neck | Head and Neck | Yes |
| 18 | JHU-011 | 1240161 | Head and Neck | Head and Neck | Yes |
| 19 | JHU-022 | 1240162 | Head and Neck | Lung | No |
| 20 | KON | 1298215 | Head and Neck | Head and Neck | Yes |
| 21 | KOSC-2 | 753570 | Head and Neck | Head and Neck | Yes |
| 22 | LB771-HNC | 753583 | Head and Neck | Lung | No |
| 23 | OSC-19 | 1298362 | Head and Neck | Head and Neck | Yes |
| 24 | OSC-20 | 1240196 | Head and Neck | Head and Neck | Yes |
| 25 | PCI-15A | 1240204 | Head and Neck | Esophagus | No |
| 26 | PCI-30 | 1298529 | Head and Neck | Head and Neck | Yes |
| 27 | PCI-38 | 1240205 | Head and Neck | Head and Neck | Yes |
| 28 | PCI-4B | 1298531 | Head and Neck | Head and Neck | Yes |
| 29 | PCI-6A | 1240206 | Head and Neck | Head and Neck | Yes |
| 30 | RPMI-2650 | 909700 | Head and Neck | Adrenal Gland | No |
| 31 | SAS | 909708 | Head and Neck | Head and Neck | Yes |
| 32 | SAT | 1299050 | Head and Neck | Head and Neck | Yes |
| 33 | SCC-15 | 910911 | Head and Neck | Head and Neck | Yes |
| 34 | SCC-25 | 910701 | Head and Neck | Head and Neck | Yes |
| 35 | SCC-4 | 910904 | Head and Neck | Head and Neck | Yes |
| 36 | SCC-9 | 909709 | Head and Neck | Head and Neck | Yes |
| 37 | SKN-3 | 1299059 | Head and Neck | Head and Neck | Yes |
| 38 | AU565 | 910704 | Breast | Breast | Yes |
| 39 | BT-20 | 906801 | Breast | Breast | Yes |
| 40 | BT-474 | 946359 | Breast | Breast | Yes |
| 41 | BT-483 | 949093 | Breast | Breast | Yes |
| 42 | BT-549 | 905951 | Breast | Breast | Yes |
| 43 | CAL-120 | 906826 | Breast | Breast | Yes |
| 44 | CAL-148 | 924106 | Breast | Breast | Yes |
| 45 | CAL-51 | 910927 | Breast | Kidney | No |
| 46 | CAL-85-1 | 910852 | Breast | Breast | Yes |
| 47 | CAMA-1 | 946382 | Breast | Breast | Yes |
| 48 | COLO-824 | 906812 | Breast | Breast | Yes |
| 49 | DU-4475 | 906844 | Breast | Colorectal | No |
| 50 | EFM-19 | 906851 | Breast | Breast | Yes |
| 51 | EFM-192A | 1290798 | Breast | Breast | Yes |
| 52 | EVSA-T | 906862 | Breast | Breast | Yes |
| 53 | HCC1143 | 749710 | Breast | Breast | Yes |
| 54 | HCC1187 | 749711 | Breast | Breast | Yes |
| 55 | HCC1395 | 749712 | Breast | Breast | Yes |
| 56 | HCC1419 | 907045 | Breast | Breast | Yes |
| 57 | HCC1428 | 1290905 | Breast | Breast | Yes |
| 58 | HCC1500 | 1303900 | Breast | Breast | Yes |
| 59 | HCC1569 | 907046 | Breast | Breast | Yes |
| 60 | HCC1599 | 749713 | Breast | Breast | Yes |
| 61 | HCC1806 | 907047 | Breast | Bladder | No |
| 62 | HCC1937 | 749714 | Breast | Breast | Yes |
| 63 | HCC1954 | 749709 | Breast | Breast | Yes |
| 64 | HCC202 | 1290906 | Breast | Breast | Yes |
| 65 | HCC2157 | 749715 | Breast | Breast | Yes |
| 66 | HCC2218 | 749716 | Breast | Breast | Yes |
| 67 | HCC38 | 749717 | Breast | Breast | Yes |
| 68 | HCC70 | 907048 | Breast | Breast | Yes |
| 69 | HDQ-P1 | 1290922 | Breast | Breast | Yes |
| 70 | MCF7 | 905946 | Breast | Breast | Yes |
| 71 | MDA-MB-157 | 925338 | Breast | Breast | Yes |
| 72 | MDA-MB-231 | 905960 | Breast | Lung | No |
| 73 | MDA-MB-330 | 1330941 | Breast | Breast | Yes |
| 74 | MDA-MB-361 | 908121 | Breast | Breast | Yes |
| 75 | MDA-MB-415 | 924240 | Breast | Breast | Yes |
| 76 | MDA-MB-436 | 1240172 | Breast | Skin | No |
| 77 | MDA-MB-453 | 908122 | Breast | Breast | Yes |
| 78 | MDA-MB-468 | 908123 | Breast | Breast | Yes |
| 79 | MFM-223 | 910948 | Breast | Breast | Yes |
| 80 | OCUB-M | 909256 | Breast | Breast | Yes |
| 81 | T47D | 905945 | Breast | Breast | Yes |
| 82 | UACC-812 | 910910 | Breast | Breast | Yes |
| 83 | UACC-893 | 909778 | Breast | Breast | Yes |
| 84 | ZR-75-30 | 909907 | Breast | Breast | Yes |
| 85 | CCK-81 | 1240123 | Colorectal | Colorectal | Yes |
| 86 | CL-11 | 1290769 | Colorectal | Colorectal | Yes |
| 87 | CL-40 | 1240124 | Colorectal | Colorectal | Yes |
| 88 | COLO-205 | 905961 | Colorectal | Colorectal | Yes |
| 89 | COLO-320-HSR | 910569 | Colorectal | Colorectal | Yes |
| 90 | COLO-678 | 910689 | Colorectal | Colorectal | Yes |
| 91 | CW-2 | 910554 | Colorectal | Colorectal | Yes |
| 92 | HCC2998 | 905971 | Colorectal | Colorectal | Yes |
| 93 | HCC-56 | 1290907 | Colorectal | Colorectal | Yes |
| 94 | HCT-116 | 905936 | Colorectal | Stomach | No |
| 95 | HCT-15 | 905937 | Colorectal | Colorectal | Yes |
| 96 | HT-115 | 907289 | Colorectal | Colorectal | Yes |
| 97 | HT-29 | 905939 | Colorectal | Colorectal | Yes |
| 98 | HT55 | 907287 | Colorectal | Colorectal | Yes |
| 99 | KM12 | 905989 | Colorectal | Colorectal | Yes |
| 100 | LS-1034 | 917486 | Colorectal | Colorectal | Yes |
| 101 | LS-123 | 907792 | Colorectal | Colorectal | Yes |
| 102 | LS-180 | 998189 | Colorectal | Colorectal | Yes |
| 103 | LS-411N | 907794 | Colorectal | Colorectal | Yes |
| 104 | LS-513 | 907795 | Colorectal | Colorectal | Yes |
| 105 | MDST8 | 1240173 | Colorectal | Skin | No |
| 106 | NCI-H630 | 908482 | Colorectal | Colorectal | Yes |
| 107 | NCI-H716 | 908458 | Colorectal | Colorectal | Yes |
| 108 | NCI-H747 | 908457 | Colorectal | Colorectal | Yes |
| 109 | RCM-1 | 909263 | Colorectal | Colorectal | Yes |
| 110 | RKO | 909698 | Colorectal | Colorectal | Yes |
| 111 | SK-CO-1 | 909718 | Colorectal | Colorectal | Yes |
| 112 | SNU-1040 | 1659823 | Colorectal | Colorectal | Yes |
| 113 | SNU-175 | 1659928 | Colorectal | Colorectal | Yes |
| 114 | SNU-407 | 1660034 | Colorectal | Colorectal | Yes |
| 115 | SNU-61 | 1660035 | Colorectal | Colorectal | Yes |
| 116 | SNU-81 | 1660036 | Colorectal | Colorectal | Yes |
| 117 | SNU-C1 | 910905 | Colorectal | Colorectal | Yes |
| 118 | SNU-C2B | 909740 | Colorectal | Stomach | No |
| 119 | SNU-C5 | 1674021 | Colorectal | Colorectal | Yes |
| 120 | SW1116 | 909746 | Colorectal | Colorectal | Yes |
| 121 | SW1417 | 909747 | Colorectal | Colorectal | Yes |
| 122 | SW1463 | 909748 | Colorectal | Colorectal | Yes |
| 123 | SW48 | 909751 | Colorectal | Colorectal | Yes |
| 124 | SW837 | 909755 | Colorectal | Colorectal | Yes |
| 125 | SW948 | 909757 | Colorectal | Colorectal | Yes |
| 126 | T84 | 909761 | Colorectal | Colorectal | Yes |
| 127 | C3A | 910850 | Liver | Liver | Yes |
| 128 | HLE | 907057 | Liver | Liver | Yes |
| 129 | JHH-1 | 1298151 | Liver | Kidney | No |
| 130 | JHH-6 | 1240159 | Liver | Liver | Yes |
| 131 | JHH-7 | 1240160 | Liver | Liver | Yes |
| 132 | SK-HEP-1 | 909719 | Liver | Lung | No |
| 133 | SNU-182 | 1240216 | Liver | Liver | Yes |
| 134 | SNU-387 | 909736 | Liver | Liver | Yes |
| 135 | SNU-398 | 1240217 | Liver | Liver | Yes |
| 136 | SNU-423 | 909737 | Liver | Liver | Yes |
| 137 | SNU-449 | 909738 | Liver | Liver | Yes |
| 138 | SNU-475 | 909739 | Liver | Liver | Yes |
| 139 | 769-P | 910922 | Kidney | Kidney | Yes |
| 140 | 786-0 | 905947 | Kidney | Kidney | Yes |
| 141 | A498 | 905948 | Kidney | Kidney | Yes |
| 142 | A704 | 910920 | Kidney | Kidney | Yes |
| 143 | ACHN | 905950 | Kidney | Kidney | Yes |
| 144 | BB65-RCC | 753533 | Kidney | Kidney | Yes |
| 145 | BFTC-909 | 910698 | Kidney | Kidney | Yes |
| 146 | CAKI-1 | 905963 | Kidney | Kidney | Yes |
| 147 | CAL-54 | 910952 | Kidney | Kidney | Yes |
| 148 | G-401 | 907299 | Kidney | Testis | No |
| 149 | HA7-RCC | 753558 | Kidney | Kidney | Yes |
| 150 | KMRC-1 | 1298168 | Kidney | Kidney | Yes |
| 151 | KMRC-20 | 1298169 | Kidney | Kidney | Yes |
| 152 | LB1047-RCC | 753577 | Kidney | Kidney | Yes |
| 153 | LB2241-RCC | 753578 | Kidney | Kidney | Yes |
| 154 | LB996-RCC | 753585 | Kidney | Kidney | Yes |
| 155 | NCC010 | 1509073 | Kidney | Kidney | Yes |
| 156 | NCC021 | 1509074 | Kidney | Kidney | Yes |
| 157 | OS-RC-2 | 909250 | Kidney | Kidney | Yes |
| 158 | RCC10RGB | 909974 | Kidney | Kidney | Yes |
| 159 | RCC-AB | 1524418 | Kidney | Kidney | Yes |
| 160 | RCC-ER | 1524417 | Kidney | Kidney | Yes |
| 161 | RCC-FG2 | 1524414 | Kidney | Kidney | Yes |
| 162 | RCC-JW | 1524416 | Kidney | Kidney | Yes |
| 163 | RCC-MF | 1524419 | Kidney | Kidney | Yes |
| 164 | RXF393 | 905978 | Kidney | Kidney | Yes |
| 165 | SN12C | 905979 | Kidney | Soft Tissue | No |
| 166 | TK10 | 905980 | Kidney | Kidney | Yes |
| 167 | U031 | 905981 | Kidney | Kidney | Yes |
| 168 | VMRC-RCZ | 909781 | Kidney | Kidney | Yes |
| 169 | 201T | 1287381 | Lung | Liver | No |
| 170 | A549 | 905949 | Lung | Liver | No |
| 171 | ABC-1 | 906791 | Lung | Lung | Yes |
| 172 | COR-L105 | 906805 | Lung | Liver | No |
| 173 | EKVX | 905970 | Lung | Esophagus | No |
| 174 | EMC-BAC-1 | 1503369 | Lung | Lung | Yes |
| 175 | EMC-BAC-2 | 1503370 | Lung | Liver | No |
| 176 | H3255 | 1247873 | Lung | Lung | Yes |
| 177 | HCC-44 | 1240145 | Lung | Lung | Yes |
| 178 | HCC-78 | 1290908 | Lung | Lung | Yes |
| 179 | HCC-827 | 1240146 | Lung | Lung | Yes |
| 180 | LXF-289 | 753592 | Lung | Lung | Yes |
| 181 | NCI-H1355 | 724866 | Lung | Liver | No |
| 182 | NCI-H1395 | 684681 | Lung | Liver | No |
| 183 | NCI-H1435 | 1298347 | Lung | Lung | Yes |
| 184 | NCI-H1563 | 753600 | Lung | Liver | No |
| 185 | NCI-H1568 | 1298348 | Lung | Lung | Yes |
| 186 | NCI-H1573 | 908472 | Lung | Lung | Yes |
| 187 | NCI-H1623 | 687798 | Lung | Lung | Yes |
| 188 | NCI-H1648 | 687799 | Lung | Lung | Yes |
| 189 | NCI-H1650 | 687800 | Lung | Breast | No |
| 190 | NCI-H1651 | 910900 | Lung | Liver | No |
| 191 | NCI-H1666 | 908473 | Lung | Lung | Yes |
| 192 | NCI-H1693 | 687802 | Lung | Skin | No |
| 193 | NCI-H1703 | 908474 | Lung | Skin | No |
| 194 | NCI-H1734 | 722058 | Lung | Lung | Yes |
| 195 | NCI-H1755 | 908475 | Lung | Adrenal Gland | No |
| 196 | NCI-H1781 | 1298350 | Lung | Lung | Yes |
| 197 | NCI-H1792 | 724868 | Lung | Lung | Yes |
| 198 | NCI-H1793 | 908463 | Lung | Liver | No |
| 199 | NCI-H1838 | 687807 | Lung | Lung | Yes |
| 200 | NCI-H1944 | 1240185 | Lung | Liver | No |
| 201 | NCI-H1975 | 924244 | Lung | Lung | Yes |
| 202 | NCI-H1993 | 908476 | Lung | Lung | Yes |
| 203 | NCI-H2009 | 724873 | Lung | Lung | Yes |
| 204 | NCI-H2023 | 1240187 | Lung | Liver | No |
| 205 | NCI-H2030 | 722045 | Lung | Liver | No |
| 206 | NCI-H2085 | 687812 | Lung | Lung | Yes |
| 207 | NCI-H2087 | 724834 | Lung | Lung | Yes |
| 208 | NCI-H2122 | 722046 | Lung | Lung | Yes |
| 209 | NCI-H2228 | 687816 | Lung | Lung | Yes |
| 210 | NCI-H2291 | 724874 | Lung | Lung | Yes |
| 211 | NCI-H23 | 905942 | Lung | Lung | Yes |
| 212 | NCI-H2342 | 687819 | Lung | Lung | Yes |
| 213 | NCI-H2347 | 687820 | Lung | Lung | Yes |
| 214 | NCI-H2405 | 687821 | Lung | Pancreas | No |
| 215 | NCI-H292 | 753604 | Lung | Bladder | No |
| 216 | NCI-H3122 | 1240190 | Lung | Lung | Yes |
| 217 | NCI-H322M | 905967 | Lung | Lung | Yes |
| 218 | NCI-H358 | 908465 | Lung | Lung | Yes |
| 219 | NCI-H441 | 908460 | Lung | Lung | Yes |
| 220 | NCI-H522 | 905944 | Lung | Liver | No |
| 221 | NCI-H596 | 908459 | Lung | Lung | Yes |
| 222 | NCI-H650 | 722066 | Lung | Lung | Yes |
| 223 | NCI-H838 | 910399 | Lung | Lung | Yes |
| 224 | PC-14 | 753608 | Lung | Lung | Yes |
| 225 | RERF-LC-KJ | 1298537 | Lung | Lung | Yes |
| 226 | RERF-LC-MS | 910931 | Lung | Skin | No |
| 227 | SK-LU-1 | 909721 | Lung | Liver | No |
| 228 | SW1573 | 724878 | Lung | Stomach | No |
| 229 | EBC-1 | 753554 | Lung | Lung | Yes |
| 230 | EPLC-272H | 753556 | Lung | Lung | Yes |
| 231 | HARA | 1240142 | Lung | Esophagus | No |
| 232 | HCC-15 | 1240143 | Lung | Lung | Yes |
| 233 | KNS-62 | 753569 | Lung | Lung | Yes |
| 234 | LK-2 | 687787 | Lung | Esophagus | No |
| 235 | LOU-NH91 | 1298226 | Lung | Breast | No |
| 236 | NCI-H1869 | 1240183 | Lung | Lung | Yes |
| 237 | NCI-H2170 | 687815 | Lung | Lung | Yes |
| 238 | NCI-H226 | 905941 | Lung | Adrenal Gland | No |
| 239 | NCI-H520 | 908443 | Lung | Lung | Yes |
| 240 | SK-MES-1 | 909728 | Lung | Lung | Yes |
| 241 | SW900 | 724879 | Lung | Lung | Yes |
| 242 | 42-MG-BA | 687561 | Brain | Soft Tissue | No |
| 243 | 8-MG-BA | 687562 | Brain | Brain | Yes |
| 244 | A172 | 687563 | Brain | Adrenal Gland | No |
| 245 | AM-38 | 910933 | Brain | Brain | Yes |
| 246 | CAS-1 | 910943 | Brain | Brain | Yes |
| 247 | CCF-STTG1 | 906823 | Brain | Brain | Yes |
| 248 | D-247MG | 946367 | Brain | Brain | Yes |
| 249 | D-263MG | 946368 | Brain | Brain | Yes |
| 250 | D-336MG | 946369 | Brain | Brain | Yes |
| 251 | D-392MG | 946370 | Brain | Brain | Yes |
| 252 | D-502MG | 946373 | Brain | Brain | Yes |
| 253 | D-542MG | 753549 | Brain | Brain | Yes |
| 254 | D-566MG | 946377 | Brain | Brain | Yes |
| 255 | DBTRG-05MG | 906835 | Brain | Brain | Yes |
| 256 | DK-MG | 906839 | Brain | Brain | Yes |
| 257 | GAMG | 906868 | Brain | Brain | Yes |
| 258 | GB-1 | 687568 | Brain | Brain | Yes |
| 259 | GI-1 | 906871 | Brain | Adrenal Gland | No |
| 260 | GMS-10 | 906873 | Brain | Brain | Yes |
| 261 | H4 | 907042 | Brain | Adrenal Gland | No |
| 262 | KALS-1 | 907271 | Brain | Brain | Yes |
| 263 | KINGS-1 | 907279 | Brain | Brain | Yes |
| 264 | KNS-42 | 907282 | Brain | Brain | Yes |
| 265 | KNS-81-FD | 924188 | Brain | Brain | Yes |
| 266 | KS-1 | 907313 | Brain | Soft Tissue | No |
| 267 | LN-18 | 1240168 | Brain | Brain | Yes |
| 268 | LN-229 | 1240169 | Brain | Skin | No |
| 269 | LN-405 | 910694 | Brain | Brain | Yes |
| 270 | LNZTA3WT4 | 1240170 | Brain | Brain | Yes |
| 271 | M059J | 949094 | Brain | Brain | Yes |
| 272 | MOG-G-CCM | 908144 | Brain | Brain | Yes |
| 273 | MOG-G-UVW | 908145 | Brain | Brain | Yes |
| 274 | NMC-G1 | 908449 | Brain | Brain | Yes |
| 275 | SF126 | 909712 | Brain | Soft Tissue | No |
| 276 | SF268 | 905986 | Brain | Brain | Yes |
| 277 | SF295 | 905985 | Brain | Brain | Yes |
| 278 | SF539 | 905984 | Brain | Soft Tissue | No |
| 279 | SK-MG-1 | 909729 | Brain | Adrenal Gland | No |
| 280 | SNB75 | 905982 | Brain | Brain | Yes |
| 281 | SW1088 | 909745 | Brain | Brain | Yes |
| 282 | SW1783 | 909750 | Brain | Brain | Yes |
| 283 | T98G | 687586 | Brain | Soft Tissue | No |
| 284 | U-118-MG | 687588 | Brain | Brain | Yes |
| 285 | U251 | 905983 | Brain | Brain | Yes |
| 286 | U-87-MG | 687590 | Brain | Brain | Yes |
| 287 | YH-13 | 909905 | Brain | Brain | Yes |
| 288 | YKG-1 | 687592 | Brain | Brain | Yes |
| 289 | CAPAN-1 | 753624 | Pancreas | Pancreas | Yes |
| 290 | CFPAC-1 | 906821 | Pancreas | Pancreas | Yes |
| 291 | DAN-G | 1290797 | Pancreas | Esophagus | No |
| 292 | HPAC | 1298136 | Pancreas | Esophagus | No |
| 293 | HPAF-II | 724869 | Pancreas | Stomach | No |
| 294 | KP-1N | 1298216 | Pancreas | Esophagus | No |
| 295 | KP-3 | 1298219 | Pancreas | Stomach | No |
| 296 | KP-4 | 753572 | Pancreas | Stomach | No |
| 297 | MZ1-PC | 753595 | Pancreas | Pancreas | Yes |
| 298 | PANC-02-03 | 1298475 | Pancreas | Pancreas | Yes |
| 299 | PANC-03-27 | 925346 | Pancreas | Pancreas | Yes |
| 300 | PANC-04-03 | 1298476 | Pancreas | Esophagus | No |
| 301 | PANC-08-13 | 925347 | Pancreas | Stomach | No |
| 302 | PANC-10-05 | 925348 | Pancreas | Stomach | No |
| 303 | PA-TU-8902 | 1298526 | Pancreas | Stomach | No |
| 304 | PA-TU-8988T | 1240201 | Pancreas | Stomach | No |
| 305 | PL18 | 1240208 | Pancreas | Kidney | No |
| 306 | PL4 | 1298533 | Pancreas | Pancreas | Yes |
| 307 | PSN1 | 910546 | Pancreas | Pancreas | Yes |
| 308 | QGP-1 | 1298534 | Pancreas | Prostate | No |
| 309 | SU8686 | 1240218 | Pancreas | Pancreas | Yes |
| 310 | SUIT-2 | 1240219 | Pancreas | Colorectal | No |
| 311 | SW1990 | 910907 | Pancreas | Pancreas | Yes |
| 312 | YAPC | 909904 | Pancreas | Pancreas | Yes |
| 313 | A101D | 910921 | Skin | Skin | Yes |
| 314 | A2058 | 906792 | Skin | Skin | Yes |
| 315 | A375 | 906793 | Skin | Skin | Yes |
| 316 | C32 | 906830 | Skin | Skin | Yes |
| 317 | CHL-1 | 910853 | Skin | Skin | Yes |
| 318 | COLO-679 | 906818 | Skin | Skin | Yes |
| 319 | COLO-792 | 906814 | Skin | Skin | Yes |
| 320 | COLO-800 | 906813 | Skin | Skin | Yes |
| 321 | COLO-829 | 687448 | Skin | Skin | Yes |
| 322 | CP50-MEL-B | 753545 | Skin | Skin | Yes |
| 323 | CP66-MEL | 753546 | Skin | Skin | Yes |
| 324 | G-361 | 906865 | Skin | Skin | Yes |
| 325 | GAK | 910932 | Skin | Skin | Yes |
| 326 | G-MEL | 1240130 | Skin | Skin | Yes |
| 327 | HMV-II | 907058 | Skin | Skin | Yes |
| 328 | HT-144 | 907067 | Skin | Skin | Yes |
| 329 | IGR-1 | 907169 | Skin | Skin | Yes |
| 330 | IGR-37 | 1240153 | Skin | Skin | Yes |
| 331 | IPC-298 | 907171 | Skin | Skin | Yes |
| 332 | IST-MEL1 | 907172 | Skin | Skin | Yes |
| 333 | LB2518-MEL | 753579 | Skin | Skin | Yes |
| 334 | LB373-MEL-D | 753581 | Skin | Skin | Yes |
| 335 | LOXIMVI | 905974 | Skin | Skin | Yes |
| 336 | M14 | 905975 | Skin | Skin | Yes |
| 337 | MEL-HO | 908124 | Skin | Skin | Yes |
| 338 | MEL-JUSO | 908125 | Skin | Skin | Yes |
| 339 | MMAC-SF | 925339 | Skin | Skin | Yes |
| 340 | MZ2-MEL | 971777 | Skin | Skin | Yes |
| 341 | RPMI-7951 | 910903 | Skin | Skin | Yes |
| 342 | RVH-421 | 909706 | Skin | Skin | Yes |
| 343 | SH-4 | 909713 | Skin | Skin | Yes |
| 344 | SK-MEL-1 | 909723 | Skin | Skin | Yes |
| 345 | SK-MEL-2 | 905955 | Skin | Skin | Yes |
| 346 | SK-MEL-24 | 909725 | Skin | Skin | Yes |
| 347 | SK-MEL-28 | 905954 | Skin | Skin | Yes |
| 348 | SK-MEL-3 | 909724 | Skin | Skin | Yes |
| 349 | SK-MEL-30 | 909726 | Skin | Skin | Yes |
| 350 | SK-MEL-31 | 909727 | Skin | Skin | Yes |
| 351 | SK-MEL-5 | 905956 | Skin | Skin | Yes |
| 352 | UACC-257 | 905977 | Skin | Skin | Yes |
| 353 | UACC-62 | 905976 | Skin | Skin | Yes |
| 354 | WM-115 | 909784 | Skin | Skin | Yes |
| 355 | WM1552C | 1299078 | Skin | Skin | Yes |
| 356 | WM278 | 1240226 | Skin | Skin | Yes |
| 357 | WM35 | 1299080 | Skin | Skin | Yes |
| 358 | WM793B | 1299081 | Skin | Skin | Yes |
| 359 | 639-V | 906798 | Bladder | Esophagus | No |
| 360 | 647-V | 906797 | Bladder | Bladder | Yes |
| 361 | BFTC-905 | 910926 | Bladder | Bladder | Yes |
| 362 | CAL-29 | 1290730 | Bladder | Bladder | Yes |
| 363 | DSH1 | 753552 | Bladder | Bladder | Yes |
| 364 | HT-1197 | 907065 | Bladder | Bladder | Yes |
| 365 | HT-1376 | 907066 | Bladder | Bladder | Yes |
| 366 | J82 | 753566 | Bladder | Bladder | Yes |
| 367 | KU-19-19 | 907312 | Bladder | Esophagus | No |
| 368 | LB831-BLC | 753584 | Bladder | Bladder | Yes |
| 369 | RT-112 | 909704 | Bladder | Bladder | Yes |
| 370 | RT4 | 687455 | Bladder | Bladder | Yes |
| 371 | SW1710 | 909749 | Bladder | Kidney | No |
| 372 | SW780 | 687457 | Bladder | Bladder | Yes |
| 373 | T-24 | 724812 | Bladder | Bladder | Yes |
| 374 | TCCSUP | 687459 | Bladder | Bladder | Yes |
| 375 | UM-UC-3 | 724838 | Bladder | Adrenal Gland | No |
| 376 | VM-CUB-1 | 909780 | Bladder | Bladder | Yes |
| 377 | NCCIT | 908441 | Testis | Testis | Yes |
| 378 | NEC8 | 910942 | Testis | Testis | Yes |
| 379 | DIFI | 1789883 | Colorectal | Colorectal | Yes |
| 380 | NCI-H508 | 908442 | Colorectal | Colorectal | Yes |
| 381 | SNU-283 | 1659929 | Colorectal | Colorectal | Yes |
| 382 | JHH-2 | 1240157 | Liver | Liver | Yes |
| 383 | JHH-4 | 1240158 | Liver | Liver | Yes |
| 384 | SW156 | 1240220 | Kidney | Kidney | Yes |
| 385 | NCI-H1437 | 687794 | Lung | Lung | Yes |
| 386 | D-423MG | 946372 | Brain | Brain | Yes |
| 387 | CP67-MEL | 949092 | Skin | Skin | Yes |
| 388 | VMRC-LCD | 713869 | Lung | Liver | No |
| 389 | YMB-1-E | 1303911 | Breast | Breast | Yes |
| 390 | D-245MG | 946366 | Brain | Brain | Yes |
