# Supplementary material for: Predicting cancer origins with a DNA methylation-based deep neural network model
Source: PLoS One. 2020 May 8;15(5):e0226461. doi: 10.1371/journal.pone.0226461 (PMC7209244; doi:10.1371/journal.pone.0226461)
Supplement: Attachment — Submitted filename: 2_Response to Reviewers_final_March27.docx [file pone.0226461.s009.docx]

Reviewer #1: Authors should make a case study which can use the method. For example, CTC methylation information could be used for showing the usefulness of the application. One of the example, CTC was used (PMID: 28389380).

*We agree with the reviewer that case studies to demonstrate the usefulness and potential applications of our approach is important. In our original manuscript, we have applied our model into independent dataset from GEO, which includes 581 tumor samples covering 10 tissue origins, demonstrating its usefulness in cancer origin prediction in solid tumors.*

*In this revision, we introduced another use case, i.e. cancer cell type prediction. As the reviewer suggested, CTC would be an exciting application. However, DNA methylation data from CTCs cells are limited. Instead, we did a literature search and found a paper that reported DNA methylation data from 1001 human cancer cell line (Iorio et al.). A total of 391 of them are covered in our 18-cancer tissue origin classifier. Applying our model in this dataset, our model achieved a good performance (precision: 86.13%, recall: 82.55%). We included the details for this application in Method, Results and Discussion in this revision. As high-quality cancer methylation data becomes increasingly publicly available in the future, we expect that we will be able to demonstrate more applications of our approaches.*

*Iorio F, Knijnenburg TA, Vis DJ, Bignell GR, Menden MP, Schubert M, et al. A Landscape of Pharmacogenomic Interactions in Cancer. Cell. 2016 Jul 28;166(3):740-754.*

Reviewer #2: General:

The authors proposed a deep neural network-based model to predict origin of cancers using DNA methylation data available in TCGA repository. They divided the dataset into train. development and test sets according to 60:20:20 splits. Other than using 10-fold cross-validation, they also used an independent set of data available in GEO database.

Strength:

• Author clearly mentioned that how cancer site classification study is useful

• Idea of model development and independent validation is good

• Prediction of metastatic samples provides another level of validation

Weakness:

• Major drawback of this model is that it cannot tell which features are responsible for classification or related to the origin of cancers.

*We thank reviewer for the insightful comments. We agree that our current model lacks interpretability in two ways: (1) the “black-box” nature of existing deep-leaning methods; (2) our limited understanding how methylation sites are related to biology of cancer origins.*

*In the revised manuscript, we discussed these two points in the Discussion Section.*

• Though the DNN model configuration is mentioned, the author did not specify which version of DNN is used, for example MLP, Autoencoder, etc.

*We used MLP in our model construction. In the revised manuscript, we clarified the choice of model configuration.*

• Design of DNN architecture was not explained clearly. How the number of hidden layers and number of nodes in each hidden layer are decided?

*Thanks for pointing out this. In the revised manuscript, we added more details as following “ three learning rates (α = 0.001, 0.01 and 0.1), three hidden layers (L = 2, 3, 4) and three hidden layer units (N = 32, 64, 128) were tested. We used grid search strategy to optimize these parameters and the best combination according to development set performance is α = 0.001, L = 2 and N = 64.”*

• Feature selection is the key component of this study, which was not explained clearly. Given the information provided, it would be difficult to reproduce the results

*Thanks for pointing out this. We added details for step-by-step feature selection in the revised manuscript.*

• There is no section with “Conclusion.”

*We added “Conclusion” section in this revised manuscript.*

Minor Comments:

• Line - 61: “All these molecular platforms have shown better performance in identifying tissue of origin as compared to pathology-based methods.”  This sentence does not make sense with the context of material presented in this section. Needs rephrasing.

*Thanks for this comment. We rephrased this sentence as following “Though these molecular platforms have shown better performance in identifying tissue of origin as compared to pathology-based methods, gene expression- or microRNA-bases classifiers are not easy to be implemented in clinic setting partially due to the instability of RNA.”*

• Line-62: “However, gene expression- or microRNA-bases classifiers need to handle RNA that is unstable and less convenient in clinic settings.” This statement requires some references to support. There is also a spelling mistake, should be … microRNA-based.

*Thanks for pointing out this mistake. It was corrected. The supporting references are also added as following “.. gene expression- or microRNA-bases classifiers are not easy to be implemented in clinic setting partially due to the instability of RNA [17, 18].”*

• Line – 114: “Then we used the Tukey honest test to remove….”  Little explanation of “Tukey honest test” and how it works would improve the quality of the paper.

*A detailed explanation for the Tukey honest test and how it was used were added in the revised method section as following:*

*“Tukey’s HSD is a multiple comparison procedure to find means that are significantly different from each other. It’s essentially a t-test that controls family-wise error rate, which is commonly used for post hoc test for ANOVA [35]. The results from Tukey’s HSD test are pairwise tissue comparisons with statistical significance and mean difference. Here, we used the pairwise tissue test results to obtain the maximal difference of the mean beta value among all tissues. Tukey’s HSD test resulted in 10360 CpG sites that were used for the input layer of neural network.”*

• Line-262: “High performance of our DNA methylation based DNN model may provide an opportunity in this scenario when pathology-based approach fails.”  But it does not provide which DNA methylation sites are related to producing high performance.

*In the revised manuscript, we made modifications as following “High performance of our DNA methylation-based DNN model may provide an opportunity in this scenario when pathology-based approach fails. However, compared to pathologic examination, DNA methylation-based DNN prediction models has limited interpretability due to the “black-box” nature of deep learning methods and our limited understanding of the mechanistic connections between DNA methylation and cancer origins. We envision that a hybrid approach innovatively combining existing pathological examinations with DNA methylation-based prediction may offer both interpretability and high prediction power.”*

…….

Major Comments:

• Need to add a section called “Conclusion”

*As the reviewer suggested, a “Conclusion” section was added*

• Feature Selection: Line-110: “To make the model with good compatibility and also reduce the dimensionality, we firstly reduced CpG sites to 27K for 450K derived samples.”  Concern-1: The authors did not mention what technique was used to reduce the dimension from 450K to 27K. Concern-2: After reducing features 450K to 27K, there will be two sets of 27K features. It is not clear – What do they do with these two sets? Do they combine? Step-by-step procedure to get to the final set of features, 10,360 CpG sites, needs to be stated/explained clearly so that the results can be reproduced.

*Thanks for this comment. A detailed feature selection procedure was described in the revised method.*

*For concern-1: This step was done by directly extracting 27K features from the 450K data as the probes used in 450K includes all probes in 27K. Our study data from TCGA and GEO were generated by 450 K platform.*

*For concern-2: Reducing 450K to 27K features is largely for reducing the number of features while at the same time making the set of features back-compatible with those from 27K platform. It is likely that reducing CpG sites to 27K for 450K may lead to information loss. As methylation data are increasingly generated by 450K platform and back-compatibility is no longer an issue, other cutoffs for feature size reduction can be used. We will investigate this issue in the future.*

• Line-127: “In addition, three hyperparameters (learning rate, number of hidden layer and hidden layer unit) were optimized to obtain best performance according to development set performance.”  More details are needed on how each of these hyperparameters are obtained so that the results can be reproduced.

*Thanks for this comment. A detailed hyperparameter tuning procedure was described in the revised manuscript as following: “We employed multilayer perceptron (MLP) to construct the neuron network. Three hyperparameters (learning rate, number of hidden layer and hidden layer unit) were optimized according to development set performance (1,468 patients with the same distribution of cancer origins as training set). Three learning rates (α = 0.001, 0.01 and 0.1), three hidden layers (L = 2, 3, 4) and three hidden layer units (N = 32, 64, 128) were tested. We used grid search strategy to optimize these parameters and the best combination according to development set performance is α = 0.001, L = 2 and N = 64.”*
